# The effect of higher versus lower protein delivery in critically ill patients: a systematic review and meta-analysis of randomized controlled trials

**DOI:** 10.1186/s13054-021-03693-4

**Published:** 2021-07-23

**Authors:** Zheng-Yii Lee, Cindy Sing Ling Yap, M. Shahnaz Hasan, Julia Patrick Engkasan, Mohd Yusof Barakatun-Nisak, Andrew G. Day, Jayshil J. Patel, Daren K. Heyland

**Affiliations:** 1 Department of Anesthesiology, Faculty of Medicine, University of Malaya, Kuala Lumpur, Malaysia; 2 Department of Rehabilitation Medicine, Faculty of Medicine, University of Malaya, Kuala Lumpur, Malaysia; 3 Department of Nutrition and Dietetics, Faculty of Medicine and Health Sciences, Universiti Putra Malaysia, Serdang, Malaysia; 4 Department of Critical Care Medicine, Queen’s University and the Clinical Evaluation Research Unit, Kingston General Hospital, Kingston, ON Canada; 5 Medical College of Wisconsin, Milwaukee, WI USA; 6 Institute for Social Science Studies, Universiti Putra Malaysia, Serdang, Malaysia

**Keywords:** Critical illness, Protein, Nutrition support, Muscle, Systematic review

## Abstract

**Background:**

The optimal protein dose in critical illness is unknown. We aim to conduct a systematic review of randomized controlled trials (RCTs) to compare the effect of higher versus lower protein delivery (with similar energy delivery between groups) on clinical and patient-centered outcomes in critically ill patients.

**Methods:**

We searched MEDLINE, EMBASE, CENTRAL and CINAHL from database inception through April 1, 2021.We included RCTs of (1) adult (age ≥ 18) critically ill patients that (2) compared higher vs lower protein with (3) similar energy intake between groups, and (4) reported clinical and/or patient-centered outcomes. We excluded studies on immunonutrition. Two authors screened and conducted quality assessment independently and in duplicate. Random-effect meta-analyses were conducted to estimate the pooled risk ratio (dichotomized outcomes) or mean difference (continuous outcomes).

**Results:**

Nineteen RCTs were included (*n* = 1731). Sixteen studies used primarily the enteral route to deliver protein. Intervention was started within 72 h of ICU admission in sixteen studies. The intervention lasted between 3 and 28 days. In 11 studies that reported weight-based nutrition delivery, the pooled mean protein and energy received in higher and lower protein groups were 1.31 ± 0.48 vs 0.90 ± 0.30 g/kg and 19.9 ± 6.9 versus 20.1 ± 7.1 kcal/kg, respectively. Higher vs lower protein did not significantly affect overall mortality [risk ratio 0.91, 95% confidence interval (CI) 0.75–1.10, *p* = 0.34] or other clinical or patient-centered outcomes. In 5 small studies, higher protein significantly attenuated muscle loss (MD −3.44% per week, 95% CI −4.99 to −1.90; *p* < 0.0001).

**Conclusion:**

In critically ill patients, a higher daily protein delivery was not associated with any improvement in clinical or patient-centered outcomes. Larger, and more definitive RCTs are needed to confirm the effect of muscle loss attenuation associated with higher protein delivery.

*PROSPERO registration number*: CRD42021237530

**Supplementary Information:**

The online version contains supplementary material available at 10.1186/s13054-021-03693-4.

## Introduction

Critical illness is associated with significant skeletal muscle wasting [1, 2]. Survivors of critical illness often have impaired muscle function, which is associated with physical disability and reduced quality of life (QOL) [3]. Exogenous administration of protein/amino acids *may* attenuate protein losses and aid in the recovery of critically ill patients [4, 5]. Unfortunately, the optimal protein dose for critically ill patients remains unknown and nutrition societies worldwide provide disparate recommendations (1.2 to 2.5 g/kg body weight) based on weak evidence [6–9], which suggests clinical equipoise exists for protein dose in critically ill patients [10].

Previous systematic reviews and meta-analysis evaluating optimal protein dose in critical illness draw different conclusions. Hoffer and Bistrian concluded that a protein dose of 2.0–2.5 g/kg normal body weight is safe and could be optimal for most critically ill patients while acknowledging poor quality evidence informs their conclusions [11]. Davies et al. included 14 RCTs that comprised of 3238 patients and found no relationship between protein delivered and mortality. However, the mean protein delivered between groups was 0.67 ± 0.38 g/kg/day versus (vs) 1.02 ± 0.42 g/kg/day. In addition, they included studies that tested immunonutrition, which may be plagued by an interaction effect [12]. Fetterplace et al. included 6 RCTs with 511 patients and were unable to conclude whether protein provision of ≥ 1.2 vs < 1.2 g/kg per day improves outcomes due to limited data [13]. The systematic reviews by Davies and Fetterplace included studies that had significant differences in calories between groups, which may limit interpretation of results as the confounding effect of calories intake cannot be excluded [12, 13]. Furthermore, since the publication of the last meta-analysis, several other RCTs have been published that were not included in these analyses [14, 15].

Due to aforementioned limitations, we aimed to perform an up-to-date systematic review with meta-analysis of RCTs to compare the effect of higher vs lower protein dose (with similar energy between groups) on clinical and patient-centered outcomes in critically ill patients.

## Methodology

This systematic review was performed in accordance to the 2020 Preferred Reporting Items for Systematic Reviews and Meta-Analyses (PRISMA) statement [16]. The protocol of this systematic review is available at https://www.criticalcarenutrition.com/systematic-reviews, which maintains systematic review and meta-analysis of topics related to critical care nutrition, and since 2003, has synthesized evidence for the critical care nutrition community [17]. The systematic review was registered in PROSPERO (CRD42021237530).

### Eligibility criteria

We included RCTs of (1) adult (age ≥ 18) critically ill patients (explicitly stated as such, or mechanically ventilated or if uncertain, the control group mortality had to be greater than 5%) that (2) compared protein doses with delivery via enteral (EN) formula, EN protein supplementation, parenteral nutrition (PN), or intravenous (IV) amino acids, (3) reported similar energy intake, and (4) reported clinical and/or patient-centered outcomes (Table 1). Studies of elective surgery patients or studies with only biochemical, metabolic, or nutritional outcomes were excluded. Studies that investigated the effect of a immunonutrition (e.g., glutamine or arginine) were also excluded.

**Table 1:** PICOS criteria for inclusion of studies

Parameter	Inclusion criteria
Population	Adult (age ≥ 18 years old) critically ill patients (mechanically ventilated or mortality of > 5% in the control group)
Intervention	Higher protein delivery through enteral formula, enteral protein supplementation, parenteral nutrition, or intravenous amino acids
Comparator	Lower protein delivery (similar calories delivery with the intervention group)
Outcomes	Clinical outcomes (mortality, infectious complication, duration of mechanical ventilation, length of ICU stay, length of hospital stay) and/or patient-centered outcomes (muscle mass, muscle strength, physical function, discharge destination and quality of life)
Study design	Randomized controlled trial

### Information source and search strategies

We systematically searched MEDLINE, EMBASE and CENTRAL (Cochrane Database of Systematic Reviews and the Cochrane Central Register of Controlled Trials) through OVID, and CINAHL (Cumulative Index to Nursing and Allied Health Literature) through EBSCOhost from database inception to April 1, 2021. No language restrictions were applied. Search strategies for all databases are available in the Additional File 1: ClinicalTrials.gov was also searched for ongoing studies.

### Study selection process

Search results were exported into Mendeley Desktop Version 1.19.8 (Elsevier) for screening and removal of duplicates. The detailed study selection process are available in the Additional file 1.

### Data collection process and data items

A standardized form was used for data abstraction and was completed by two authors independently (ZYL and CSYL). Disagreements were resolved by a third author (DKH).

For studies that reported median (Q1–Q3) for continuous outcomes, we contacted the author to obtain the mean and standard deviation (SD). If means and SDs were unavailable, we excluded those outcomes from the meta-analysis. For nutrition variables, the daily mean and SD of energy and protein delivery (the exact value) was obtained from the primary publication or the corresponding author. In some cases, precise estimate were unavailable as data were only presented in a graph and authors are unable to provide the exact value. In this case, amounts of nutrition delivery were estimated from the graph but not included in the meta-analysis. Protein and energy delivery from individual studies was pooled into a single mean and SD (by group) by using an online calculator [18]. To investigate the effect of protein dose on changes in muscle mass, we contacted all authors that reported muscle mass to calculate the percentage change in muscle mass between 2 measurements.

In one included study, only 2 out of the 3 groups randomized with similarities in energy and differences in protein dose were included in our meta-analysis [19]. This study also reported the nutritional delivery and LOS outcomes separately for traumatic brain injury (TBI) and non-TBI group, and these were pooled into a single mean and SD [18].

### Study quality and risk of bias assessment

Each included study was critically appraised in duplicate by two independent authors (ZYL and CSLY) by using the methodological quality scoring system that ranges from 0 to 14 points, where higher score indicates higher study quality (Additional file 2: Table S1). This quality assessment tool has been used in prior critical care nutrition systematic reviews and allows for comparisons of quality across topics and across time [20, 21]. A third senior author (DKH) was consulted if agreement could not be reached. A trial was considered a level I study if all 3 of the following criteria were fulfilled: (1) concealed randomization, (2) double-blinded (outcome adjudication must be blinded) and (3) conducted an intention-to-treat analysis. If any one of the above characteristics was unfulfilled, it was considered a level II study. We also appraised the quality of included studies by using the Revised Cochrane risk-of-bias (ROB2) tool for randomized trials for each evaluated outcome [22]. (More information is available in Additional file 1).

### Data analysis

All analyses were conducted using RevMan 5.4 (Cochrane IMS, Oxford, UK). For dichotomized outcomes, the pooled risk ratio (RR) was estimated by the DerSimonian and Laird random effect meta-analysis. For continuous outcomes, the random effect mean difference (MD) was estimated. The random effect model was chosen due to heterogeneity in study duration and protein doses between groups and in between studies. Heterogeneity was quantified by the I^2^ measure. Publication bias was evaluated by funnel plots. Egger’s test for funnel plot asymmetry was performed by using the metafor package in RStudio (version 1.3.1093) if ≥ 10 studies are included in a meta-analysis [23].

The following outcomes were pooled in the meta-analysis: (i) Nutritional outcomes: average protein (g/kg/day and g/day) and energy (kcal/kg/day and kcal/day) for the individual study duration; (ii) Clinical outcomes: overall mortality (if > 1 type of mortality was reported, they will be selected in the order of 28-day, hospital, ICU and other mortality), and ICU, hospital, 28-day, and ≥ 60-day mortality (the mortality with the longest duration was chosen), infectious complications, ICU and hospital LOS and duration of mechanical ventilation (MV); (iii) Muscle outcomes: percentage change of muscle mass and handgrip strength; (iv) Discharge to rehabilitation facility and (v) QOL physical measures.

For muscle mass, all studies reported quadriceps/thigh muscles and the percentage change of the quadriceps muscles between 2 measurements (baseline and end of the study muscle mass outcome follow-up) were meta-analyzed. Since the duration between 2 measurements ranged from 7 to 28 days, it was converted to percentage change per week. In addition, raw (unconverted) muscle mass data was meta-analyzed and presented as standardized MD.

Subgroup analyses were performed for studies that used EN or PN/IV amino acids strategy to optimize the difference in the protein dose between groups. A sensitivity analysis was conducted by excluding a study that had a marginal difference in calorie delivery between groups. This study incidentally led to small differences in calorie delivery between groups after the addition of amino acid supplement [24]. Two post-hoc subgroup analyses were performed to test the robustness of our findings: studies that started intervention ≤ 3 vs > 3 days of ICU admission, and studies that enrolled patients with head/brain pathology vs studies that enrolled heterogenous population. A *p*-value ≤ 0.05 was considered significant and values between > 0.05 but < 0.20 were considered a trend towards significance (for hypothesis-generating purpose).

## Results

### Study selection

Our search identified a total of 4220 records from MEDLINE (*n* = 1025), EMBASE (*n* = 1634), CENTRAL (*n* = 1158), CINAHL (*n* = 403). We also identified 44 records from websites (*n* = 5), personal files (*n* = 14), and citation screening (*n* = 25). The study selection process is shown in the PRISMA 2020 flow diagram (Additional file 3: Figure S1). Overall, we included 19 RCTs. The list of excluded studies and reasons for exclusion are presented in Additional file 2: Table S2. Sixty-two potential trials were identified on ClinicalTrials.gov. After screening, 21 were considered ongoing or unpublished related trials and are listed in Additional file 2: Table S3.

### Studies and patients characteristics

Nineteen RCTs totaling 1731 patients were included (sample size range: 14–474) [14, 15, 19, 24–39]. Study characteristics are summarized in Table 2. Seven studies were conducted in Europe [15, 25, 27, 30, 33, 35, 37], four in Australia [24, 26, 32, 39], four in Asia [14, 19, 28, 36], two in North America [31, 34], two in South America [29, 38]. Nine studies included mixed medical and surgical population [14, 24–27, 29, 30, 32, 39], four included patients with stroke or head injury [31, 33, 34, 36], one included only medical [38], one included only surgical patients [15], and population studied (medical/surgical) was unclear in four studies [19, 28, 35, 37]. One study included only overweight (BMI ≥ 25) [27], one included only obese (BMI ≥ 30) patients [35], and one included patients with non-oliguric acute renal failure requiring PN [37].

**Table 2:** Study Population, Nutrition Route and Timing of Intervention

Author, year (country)	*N*	Population	EN	PN	Start Intervention		Days on Intervention	
					High	Low	High	Low
1. Clifton 1985 (USA)	20	Severe head injury	EN only	–	Balance period: ~ 7–14d after injury	Balance period: ~ 7–14d after injury	~ 7d	~ 7d
2. Mesejo 2003 (Spain)	50	EN ≥ 5 days, APACHE II 10–25, BMI ≥ 30, no kidney/liver failure	EN only	–	Within 48 h of ICU admission	Within 48 h of ICU admission	5 d	5 d
3. Zhou 2006 (China)	51	Severe stroke with GCS < 12	EN only	–	≤ 5 days of acute stroke	≤ 5 days of acute stroke	– (up to 14d)	– (up to 14d)
4. Singer 2007 (Israel)	14	MV with non-oliguric acute renal failure and required PN	–	PN only	D2 of ICU admission	D2 of ICU admission	3d	3d
5. Rugeles 2013 (Columbia)	80	Medical, EN ≥ 96 h	EN only	Exclude patients that need PN	~ ≤ 48 of ICU admission	~ ≤ 48 of ICU admission	≥ 96 h (up to 7d)	≥ 96 h (up to 7d)
6. Doig 2015 (Australia)	474	Mixed, Stay ≥ 2d	Decide by the attending physician		D1-2 of ICU admission	D1-2 of ICU admission	At D7, n = 124 (Until ICU DC: ICU LOS 11.6d)	At D7, n = 120 (Until ICU DC: ICU LOS 10.7d)
7. Ferrie 2015 (Australia)	120	Mixed, ≥ 3d on PN	–	PN only	1 (1–2) d in ICU	1 (1–2) d in ICU	10.0 (6.8–14.0) d (up to 10d)	9.5 (7.0–13.5) d (up to 10d)
8. Jakob 2017 (Switzerland)	90	Mixed, EN ≥ 3d, stay ≥ 5d	EN first	PN is only allowed if intolerant to EN	Time to reach full kcal goal: 2.2 (0.8–3.7)d	Time to reach full kcal goal: 2.0 (1.3–2.7)d	5.0 (3.6–6.4)d (up to 10d)	7.0 (5.3–8.7)d (up to 10d)
9. Fetterplace 2018 (Australia)	60	Mixed, MV within 48 h and remained ≥ 72 h	EN first	PN is allowed at the discretion of treating physician	Time EN start: 13 ± 8 h	Time EN start: 20 ± 10 h	At D7, n = 15 (up to 15d)	At D7, n = 12 (up to 15d)
10. van Zanten 2018 (Netherlands)	44	Mixed, MV, BMI ≥ 25, EN ≤ 48 h- > 5d	EN first	SPN is allowed if necessary	D1-2 of ICU admission	D1-2 of ICU admission	At D10, n = 16 (up to 28d)	At D10, n = 13 (up to 28d)
11. Vega-Alava 2018 (Philippines)	40	MV, EN	EN only	–	Within 24 h of ICU admission	Within 24 h of ICU admission	–	–
12. Azevedo 2019 (Brazil)	120	Mixed, MV, Stay > 2d	EN first	SPN is allowed after 5 days if caloric goal not achieved	~ ≤ 3d in the ICU (IC to adjust caloric intake)	~ ≤ 3d in the ICU (IC to adjust caloric intake)	– (up to 14d)	– (up to 14d)
13. Danielis 2019 (Italy)	40	Mixed, MV within 12 h, BMI 18.5 to 30, no acute/chronic renal or hepatic failure	EN first	SPN allowed to make up the energy shortfall	Once admitted to the ICU and assessed for eligibility	Once admitted to the ICU and assessed for eligibility	– (up to end of MV, onset of acute renal or hepatic failure, transfer to another hospital or death)	
14. Badjatia 2020 (USA)	25	SAH, Stay > 7d, BMI 15 to 40	EN or oral intake	–	Time EN start: < 24 h of aneurysmal repair	Time EN start: < 24 h of aneurysmal repair	12 d (range 9–14)	12 d (range 9–14)
15. Bukhari 2020^ (Indonesia)	33	ICU patients not contraindicated or intolerant to EN	EN only	–	Within 24–48 h of ICU admission	Within 24–48 h of ICU admission	3d	3d
16. Chapple 2020 (Australia)	116	Mixed, MV, EN > 2d	EN first	SPN is allowed if deemed necessary by the treating physician	~ 19 h of ICU admission	~ 17.6 h of ICU admission	8.7 ± 7.3d (up to 28d)	8.1 ± 6.3d (up to 28d)
17. Nakamura 2020 (Japan)	117	Mixed, No lower limb injury, no die or discharge < D10	EN first	SPN is allowed to reach energy goal within 3d (no IV AA)	Time EN start: < 48 h ICU admission	Time EN start: < 48 h ICU admission	EN: 8 (5–9)d. Oral up to D10	EN: 8 (5–9)d. Oral up to D10
18. Carteron 2021 (France)	195	Brain injured (GCS < 8), expected MV > 48 h	EN only	–	Within 36 h of ICU admission	Within 36 h of ICU admission	At D10, n = 52 (up to 10d)	At D10, n = 60 (up to 10d)
19. Dresen 2021 (Germany)	42	Surgical, MV, after stay ≥ 10d, expected stay ≥ 30d	EN first	If nutrition target were not achieved within 24 h, initiate SPN	After ≥ 10 days in the ICU	After ≥ 10 days in the ICU	At D25, n = 15 (up to 28d)	At D25, n = 12 (up to 28d)

^This study has 3 groups: control (*n* = 22), high-protein polymeric (*n* = 19) and oligomeric group (*n* = 14), the control group was excluded from the analysis

*EN* enteral nutrition, *PN* parenteral nutrition, *d* day(s), *h* hour, *ICU* intensive care unit, *DC* discharge, *LOS* length of stay, *SPN* supplemental parenteral nutrition, *MV* mechanical ventilation, *BMI* body mass index, *IC* indirect calorimetry

Sixteen studies used an EN route and three used PN [37, 39] or IV amino acids [24] strategy to deliver protein. Of the 16 studies that used an EN strategy, supplemental PN was allowed in 8 studies. Sixteen studies started the intervention within 3 days of ICU admission. One study each started intervention within 5 days of acute stroke [36], 7–14 days after head injury [34] and after 10 days in the ICU [15]. The duration of intervention ranged 3 to 28 days. Sixteen studies used an EN delivery strategy and 9 of them achieved higher protein by using an EN formula with higher protein content [19, 25, 27, 30, 32–36]. Other patients’ baseline and nutritional characteristics can be found in Additional file 2: Tables S4 and S5.

### Energy estimation and nutrition prescription and delivery

Eleven studies (*n* = 908) reported weight-based nutrition delivery. The pooled mean protein delivery for the higher vs lower protein group were 1.31 ± 0.48 vs 0.90 ± 0.30 g/kg/day respectively, resulting in a daily MD of 0.48 g/kg (95% confidence interval [CI] 0.33–0.63, *p* < 0.00001; *I*^2^ = 92%) more protein delivery. Energy delivery was not different between groups (19.87 ± 6.93 vs. 20.13 ± 7.10 kcal/kg/day; MD −0.64, 95% CI −1.71 to 0.43, *p* = 0.24; *I*^2^ = 60%) (Additional file 3: Figure S2).

A total of 7 studies reported protein delivery in g/day and 8 studies reported energy delivery in kcal/day. The pooled mean protein delivery for the higher vs lower protein group were 97.2 ± 27.6 vs 68.7 ± 20.8 g/day respectively, resulting in a MD of 32.8 g/day (95% CI 18.5–47.1, *p* < 0.00001; *I*^2^ = 89%) more protein delivery. Energy delivery was not different between groups (1580.7 ± 484.1 vs 1555.0 ± 475.2 kcal/day; MD 41.7, 95% CI −38.8 to 122.2, *p* = 0.31; *I*^2^ = 20%) (Additional file 3: Figure S3).

### Assessment of study methodology

The median methodological quality score of included studies was 8 (out of 14). A total of 10 studies had a methodological quality score of ≥ 8 [15, 25–28, 31–34, 39]. Three trials were level 1 studies [26, 27, 39] (Additional file 2: Table S6). The ROB2 plots are presented in Additional file 3: Figure S4. Generally, most of the outcomes had some concerns, mainly due to biases arising from the randomization process and selection of the reported results.

### Outcomes

All relevant outcomes are summarized in Additional file 2: Table S7.

#### Mortality

**Fig. 1:**
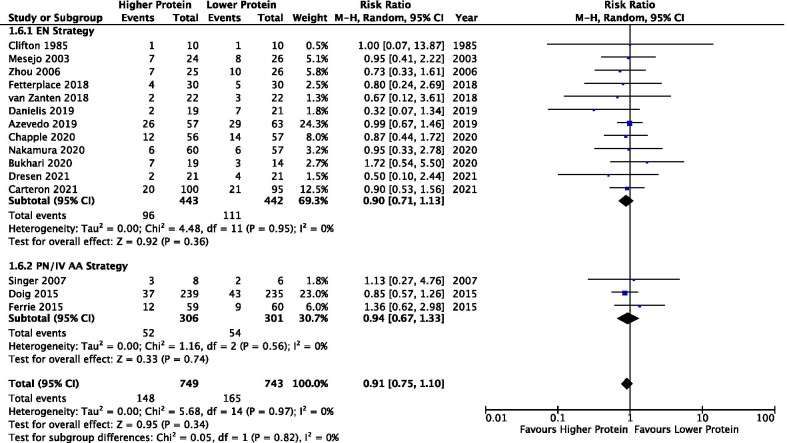
Overall mortality

A total of 15 studies reported mortality outcome (*n* = 1492). No difference was found between higher vs lower protein groups (RR 0.91, 95% CI 0.75–1.10, *p* = 0.34; *I*^2^ = 0%) in the overall analysis or the EN and PN/IV amino acids subgroups (test for subgroup differences *p* = 0.82; *I*^2^ = 0%) (Fig. 1). No evidence of funnel plot asymmetry was detected (*t* = −0.098, *p* = 0.924).

Nine studies (*n* = 1020) reported ICU mortality. No difference was found between groups in the overall (RR 0.94, 95% CI 0.74–1.20, *p* = 0.63; *I*^2^ = 0%) or subgroup analyses (test for subgroup differences *p* = 0.73; *I*^2^ = 0%). (Additional file 3: Figure S5a).

Five studies (n = 790) reported hospital mortality. No difference was found in the overall (RR 0.98, 95% CI 0.76–1.26, *p* = 0.80; *I*^2^ = 0%) or subgroup analyses (test of subgroup differences *p* = 0.76; *I*^2^ = 0%) Additional file 3: Figure S5b.

Seven studies (*n* = 622) reported 28-day mortality. All studies utilized EN strategy and 28-day mortality was not different between groups (RR 0.83, 95% CI 0.60–1.15, *p* = 0.26; *I*^2^ = 0%) Additional file 3: Figure S5c.

Seven studies (*n* = 1027) reported ≥ 60-day mortality. Of these, 60-day [26, 33], 3-month [34], 90-day [24, 32, 36] and 6-month [39] mortality were statistically aggregated. No difference was found in the overall (RR 0.99, 95% CI 0.78–1.24, *p* = 0.91; *I*^2^ = 0%) or subgroup analyses (test for subgroup differences *p* = 0.65; *I*^2^ = 0%) Additional file 3: Figure S5d.

#### Infectious complications

**Fig. 2:**
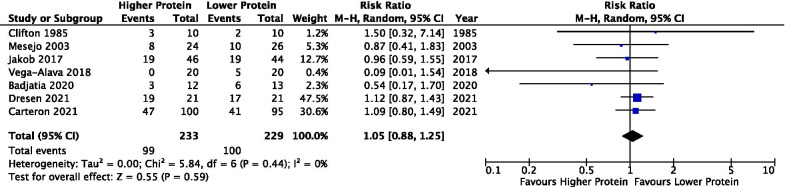
Infectious complications. Note: the infectious complications reported were not specified (Clifton 1985), Hospital-acquired infection (Mesejo 2003, Badjatia 2020), Secondary infection (Jakob 2017), ventilator-associated pneumonia (Vega-Alava 2018), Pneumonia (Carteron 2021), and Pneumonia in ICU (Dresen 2021)

Seven studies (*n* = 463) reported incidence of infection. All studies utilized EN strategy and there was no difference in infectious complications between groups (RR 1.05, 95% CI 0.88–1.25, *p* = 0.59; *I*^2^ = 0%) (Fig. 2).

#### Duration of mechanical ventilation

**Fig. 3:**
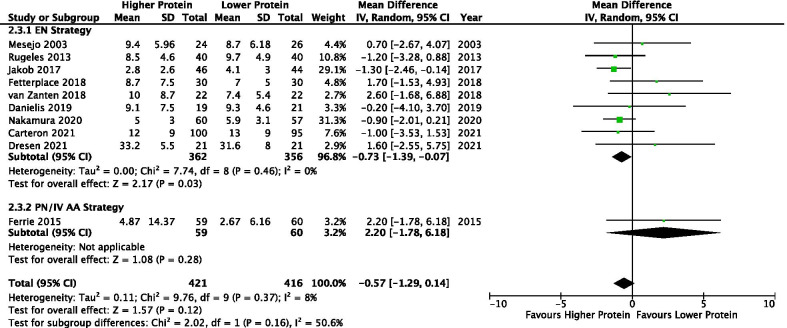
Duration of Mechanical Ventilation

Ten studies (*n* = 838) reported duration of MV. There was a trend towards a shorter duration of MV in patients that received higher compared to lower protein groups (MD −0.57, 95% CI −1.29 to 0.14, *p* = 0.12; *I*^2^ = 8%). In the EN subgroup, higher protein was associated with 0.73 less days on MV (MD −0.73, 95% CI −1.39 to −0.07, *p* = 0.03; *I*^2^ = 0%). One study utilized PN delivery strategy [39] and found no difference in duration of MV (MD 2.20, 95% CI −1.78 to 6.18, *p* = 0.28; test for subgroup differences, *p* = 0.16; *I*^2^ = 50.6%) (Fig. 3). Egger’s test found evidence of funnel plot asymmetry (*t* = 4.281, *p* = 0.003; Additional file 3: Figure S11).

#### Length of ICU and hospital stays

**Fig. 4:**
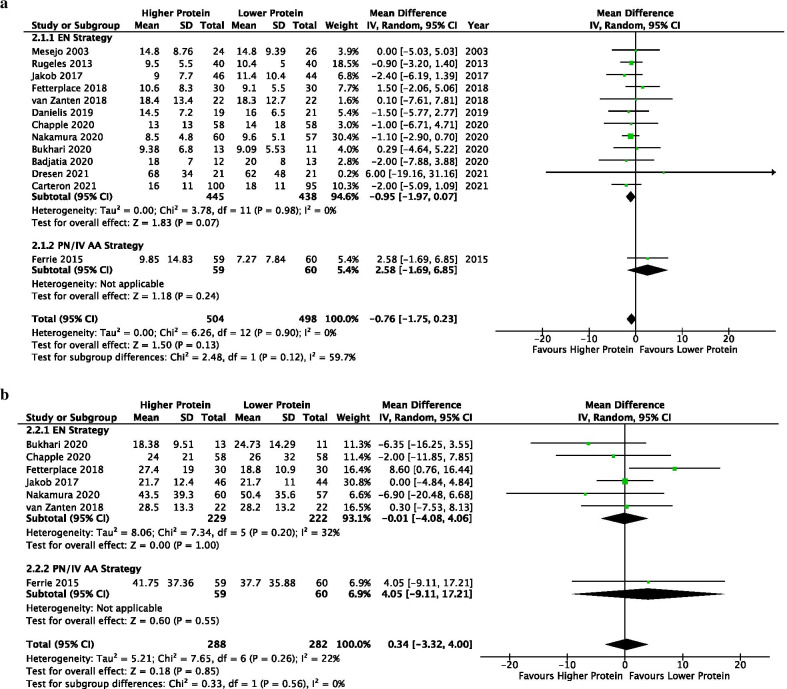
Length of Stays**.** **a** Length of ICU Stay. **b** Length of Hospital Stay

Thirteen studies (*n* = 1003) reported ICU LOS. There was a trend towards a shorter ICU LOS between higher vs lower protein groups (MD −0.76, 95% CI −1.75 to 0.23, *p* = 0.13; *I*^2^ = 0%). In the EN subgroup, higher protein was also associated with a trend towards shorter ICU LOS (MD −0.95, 95% CI −1.97 to 0.07, *p* = 0.07; *I*^2^ = 0%). One study utilized PN delivery strategy [39] and found no difference in ICU LOS (MD 2.58, 95% CI −1.69 to 6.85, *p* = 0.24; test for subgroup differences, *p* = 0.12; *I*^2^ = 59.7%) (Fig. 4a). No evidence of funnel plot asymmetry was detected (*t* = 1.086, *p* = 0.301; Additional file 3: Figure S11).

Seven studies (*n* = 570) were included for hospital LOS. There was no difference in hospital LOS between groups in the overall (MD 0.34, 95% CI -3.32 to 4.00, *p* = 0.85; *I*^2^ = 22%) or the subgroup analyses (test for subgroup differences *p* = 0.56; *I*^2^ = 0%) (Fig. 4b).

#### Muscle outcomes

Five studies (*n* = 273) reported quadriceps muscle and the percentage of muscle change per week between groups were statistically aggregated. (Table 3).

**Table 3:** Summary of muscle outcomes information

Study	Study duration		Protein, g/kg/d (*n*)		Energy, kcal/kg/d (*n*)		Device	Thigh area scanned	Compression	Was the assessor blinded?	Duration between two scans	Outcome	
Group	Higher	Lower	Higher	Lower	Higher	Lower						Higher	Lower
Ferrie 2015	10.0 (6.8–14.0) d	9.5 (7.0–13.5) d	1.09 ± 0.22 (59)	0.90 ± 0.21 (60)	23.1 ± 3.9 (59)	24.9 ± 4.2 (60)	US Sonosite M-Turbo; Fujifilm, Brookvale, NSW, Australia	Anteriorly at the mid-thigh point and two-thirds point of the thigh length*	Minimum	Yes	7d	Thigh area on D7, cm^2^ 6.8 ± 2.1 Change in thigh area on D7, % (*n* = 28) −8.4 ± 31.1^£^	Thigh area on D7, cm^**2**^ 5.8 ± 1.9 Change in thigh area on D7, % (*n* = 29) 30 ± 139.2^£^
Fetterplace 2018	At D7, *n* = 15 (up to 15d)	At D7, * n* = 12 (up to 15d)	1.2 ± 0.3 (30)	0.75 ± 0.11 (30)	23 ± 5.7 (30)	21 ± 3.3 (30)	US Sonosite S-ICU. Multiple-frequency transducer (13–6 MHz, 6 cm)	Midpoint and two-thirds between the anterior superior iliac spine and the upper pole of the patella	Maximum (sensitivity analysis of minimum showed the same results)^£^	Not clearly stated	ICU discharge (~10d) / 15d	QMLT loss, cm (*n* = 24) 12.73 ± 18.05^£^	QMLT loss, cm (*n* = 23) 21.25 ± 17.67^£^
Badjatia 2020	12 d (range 9–14)	12 d (range 9–14)	1.51 ± 0.47 (13)	0.88 ± 0.36 (12)	20 ± 7.1 (13)	19.8 ± 9.9 (12)	CT Siemens Somatom Sensation 64 Scanner	Started at the patella and ended at the femoral head	N/A	Yes	14d	Thigh muscle volume atrophy, % (*n* = 12) 6.5 ± 4.1	Thigh muscle volume atrophy, % (*n* = 13) 12.5 ± 6.4
Nakamura 2020	EN: 8 (5–9) d Oral up to day10	EN: 8 (5–9) d Oral up to D10	1.36 ± 0.8 (60)	0.72 ± 0.46 (57)	17.9 ± 10.1 (60)	16.2 ± 9.5 (57)	CT Scenaria; Hitachi Ltd., Tokyo, Japan)	Between the femoral head and patella	N/A	Yes	10d	Femoral muscle vol loss, % (*n* = 60) 12.9 ± 8.5	Femoral muscle vol loss, % (*n* = 57) 16.9 ± 7.0
Dresen 2021	At D25, *n* = 15 (up to 28 d) *(Enrolled patients after stayed* ≥ *10 days in the ICU)*	At D25, *n* = 12 (up to 28 d) *(Enrolled patients after stayed* ≥ *10 days in the ICU)*	1.5 ± 0.5 (21)	1.0 ± 0.4 (21)	27 ± 8.9 (21)	24.6 ± 9.8 (21)	US Philips HD15 PureWave Ultrasound system, Bothel, USA Philips ATL Linear 3–12 MHz probe' with the preset 'Abdominal 42 Hz'	(1) Upper two-thirds and the lower one third of the anterior superior iliac spine (ASIS) to the upper pole of the patella (2) Midpoint between the ASIS and the upper pole of the patella	Maximum	Yes	28days	Mean decrease of QMLT mid right, mid left, 2/3 right and 2/3 left, % (*n* = 15) 30.4 ± 11.7%	Mean decrease of QMLT mid right, mid left, 2/3 right and 2/3 left, % (*n* = 12) 51.8 ± 21.1%

^£^Information from the author; *Thigh length was measured from the superior point of the greater trochanter to the knee circumference point (the superior point of the lateral border of the tibia head) and the mid-thigh point and two-thirds point near the knee identified

*CT* computed tomography, d day, *g* gram, *ICU* intensive care unit, *kg* kilogram, *NA* not applicable, *US* ultrasound

**Fig. 5:**
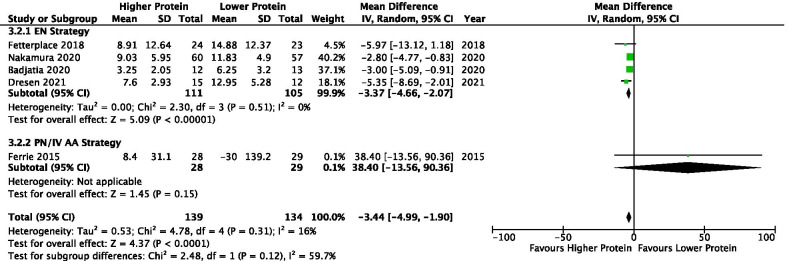
Percentage of muscle change per week

Higher, as compared to lower, protein delivery significantly attenuated muscle loss (MD −3.44% per week, 95% CI −4.99 to −1.90, *p* < 0.0001; *I*^2^ = 16%) (Fig. 5). This result is mainly attributed to the EN subgroup (MD −3.37, 95% CI −4.66 to −2.07, *p* < 0.00001; *I*^2^ = 0%). No difference was found in one study that utilized PN (MD 38.40, 95% CI −13.56 to 90.36, *p* = 0.15; test for subgroup differences, *p* = 0.12; *I*^2^ = 59.7%). The difference in protein and energy delivery between groups in the 5 studies were 0.46 g/kg/day (95% CI 0.26 to 0.65; *p* < 0.00001) and 0.56 kcal/kg/day (95% CI −1.68 to 2.81; *p* = 0.62), respectively (Additional file 3: Figure S6). Analysis with the raw (unconverted) percentage loss in the standardized scale showed less muscle loss (SMD −0.52, 95% CI −1.03 to −0.00, *p* = 0.05; *I*^2^ = 73%) in the higher protein group (Additional file 3: Figure S7) that was again largely driven by the signal in the EN subgroup analysis (SMD −0.68, 95% CI −1.02 to −0.34, *p* < 0.0001; *I*^2^ = 22%). Subjective evaluation of the funnel plots found no evidence of asymmetry (Additional file 3: Figure S11).

#### Muscle strength, discharge to rehabilitation facility and QOL

A total of 2, 3 and 4 studies that reported on muscle strength, discharge to rehabilitation facility and QOL physical measures respectively were statistically aggregated. These results are presented in Additional file 3: Figure S8–S10. No significant differences were found between groups for these outcomes.

### Sensitivity and post-hoc subgroup analysis

No change in the direction of results in a sensitivity analysis that exclude a study [24] that had different calories between groups after the addition of amino acids supplements (Additional file 2: Table S8). All post-hoc subgroup analyses had similar findings as the main analysis with the exception that higher protein improved quality of life physical measures in a very small study among patients with head/brain pathology (SMD 0.81, 95% CI −0.01 to 1.63, *p* = 0.05; test for subgroup differences *p* = 0.04; *I*^2^ = 75.2%) (Additional file 2: Table S8).

## Discussion

### Summary of main findings

In this systematic review and meta-analysis of RCTs that compared higher vs lower protein delivery (with similar energy delivery between groups) in critically ill patients, we found that a 0.48 g/kg/day higher protein delivery had no significant effect on overall mortality and other clinical and patient-centered outcomes. However, higher protein was associated with a trend towards shorter duration of MV and ICU LOS. In subgroup of studies that used an EN delivery strategy, higher protein was associated with a significantly shorter duration of MV (0.73 days) and about 1 day reduction in ICU length of stay (*p* = 0.07). In 5 small studies, higher protein delivery was associated with significant attenuation of muscle loss by 3.4% per week.

### Clinical outcomes

The results of this systematic review and meta-analysis are too imprecise to confirm a clinical benefit from higher protein administration. There are observational studies that suggest higher protein administration is beneficial and may be harmful. In a single-center study, Allingstrup et al. found protein delivery in the highest tertile (~ 1.46 g/kg), compared with the lowest tertile (~ 0.79 g/kg), was associated with lower ICU mortality [40]. In another large prospective observational study of 2828 patients who stayed in the ICU ≥ 4 days, receiving > 80% prescribed protein (1.2 g/kg) was associated with reduced mortality [5]. The mortality benefits of higher protein was independent of energy delivery in both studies. Although we found no differences in mortality outcomes, there were signal towards improvement in other clinical outcomes (trend towards shorter duration of MV and ICU LOS, particularly in the EN RCTs).

Our results do not align well with findings from several post-hoc analyses of RCTs and observational studies that suggest higher protein administration may be harmful. The post-hoc analysis of the EPANIC trial found that every 7 g/day higher cumulative protein delivery during the first 3 days of ICU admission was associated with a significantly lower likelihood of earlier ICU discharge [41]. However, 16 of 19 RCTs included in our meta-analysis started the protein intervention within 3 days of ICU admission without evidence of harmful effect. The disparate finding may be due to the patient population studied and method of protein delivery. The EPANIC study enrolled mostly cardiac surgery patients and tested supplemental PN. We found no differences in outcomes in the subgroup of patients who received PN as a protein delivery strategy.

In the PROTINVENT retrospective study, 6-month mortality was higher among patients who received > vs < 0.8 g/kg/day during the first 3 days of ICU admission. Between days 4 and 7, patients who received 0.8–1.2 g/kg/day of protein had a lower mortality than patients who received > 1.2 g/kg/day [42]. The post-hoc analysis of the INTACT trial found that every 1 g/kg higher protein delivery during the first week of ICU stay was associated with an increased risk for mortality, while higher protein after 7 days was associated with a decreased risk for mortality [43]. Similarly, Lew et al. found every 10% increase in goal protein delivery in patients with short-term nutritional support (≤ 6 days) was associated with increased 28-day mortality, but was associated with decreased mortality in patients requiring longer nutritional support [44]. These findings suggest optimal dose of protein may depend on the timing of protein delivery and that progressive increase in protein dose during the first week of critical illness may be associated with improved outcomes. These findings are not supported by our results where 17 of the RCTs started intervention within the first week (16 started within 3 days) and found no mortality difference between higher vs lower protein dose. Similar results were found in subgroup analyses of studies that started intervention ≤ 3 or > 3 days of ICU admission. The protein delivered of the higher protein group of the included RCTs ranges from 0.9 g/kg/day to 2.63 g/kg/day (most of them in the interval 1.2 to 1.5 g/kg/day). Altogether, we could not detect any harmful effect of higher protein during the early phase of critical illness as suggested by previous post-hoc analyses of RCTs and observational studies.

### Muscle, physical function and QOL outcomes

One important finding of this systematic review is that a 0.46 g/kg/day higher protein delivery was associated with muscle loss attenuation by 3.4% per week. Our findings align with results of a recent systematic review among healthy and non-critically ill patients that showed a dose–response relationship between protein and muscle [45]. Every 0.1 g/kg/day increment of protein intake was associated with an increase of 0.39 kg lean body mass, up to 1.3 g/kg/day [45]. Beyond 1.3 g/kg/day, the rate of increment of lean body mass continue to rise with resistance training and declined without training [45]. It is a coincidence that the higher protein group of our included studies received a pooled mean of 1.31 g/kg/d of protein, hence we are unsure whether a protein dosage of higher than 1.3 g/kg/d will confer additional muscle attenuating benefits. Taken together, these results suggest that a combination of higher protein and early resistance exercise may have an additive benefits for the critically ill patients [46, 47].

Our findings differ with several previous studies. Lambell et al. in a systematic review that included 4 observational studies and 2 RCTs that measured skeletal muscle mass and/or total body protein at ≥ 2 time points during critical illness found no association between energy and protein delivery and changes in skeletal muscle mass [48]. In another study, the same group found that the marked losses of computed tomography–derived skeletal muscle area and density over the first month of critical illness are not associated with energy and protein delivery [49]. While Puthucheary et al. in an observational study demonstrated that increasing protein delivery was associated with increased muscle wasting at day 10 within the limit of average daily protein delivery of 0.67 g/kg/day [50]. Our findings from RCTs do not support the results from these studies that are mainly observational.

The benefits of attenuation of muscle loss can be viewed from both shorter and longer-term. In the shorter term, our previous observational study demonstrated every 1% muscle loss (quadriceps muscle layer thickness [QMLT]) attenuation was independently associated with an odd of 0.95 for 60-day mortality [2]. In the longer-term, better self-reported physical function at 3 months was associated with greater QMLT at hospital discharge and at 3 months in a cohort of ICU patients with TBI [51]. Another study of survivors of acute respiratory distress syndrome found that a greater lean mass percentage was associated with gait speed and 6-min walk distance [52]. Taken together, the attenuation of muscle loss in the ICU not only may improve short-term survival, but also have the potential to have a legacy effect to the survivorship of a patient. Unfortunately, our meta-analysis was unable to demonstrate benefit on survival or QOL with higher protein delivery, and this warrant further study.

### Strengths and limitations

This study has several strengths. Firstly, we only included RCTs that delivered different protein but similar calories between group, a priori removing the potential confounding of calorie. We also did not include studies that used immune-modulating formula where the independent effect of glutamine or arginine may modify the effect on outcomes. Authors were contacted extensively to obtain relevant data.

One major limitation of this systematic review is that most of the included studies were of moderate quality, small and single-center. Notably, muscle mass outcomes are derived from 5 small studies and the risk of bias was high in 1 study and some concerns in 3 studies. Although 2 studies used computed tomography (CT) imaging and 3 studies used ultrasound (with different protocol) to measure muscle mass, this was standardized by calculating the percentage change of muscle mass (Table 3). CT is the gold standard for skeletal muscle mass assessment [53], while ultrasound is widely used in the critical care literature with excellent reliability [54, 55]. The finding of shorter duration of MV associated with higher protein delivery in the EN subgroup is weak due to possible biases as evidence by funnel plot asymmetry. In addition, this time-dependent variable is not normally distributed and further reduce the strength of this finding. Furthermore, our meta-analysis of approximately 1000 patients might still be underpowered to detect a difference in ≥ 60-day mortality [56]. Large and adequately powered trials such as the EFFORT trial (NCT 03160547) are ongoing to answer this important question.

## Conclusion

In critically ill patients, a 0.48 g/kg higher protein delivery (with similar calories delivery between groups) started within 3 days of ICU admission and last for 3 to 28 days in the ICU was not associated with a significant effect on overall mortality and mortality at any time point, duration of MV, ICU and hospital LOS or infectious complications. In subgroup analysis that used EN strategy to increase protein delivery, a significant shorter duration of MV (0.73 day) was shown; however, this finding may subject to possible biases as evidence by funnel plot asymmetry. In 5 small studies, higher protein delivery was associated with attenuated muscle mass loss (3.4% per week). Nevertheless, this was not translated to improve muscle strength, discharge destination and quality of life; however, very few trials reported these endpoints. The pooled protein and energy delivery was 1.31 ± 0.48 g/kg vs 0.90 ± 0.30 g/kg and 19.9 ± 6.9 kcal/kg vs 20.1 ± 7.1 kcal/kg in the higher versus lower protein group, respectively. Further studies are required to confirm these findings.

Future protein trials should focus on patient-centered outcome such as physical function and QOL outcomes [46]. In addition, the combination of higher protein with early mobilization might show a greater effect [46, 47]. Currently, a total of 21 RCTs are ongoing to investigate the effect of higher vs lower protein dosing in critical illness, and 6 of them combined a higher protein with early mobility/resistance exercise (Additional file 2: Table S2). More results will be forthcoming that will continue to shape our knowledge about the role of protein administration in the context of critical illness.

## Supplementary Information

**Additional file 1**. Supplementary methods and search strategies.

**Additional file 2**. Supplementary tables.

**Additional file 3**. Supplementary figures.

## Data Availability

All data generated and/or analyzed during the current study are included within the published article and its additional files.

## Abbreviations

BMI: Body mass index
CENTRAL: Cochrane Central Register of Controlled Trials
CI: Confidence interval
CINAHL: Cumulative Index to Nursing and Allied Health Literature
CT: Computed tomography
EN: Enteral nutrition
g: Gram
ICU: Intensive care unit
IV: Intravenous
kcal: Kilocalories
kg: Kilogram
LOS: Length of stay
MD: Mean difference
MV: Mechanical ventilation
PN: Parenteral nutrition
PRISMA: Preferred Reporting Items for Systematic Reviews and Meta-Analyses
Q1: Quartile 1
Q3: Quartile 3
QMLT: Quadriceps muscle layer thickness
QOL: Quality of life
RCT: Randomized controlled trial
ROB2: Revised Cochrane risk-of-bias tool
RR: Risk ratio
SD: Standard deviation
SMD: Standardized mean difference
TBI: Traumatic brain injury
vs: Versus
