# Supplementary material for: The effect of higher versus lower protein delivery in critically ill patients: a systematic review and meta-analysis of randomized controlled trials
Source: Crit Care. 2021 Jul 23;25:260. doi: 10.1186/s13054-021-03693-4 (PMC8300989; doi:10.1186/s13054-021-03693-4)
Supplement: Supplementary file 1 — Additional file 1. Supplementary methods and search strategies. [file 13054_2021_3693_MOESM1_ESM.docx]

**Supplementary methods**

**The Effect of Higher versus Lower Protein Delivery In Critically Ill Patients. A Systematic Review and Meta-Analysis of Randomized Controlled Trials.**

Zheng-Yii Lee, MSc^1^ [zheng_yii@hotmail.com](mailto:zheng_yii@hotmail.com)

Cindy Sing Ling Yap, BSc^1^ [cindyapsl@gmail.com](mailto:cindyapsl@gmail.com)

M. Shahnaz Hasan, MBBS, MAnaes^1^ [shahnaz@ummc.edu.my](mailto:shahnaz@ummc.edu.my)

Julia Patrick Engkasan, MBBS, MRehabMed, PhD^2^ [julia@ummc.edu.my](mailto:julia@ummc.edu.my)

Mohd Yusof Barakatun-Nisak, PhD^3,4^ [bnisak@upm.edu.my](mailto:bnisak@upm.edu.my)

Andrew G. Day, MSc^5^ [andrew.day@kingstonhsc.ca](mailto:andrew.day@kingstonhsc.ca)

Jayshil J. Patel, MD^6^ [jpatel2@mcw.edu](mailto:jpatel2@mcw.edu)

Daren K. Heyland, MSc, FRCPC^5^ [dkh2@queensu.ca](mailto:dkh2@queensu.ca)

^1^ Department of Anesthesiology, Faculty of Medicine, University of Malaya, Kuala Lumpur, Malaysia.

^2^ Department of Rehabilitation Medicine, Faculty of Medicine, University of Malaya, Kuala Lumpur, Malaysia.

^3^ Department of Nutrition and Dietetics, Faculty of Medicine and Health Sciences, Universiti Putra Malaysia, Serdang, Malaysia

^4^ Institute for Social Science Studies, Universiti Putra Malaysia, Selangor, Malaysia

^5^ Department of Critical Care Medicine, Queen’s University and the Clinical Evaluation Research Unit, Kingston General Hospital, Kingston, Ontario, Canada.

^6^ Medical College of Wisconsin, Milwaukee, Wisconsin, Unites States

**Corresponding Author**

Daren K. Heyland

Department of Critical Care Medicine, Queen’s University and the Clinical Evaluation Research Unit, Kingston General Hospital, Kingston, Ontario, Canada.

Email address: [dkh2@queensu.ca](mailto:dkh2@queensu.ca)

**Methods (Additional information)**

**Information Source and Search Strategies**

The search was conducted with 4 major concepts: (1) Critically ill population, (2) Nutritional Support, (3) Protein, and (4) Randomized Controlled Trial (by using the Cochrane highly sensitive search strategy for identifying randomized trials in MEDLINE, EMBASE and CINAHL).^1^ Each concept was search by using subject headings and relevant keywords and these were combined with the Boolean operator ‘OR’. The concepts were then combined with Boolean operator ‘AND’. Search strategies for all databases are presented below.

**Study Selection Process**

Search results were exported into Mendeley Desktop Version 1.19.8 (Elsevier) for removal of duplicates and then screened for irrelevant studies by using title or keyword search (e.g., observational study, retrospective, pediatrics, immunonutrition, etc.). One author (ZYL) screened the title and/or abstract of remaining articles to further exclude studies. The remaining studies were retrieved and assessed for eligibility by two authors (ZYL and DKH). The reference lists of previous systematic reviews, website (criticalcarenutrition.com), and personal files were also searched and potential eligible studies were retrieved and assessed for eligibility by two authors (ZYL and DKH). The number of excluded articles were recorded in each step and the PRISMA flow diagram was generated by the PRISMA2020 R package and ShinyApp.^2^

**Study Quality and Risk of Bias Assessment**

The Cochrane revised risk of bias (ROB2) contains 5 domains that include the risk of bias related to the randomization process, deviations from the intended interventions, missing outcomes, measurement of outcome, and selection of the reported outcome. The overall ROB2 assessment was categorized as low risk of bias, some concerns, or high risk of bias.^3^ The risk-of-bias traffic light and summary plots were generated by the Risk-of-bias VISualization (robvis) tool.^4^

**Search Strategies**

**1) Ovid MEDLINE(R), Ovid MEDLINE(R) Daily and Epub Ahead of Print, In-Process & Other Non-Indexed Citations**

| 1 | randomized controlled trial.pt. | 526,072 |
| --- | --- | --- |
| 2 | controlled clinical trial.pt. | 94,113 |
| 3 | randomized.ab. | 514,773 |
| 4 | placebo.ab. | 216,621 |
| 5 | drug therapy.fs. | 2,295,161 |
| 6 | randomly.ab. | 354,230 |
| 7 | trial.ab. | 545,841 |
| 8 | groups.ab. | 2,173,918 |
| 9 | or/1-8 | 4,955,500 |
| 10 | exp animals/ not humans.sh. | 4,807,801 |
| 11 | 9 not 10 | 4,307,993 |
| 12 | Critical care/ | 54,309 |
| 13 | critical care.mp. | 76,201 |
| 14 | intensive care.mp. | 187,806 |
| 15 | Critical illness/ | 31,328 |
| 16 | critical illness.mp. | 36,661 |
| 17 | critically ill.mp. | 48,565 |
| 18 | Intensive care units/ or burn units/ or coronary care units/ or respiratory care units/ | 65,097 |
| 19 | intensive care unit*.mp. | 157,471 |
| 20 | critical care unit*.mp. | 3,499 |
| 21 | exp Shock/ | 77,437 |
| 22 | sepsis/ or bacteremia/ or fungemia/ | 87,705 |
| 23 | systemic inflammatory response syndrome.mp. | 8,938 |
| 24 | sepsis.mp. | 130,992 |
| 25 | septic shock.mp. | 23,734 |
| 26 | multiple organ dysfunction syndrome.mp. | 2,337 |
| 27 | multiple organ failure.mp. | 16,207 |
| 28 | cytokine release syndrome.mp. | 2,049 |
| 29 | Respiratory Distress Syndrome/ | 20,816 |
| 30 | Severe Acute Respiratory Syndrome/ | 5,499 |
| 31 | respiratory distress syndrome.mp. | 47,312 |
| 32 | acute lung injury.mp. | 15,893 |
| 33 | Burns/ | 45,196 |
| 34 | (burn$ adj3 patient$).mp. | 15,840 |
| 35 | Multiple Trauma/ | 12,875 |
| 36 | Multi* Trauma.mp. | 15,027 |
| 37 | Abdominal Injuries/ | 14,681 |
| 38 | Abdom*n* injur*.mp. | 16,269 |
| 39 | Pancreatitis, Acute Necrotizing/ | 3,404 |
| 40 | Acute Necroti*ing Pancreatitis.mp. | 1,184 |
| 41 | brain injuries/ or brain hemorrhage, traumatic/ or brain injuries, diffuse/ or brain injuries, traumatic/ | 60,112 |
| 42 | Head Injuries, Closed/ | 3,113 |
| 43 | Brain Injur*.mp. | 97,670 |
| 44 | Head injur*.mp. | 27,932 |
| 45 | or/12-44 | 678,743 |
| 46 | nutritional support/ | 6,515 |
| 47 | enteral nutrition/ | 20,174 |
| 48 | parenteral nutrition/ | 15,532 |
| 49 | Parenteral Nutrition, Total/ | 10,197 |
| 50 | Intubation, Gastrointestinal/ | 9,811 |
| 51 | (nutrition$ adj3 support$).mp. | 17,441 |
| 52 | artificial nutrition.mp. | 1,281 |
| 53 | enteral nutrition.mp. | 23,879 |
| 54 | enteric feeding.mp. | 82 |
| 55 | parenteral nutrition.mp. | 32,438 |
| 56 | intravenous feeding.mp. | 460 |
| 57 | Gastrointestin* intubation.mp. | 52 |
| 58 | or/46-57 | 69,293 |
| 59 | Amino Acids/ | 138,604 |
| 60 | Peptides/ | 160,463 |
| 61 | Proteins/ or exp dietary proteins/ or protein hydrolysates/ | 305,686 |
| 62 | Protein*.mp. | 4,869,922 |
| 63 | amino acid*.mp. | 938,070 |
| 64 | peptide*.mp. | 838,166 |
| 65 | or/59-64 | 5,415,556 |
| 66 | 11 and 45 and 58 and 65 | 1,025 |

**2) EBM Reviews - Cochrane Central Register of Controlled Trial, Cochrane Database of Systematic Reviews**

| 1 | Critical care/ | 1,748 |
| --- | --- | --- |
| 2 | critical care.mp. | 4,669 |
| 3 | intensive care.mp. | 27,413 |
| 4 | Critical illness/ | 2,295 |
| 5 | critical illness.mp. | 3,885 |
| 6 | critically ill.mp. | 7,976 |
| 7 | Intensive care units/ or burn units/ or coronary care units/ or respiratory care units/ | 2,540 |
| 8 | intensive care unit*.mp. | 21,698 |
| 9 | critical care unit*.mp. | 408 |
| 10 | exp Shock/ | 2,328 |
| 11 | sepsis/ or bacteremia/ or fungemia/ | 2,832 |
| 12 | systemic inflammatory response syndrome.mp. | 1,242 |
| 13 | sepsis.mp. | 13,326 |
| 14 | septic shock.mp. | 3,471 |
| 15 | multiple organ dysfunction syndrome.mp. | 282 |
| 16 | multiple organ failure.mp. | 1,678 |
| 17 | cytokine release syndrome.mp. | 253 |
| 18 | Respiratory Distress Syndrome/ | 1,373 |
| 19 | Severe Acute Respiratory Syndrome/ | 330 |
| 20 | respiratory distress syndrome.mp. | 6,184 |
| 21 | acute lung injury.mp. | 1,410 |
| 22 | Burns/ | 1,446 |
| 23 | (burn$ adj3 patient$).mp. | 2,496 |
| 24 | Multiple Trauma/ | 221 |
| 25 | Multi* Trauma.mp. | 635 |
| 26 | Abdominal Injuries/ | 127 |
| 27 | Abdom*n* injur*.mp. | 292 |
| 28 | Pancreatitis, Acute Necrotizing/ | 119 |
| 29 | Acute Necroti*ing Pancreatitis.mp. | 84 |
| 30 | brain injuries/ or brain hemorrhage, traumatic/ or brain injuries, diffuse/ or brain injuries, traumatic/ | 2,138 |
| 31 | Head Injuries, Closed/ | 78 |
| 32 | Brain Injur*.mp. | 7,328 |
| 33 | Head injur*.mp. | 1,975 |
| 34 | or/1-33 | 60,720 |
| 35 | nutritional support/ | 246 |
| 36 | enteral nutrition/ | 1,807 |
| 37 | parenteral nutrition/ | 977 |
| 38 | Parenteral Nutrition, Total/ | 762 |
| 39 | Intubation, Gastrointestinal/ | 671 |
| 40 | (nutrition$ adj3 support$).mp. | 3,202 |
| 41 | artificial nutrition.mp. | 116 |
| 42 | enteral nutrition.mp. | 4,661 |
| 43 | enteric feeding.mp. | 1,718 |
| 44 | parenteral nutrition.mp. | 4,554 |
| 45 | intravenous feeding.mp. | 138 |
| 46 | Gastrointestin* intubation.mp. | 12 |
| 47 | or/35-46 | 11,115 |
| 48 | Amino Acids/ | 1,756 |
| 49 | Peptides/ | 1,794 |
| 50 | Proteins/ or exp dietary proteins/ or protein hydrolysates/ | 4,539 |
| 51 | Protein*.mp. | 108,926 |
| 52 | amino acid*.mp. | 9,963 |
| 53 | peptide*.mp. | 25,577 |
| 54 | or/48-53 | 132,984 |
| 55 | 34 and 47 and 54 | 1,158 |

**3) Embase Classic+Embase**

| 1 | Randomized controlled trial/ | 658,765 |
| --- | --- | --- |
| 2 | Controlled clinical study/ | 468,092 |
| 3 | random$.ti,ab. | 1,671,928 |
| 4 | randomization/ | 91,207 |
| 5 | intermethod comparison/ | 271,274 |
| 6 | placebo.ti,ab. | 328,587 |
| 7 | (compare or compared or comparison).ti. | 567,848 |
| 8 | ((evaluated or evaluate or evaluating or assessed or assess) and (compare or compared or comparing or comparison)).ab. | 2,300,046 |
| 9 | (open adj label).ti,ab. | 86,905 |
| 10 | ((double or single or doubly or singly) adj (blind or blinded or blindly)).ti,ab. | 249,982 |
| 11 | double blind procedure/ | 186,253 |
| 12 | parallel group$1.ti,ab. | 27,405 |
| 13 | (crossover or cross over).ti,ab. | 112,151 |
| 14 | ((assign$ or match or matched or allocation) adj5 (alternate or group$1 or intervention$1 or patient$1 or subject$1 or participant$1)).ti,ab. | 355,901 |
| 15 | (assigned or allocated).ti,ab. | 419,505 |
| 16 | (controlled adj7 (study or design or trial)).ti,ab. | 380,674 |
| 17 | (volunteer or volunteers).ti,ab. | 263,815 |
| 18 | human experiment/ | 544,718 |
| 19 | trial.ti. | 335,420 |
| 20 | or/1-19 | 5,457,077 |
| 21 | (random$ adj sampl$ adj7 (cross section$ or questionnaire$1 or survey$ or database$1)).ti,ab. not (comparative study/ or controlled study/ or randomi?ed controlled.ti,ab. or randomly assigned.ti,ab.) | 8,632 |
| 22 | Cross-sectional study/ not (randomized controlled trial/ or controlled clinical study/ or controlled study/ or randomi?ed controlled.ti,ab. or control group$1.ti,ab.) | 264,817 |
| 23 | (((case adj control$) and random$) not randomi?ed controlled).ti,ab. | 18,458 |
| 24 | (Systematic review not (trial or study)).ti. | 171,726 |
| 25 | (nonrandom$ not random$).ti,ab. | 17,071 |
| 26 | Random field$.ti,ab. | 2,506 |
| 27 | (random cluster adj3 sampl$).ti,ab. | 1,361 |
| 28 | (review.ab. and review.pt.) not trial.ti. | 883,661 |
| 29 | we searched.ab. and (review.ti. or review.pt.) | 36,224 |
| 30 | update review.ab. | 116 |
| 31 | (databases adj4 searched).ab. | 42,004 |
| 32 | (rat or rats or mouse or mice or swine or porcine or murine or sheep or lambs or pigs or piglets or rabbit or rabbits or cat or cats or dog or dogs or cattle or bovine or monkey or monkeys or trout or marmoset$1).ti. and animal experiment/ | 1,112,142 |
| 33 | Animal experiment/ not (human experiment/ or human/) | 2,337,224 |
| 34 | or/21-33 | 3,699,272 |
| 35 | 20 not 34 | 4,856,498 |
| 36 | Intensive care/ | 130,796 |
| 37 | critical care.mp. | 59,626 |
| 38 | intensive care.mp. | 386,690 |
| 39 | critical illness/ | 31,305 |
| 40 | critical illness.mp. | 37,797 |
| 41 | critically ill.mp. | 84,748 |
| 42 | intensive care unit/ or burn unit/ or coronary care unit/ or medical intensive care unit/ or neurological intensive care unit/ or stroke unit/ or surgical intensive care unit/ | 196,877 |
| 43 | intensive care unit*.mp. | 257,600 |
| 44 | critical care unit*.mp. | 6,227 |
| 45 | exp shock/ | 154,602 |
| 46 | systemic inflammatory response syndrome/ | 12,835 |
| 47 | sepsis/ or bacteremia/ or fungemia/ or septicemia/ or urosepsis/ | 237,200 |
| 48 | multiple organ failure/ | 41,280 |
| 49 | systemic inflammatory response syndrome.mp. | 15,656 |
| 50 | sepsis.mp. | 235,750 |
| 51 | septic shock.mp. | 64,763 |
| 52 | multiple organ dysfunction syndrome.mp. | 3,360 |
| 53 | multiple organ failure.mp. | 44,885 |
| 54 | cytokine release syndrome.mp. | 4,459 |
| 55 | respiratory distress syndrome/ or acute lung injury/ or adult respiratory distress syndrome/ or transfusion related acute lung injury/ | 70,901 |
| 56 | Severe Acute Respiratory Syndrome/ | 9,958 |
| 57 | respiratory distress syndrome.mp. | 76,966 |
| 58 | acute lung injury.mp. | 26,028 |
| 59 | burn/ or burn shock/ | 64,730 |
| 60 | (burn$ adj3 patient$).mp. | 22,924 |
| 61 | Multiple Trauma/ | 15,739 |
| 62 | Multi* Trauma.mp. | 18,064 |
| 63 | exp abdominal injury/ | 185,119 |
| 64 | Abdom*n* injur*.mp. | 18,430 |
| 65 | pancreatitis/ or acute hemorrhagic pancreatitis/ or acute pancreatitis/ | 95,705 |
| 66 | Acute Necroti*ing Pancreatitis.mp. | 1,964 |
| 67 | brain injury/ or acquired brain injury/ or brain concussion/ or brain contusion/ or brain damage/ or brain stem injury/ or cerebellum injury/ or diffuse brain injury/ or traumatic brain injury/ | 195,948 |
| 68 | head injury/ | 55,115 |
| 69 | Brain Injur*.mp. | 172,590 |
| 70 | Head injur*.mp. | 66,430 |
| 71 | or/36-70 | 1,462,314 |
| 72 | nutritional support/ | 20,268 |
| 73 | exp artificial feeding/ | 90,323 |
| 74 | (nutrition$ adj3 support$).mp. | 31,064 |
| 75 | artificial nutrition.mp. | 2,119 |
| 76 | enteral nutrition.mp. | 16,820 |
| 77 | enteric feeding.mp. | 34,669 |
| 78 | parenteral nutrition.mp. | 53,445 |
| 79 | intravenous feeding.mp. | 1,752 |
| 80 | Gastrointestin* intubation.mp. | 130 |
| 81 | or/72-80 | 118,882 |
| 82 | Amino Acids/ | 187,870 |
| 83 | Peptides/ | 122,756 |
| 84 | exp protein diet/ | 7,862 |
| 85 | protein intake/ | 45,636 |
| 86 | Protein*.mp. | 6,347,497 |
| 87 | amino acid*.mp. | 877,305 |
| 88 | peptide*.mp. | 828,078 |
| 89 | or/82-88 | 6,931,615 |
| 90 | 35 and 71 and 81 and 89 | 1,634 |

**4) CINAHL**

| S1 | MH randomized controlled trials | 113,840 |
| --- | --- | --- |
| S2 | MH double‐blind studies | 50,061 |
| S3 | MH single‐blind studies | 14,742 |
| S4 | MH random assignment | 66,921 |
| S5 | MH pretest‐posttest design | 45,690 |
| S6 | MH cluster sample | 4,696 |
| S7 | TI (randomised OR randomized) | 113,839 |
| S8 | AB (random*) | 333,577 |
| S9 | TI (trial) | 115,883 |
| S10 | MH (sample size) AND AB (assigned OR allocated OR control) | 4,144 |
| S11 | MH (placebos) | 12,816 |
| S12 | PT (randomized controlled trial) | 127,533 |
| S13 | AB (control W5 group) | 119,139 |
| S14 | MH (crossover design) OR MH (comparative studies) | 379,704 |
| S15 | AB (cluster W3 RCT) | 379 |
| S16 | MH animals+ | 94,979 |
| S17 | MH (animal studies) | 134,110 |
| S18 | TI (animal model*) | 3,250 |
| S19 | S16 OR S17 OR S18 | 220,725 |
| S20 | MH (human) | ####### |
| S21 | S19 NOT S20 | 191,413 |
| S22 | S1 OR S2 OR S3 OR S4 OR S5 OR S6 OR S7 OR S8 OR S9 OR S10 OR S11 OR S12 OR S13 OR S14 OR S15 | 830,547 |
| S23 | S22 NOT S21 | 790,537 |
| S24 | MH Critical Care | 23,807 |
| S25 | "critical care" | 57,350 |
| S26 | "intensive care" | 109,060 |
| S27 | MH Critical Illness | 13,204 |
| S28 | "critical illness" | 15,632 |
| S29 | ""critically ill"" | 34,032 |
| S30 | (MH "Intensive Care Units") OR (MH "Coronary Care Units") OR (MH "Post Anesthesia Care Units") OR (MH "Respiratory Care Units") OR (MH "Stroke Units") | 43,744 |
| S31 | "intensive care unit*" | 84,697 |
| S32 | "critical care unit*" | 2,446 |
| S33 | (MH "Multiple Organ Dysfunction Syndrome+") | 16,799 |
| S34 | (MH "Sepsis") OR (MH "Bacteremia") OR (MH "Fungemia+") | 23,836 |
| S35 | "systemic inflammatory response syndrome" | 2,620 |
| S36 | "sepsis" | 31,013 |
| S37 | ""septic shock"" | 8,605 |
| S38 | "multiple organ dysfunction syndrome" | 3,418 |
| S39 | "multiple organ failure" | 2,527 |
| S40 | "cytokine release syndrome" | 291 |
| S41 | (MH "Respiratory Distress Syndrome") OR (MH "Respiratory Distress Syndrome, Acute") OR (MH "Acute Lung Injury+") | 12,709 |
| S42 | (MH "Severe Acute Respiratory Syndrome") | 2,464 |
| S43 | "respiratory distress syndrome" | 14,435 |
| S44 | "acute lung injury" | 3,684 |
| S45 | (MH "Burns") | 15,141 |
| S46 | (MH "Burn Units") OR (MH "Burn Patients") | 2,908 |
| S47 | ""Burn* N3 patient*"" | 8,257 |
| S48 | "burn patient" | 1,124 |
| S49 | (MH "Multiple Trauma") | 3,394 |
| S50 | "Multi* Trauma" | 3,976 |
| S51 | (MH "Abdominal Injuries") | 2,662 |
| S52 | "Abdom*n* injur*" | 3,099 |
| S53 | (MH "Pancreatitis, Acute Necrotizing") | 372 |
| S54 | "Acute Necroti*ing Pancreatitis" | 122 |
| S55 | (MH "Brain Injuries") OR (MH "Brain Concussion") OR (MH "Brain Contusions") OR (MH "Left Hemisphere Injuries") OR (MH "Right Hemisphere Injuries") | 29,603 |
| S56 | (MH "Head Injuries") | 7,430 |
| S57 | "Brain Injur*" | 34,201 |
| S58 | "Head injur*" | 10,966 |
| S59 | S24 OR S25 OR S26 OR S27 OR S28 OR S29 OR S30 OR S31 OR S32 OR S33 OR S34 OR S35 OR S36 OR S37 OR S38 OR S39 OR S40 OR S41 OR S42 OR S43 OR S44 OR S45 OR S46 OR S47 OR S48 OR S49 OR S50 OR S51 OR S52 OR S53 OR S54 OR S55 OR S56 OR S57 OR S58 | 267,529 |
| S60 | (MH "Nutritional Support") OR (MH "Enteral Nutrition") OR (MH "Parenteral Nutrition") OR (MH "Peripheral Parenteral Nutrition") OR (MH "Total Parenteral Nutrition") | 19,459 |
| S61 | “”nutrition* N3 support*”” | 10,422 |
| S62 | "artificial nutrition" | 3,718 |
| S63 | "enteral nutrition" | 11,586 |
| S64 | "enteric feeding" | 22 |
| S65 | "parenteral nutrition" | 9,719 |
| S66 | "intravenous feeding" | 4,662 |
| S67 | (MH "Intubation, Gastrointestinal") | 2,074 |
| S68 | "Gastrointestin* intubation" | 5 |
| S69 | S60 OR S61 OR S62 OR S63 OR S64 OR S65 OR S66 OR S67 OR S68 | 26,779 |
| S70 | (MH "Amino Acids") | 9,680 |
| S71 | (MH "Peptides") | 16,779 |
| S72 | (MH "Proteins") OR (MH "Dietary Proteins") | 88,609 |
| S73 | "Protein*" | 265,749 |
| S74 | "amino acid*" | 18,814 |
| S75 | "peptide*" | 57,927 |
| S76 | S70 OR S71 OR S72 OR S73 OR S74 OR S75 | 308,026 |
| S77 | S23 AND S59 AND S69 AND S76 | 403 |

**References**

1. Lefebvre C, Glanville J, Briscoe S, et al. Technical Supplement to Chapter 4: Searching for and selecting studies. In: Higgins JPT, Thomas J, Chandler J, et al., eds. *Cochrane Handbook for Systematic Reviews of Interventions Version 6.1 (Updated September 2020)*. Cochrane, 2020. Available from www.training.cochrane.org/handbook.

2. Haddaway NR, McGuinness LA. PRISMA2020: R package and ShinyApp for producing PRISMA 2020 compliant flow diagrams (Version 0.0.1). 2020. doi:10.5281/zenodo.4287835

3. Sterne JAC, Savović J, Page MJ, et al. RoB 2: a revised tool for assessing risk of bias in randomised trials. *BMJ*. 2019;366:l4898-l4898. doi:10.1136/bmj.l4898

4. McGuinness LA, Higgins JPT. Risk-of-bias VISualization (robvis): An R package and Shiny web app for visualizing risk-of-bias assessments. *Res Syn Meth*. 2021;12:55-61. doi:10.1002/jrsm.1411
