# Supplementary material for: The effect of higher versus lower protein delivery in critically ill patients: a systematic review and meta-analysis of randomized controlled trials
Source: Crit Care. 2021 Jul 23;25:260. doi: 10.1186/s13054-021-03693-4 (PMC8300989; doi:10.1186/s13054-021-03693-4)
Supplement: Supplementary file 2 — Additional file 2. Supplementary tables. [file 13054_2021_3693_MOESM2_ESM.docx]

**Supplementary tables**

**The Effect of Higher versus Lower Protein Delivery In Critically Ill Patients. A Systematic Review and Meta-Analysis of Randomized Controlled Trials.**

Zheng-Yii Lee, MSc^1^ [zheng_yii@hotmail.com](mailto:zheng_yii@hotmail.com)

Cindy Sing Ling Yap, BSc^1^ [cindyapsl@gmail.com](mailto:cindyapsl@gmail.com)

M. Shahnaz Hasan, MBBS, MAnaes^1^ [shahnaz@ummc.edu.my](mailto:shahnaz@ummc.edu.my)

Julia Patrick Engkasan, MBBS, MRehabMed, PhD^2^ [julia@ummc.edu.my](mailto:julia@ummc.edu.my)

Mohd Yusof Barakatun-Nisak, PhD^3,4^ [bnisak@upm.edu.my](mailto:bnisak@upm.edu.my)

Andrew G. Day, MSc^5^ [andrew.day@kingstonhsc.ca](mailto:andrew.day@kingstonhsc.ca)

Jayshil J. Patel, MD^6^ [jpatel2@mcw.edu](mailto:jpatel2@mcw.edu)

Daren K. Heyland, MSc, FRCPC^5^ [dkh2@queensu.ca](mailto:dkh2@queensu.ca)

^1^ Department of Anesthesiology, Faculty of Medicine, University of Malaya, Kuala Lumpur, Malaysia.

^2^ Department of Rehabilitation Medicine, Faculty of Medicine, University of Malaya, Kuala Lumpur, Malaysia.

^3^ Department of Nutrition and Dietetics, Faculty of Medicine and Health Sciences, Universiti Putra Malaysia, Serdang, Malaysia

^4^ Institute for Social Science Studies, Universiti Putra Malaysia, Selangor, Malaysia

^5^ Department of Critical Care Medicine, Queen’s University and the Clinical Evaluation Research Unit, Kingston General Hospital, Kingston, Ontario, Canada.

^6^ Medical College of Wisconsin, Milwaukee, Wisconsin, Unites States

**Corresponding Author**

Daren K. Heyland

Department of Critical Care Medicine, Queen’s University and the Clinical Evaluation Research Unit, Kingston General Hospital, Kingston, Ontario, Canada.

Email address: [dkh2@queensu.ca](mailto:dkh2@queensu.ca)

**Table S1 Methodological Quality Scoring System**

|  |  | | | | | | |
| --- | --- | --- | --- | --- | --- | --- | --- |
|  | **Score** | | | | | | |
|  | **0** | | **1** | | | **2** | |
| Randomization |  | | Not concealed or not sure |  | | Concealed* randomization |  |
| Analysis | Other |  |  | | | Intention to treat |  |
| Blinding | Not blinded |  | Single blinded  *Check who was blinded:*  Health Care Professionals  Outcomes Assessors | |  | Double blinded |  |
| Patient selection | Selected patients or unable to tell |  | Consecutive eligible patients | |  |  | |
| Comparability of groups at baseline | No or not sure |  | Yes | |  |  | |
| Extent of follow-up | < 100% |  | 100% | |  |  | |
| Treatment protocol | Poorly described |  | Reproducibly described | |  |  | |
| Co-interventions** | Not described |  | Described but not equal or not sure | |  | Well described and all equal |  |
| Outcomes | Not described |  | Partially described | |  | Objectively defined |  |

**Total Score:** **(max 14)**

* Concealed randomization means the person enrolling the patients is unaware of the next treatment

assignment (e.g. phone in randomization, computer generated).

** Extent to which antibiotics, TPN, ventilation, oxygen, transfusions, etc were applied equally across groups

**Table S2: List of Excluded Studies, Reason for Exclusion and Study Source**

|  | Abel RM, Beck CH Jr, Abbott WM, Ryan JA Jr, Barnett GO, Fischer JE. Improved survival from acute renal failure after treatment with intravenous essential L-amino acids and glucose. Results of a prospective, double-blind study. *N Engl J Med*. 1973;288(14):695-699. doi:10.1056/NEJM197304052881401 | Not high vs low protein | Search |
| --- | --- | --- | --- |
|  | Allingstrup MJ, Kondrup J, Wiis J, et al. Early goal-directed nutrition versus standard of care in adult intensive care patients: the single-centre, randomised, outcome assessor-blinded EAT-ICU trial. *Intensive Care Med.* 2017;43(11):1637-1647. doi:10.1007/s00134-017-4880-3 | Significant different in energy intake | Previous SR |
|  | Arabi YM, Al-Dorzi HM, Mehta S, et al. Association of protein intake with the outcomes of critically ill patients: a post hoc analysis of the PermiT trial. *Am J Clin Nutr.* 2018;108(5):988-996. doi:10.1093/ajcn/nqy189 | Post-hoc analysis of RCT | Search |
|  | ﻿Arefian NM, Teymourian H, Radpay B. Effect of partial parenteral versus enteral nutritional therapy on serum indices in multiple trauma patients. Tanaffos. 2007;6(4):37-41. http://ovidsp.ovid.com/ovidweb.cgi?T=JS&PAGE=reference&D=cctr&NEWS=N&AN=CN-00708370. | Significant different in energy intake; No clinically important outcomes | Search |
|  | Bauer P, Charpentier C, Bouchet C, Nace L, Raffy F, Gaconnet N. Parenteral with enteral nutrition in the critically ill. *Intensive Care Med*. 2000;26(7):893-900. doi:10.1007/s001340051278 | Significant different in energy intake | CCN website |
|  | Berger MM, Pantet O, Jacquelin-Ravel N, et al. Supplemental parenteral nutrition improves immunity with unchanged carbohydrate and protein metabolism in critically ill patients: The SPN2 randomized tracer study. *Clin Nutr.* 2019;38(5):2408-2416. doi:10.1016/j.clnu.2018.10.023 | Significant different in energy intake | Search |
|  | Braunschweig CA, Sheean PM, Peterson SJ, et al. Intensive nutrition in acute lung injury: a clinical trial (INTACT). *JPEN J Parenter Enteral Nutr.* 2015;39(1):13-20. doi:10.1177/0148607114528541 | Significant different in energy intake | Previous SR |
|  | Braunschweig CL, Freels S, Sheean PM, et al. Role of timing and dose of energy received in patients with acute lung injury on mortality in the Intensive Nutrition in Acute Lung Injury Trial (INTACT): a post hoc analysis. *Am J Clin Nutr.* 2017;105(2):411-416. doi:10.3945/ajcn.116.140764 | Post-hoc analysis of RCT | Search |
|  | Brinson RR, Kolts BE. Diarrhea associated with severe hypoalbuminemia: a comparison of a peptide-based chemically defined diet and standard enteral alimentation. *Crit Care Med*. 1988;16(2):130-136. | Protein intake was not reported | Personal file |
|  | Casaer MP, Langouche L, Coudyzer W, et al. Impact of early parenteral nutrition on muscle and adipose tissue compartments during critical illness. *Crit Care Med*. 2013;41(10):2298-2309. doi:10.1097/CCM.0b013e31828cef02 | Significant different in energy intake | Previous SR |
|  | Casaer MP, Mesotten D, Hermans G, et al. Early versus late parenteral nutrition in critically ill adults. *N Engl J Med*. 2011;365(6):506-517. doi:10.1056/NEJMoa1102662 | Significant different in energy intake | Personal file |
|  | Clevenger FW, Gerding D, Steinle E, Rodriguez DJ, Osler TM. Effectiveness and tolerance to highly concentrated vs conventional TPN formulas. J Surg Res. 1993;55(2):228-232. doi:10.1006/jsre.1993.1134 | Significant different in energy intake | Search |
|  | Davies ML, Chapple LS, Chapman MJ, Moran JL, Peake SL. Protein delivery and clinical outcomes in the critically ill: a systematic review and meta-analysis. *Crit Care Resusc*. 2017;19(2):117-127. | Systematic review & meta-analysis – included studies reviewed | Search |
|  | Doig GS, Simpson F, Heighes PT, et al. Restricted versus continued standard caloric intake during the management of refeeding syndrome in critically ill adults: a randomised, parallel-group, multicentre, single-blind controlled trial. *Lancet Respir Med*. 2015;3(12):943-952. doi:10.1016/S2213-2600(15)00418-X | Significant different in energy intake | Previous SR |
|  | Doig GS, Simpson F, Sweetman EA, et al. Early parenteral nutrition in critically ill patients with short-term relative contraindications to early enteral nutrition: a randomized controlled trial. *JAMA*. 2013;309(20):2130-2138. doi:10.1001/jama.2013.5124 | Significant different in energy intake | Personal file |
|  | Eyer SD, Micon LT, Konstantinides FN, et al. Early enteral feeding does not attenuate metabolic response after blunt trauma. *J Trauma.* 1993;34(5):639-644. doi:10.1097/00005373-199305000-00005 | Significant different in energy intake | Previous SR |
|  | Fetterplace K, Gill BMT, Chapple LS, Presneill JJ, Macisaac C, Deane AM. Systematic Review With Meta-Analysis of Patient-Centered Outcomes, Comparing International Guideline – Recommended Enteral Protein Delivery With Usual Care. *JPEN J Parenter Enter Nutr*. 2020;44(4):610-620. doi:10.1002/jpen.1725 | Systematic review & meta-analysis – included studies reviewed | Search |
|  | Gillis C, Roque PS, Bläss J, et al. High dose amino acid administration achieves an anabolic response in type 2 diabetic patients that is independent of glycaemic control: A randomized clinical trial. *Clin Nutr*. 2018;37(4):1163-1171. doi:10.1016/j.clnu.2017.04.016 | Not critically ill | Search |
|  | Goeters C, Wenn A, Mertes N, et al. Parenteral L-alanyl-L-glutamine improves 6-month outcome in critically ill patients. *Crit Care Med.* 2002;30(9):2032-2037. doi:10.1097/00003246-200209000-00013 | Immunonutrition | Previous SR |
|  | Greig PD, Elwyn DH, Askanazi J, Kinney JM. Parenteral nutrition in septic patients: effect of increasing nitrogen intake. Am J Clin Nutr. 1987;46(6):1040-1047. doi:10.1093/ajcn/46.6.1040 | Not RCT | Previous SR |
|  | Grünert A, Diesch R, Kilian J, Dölp R. Untersuchungen zur parenteralen Applikation von Aminosäuren bei septischen patienten [Parenteral administration of amino acids to septic patients]. Anaesthesist. 1984;33(1):11-19. | Unable to find the full-text article | Search |
|  | Hausmann D, Mosebach KO, Caspari R, Rommelsheim K. Combined enteral-parenteral nutrition versus total parenteral nutrition in brain-injured patients. A comparative study. *Intensive Care Med*. 1985;11(2):80-84. doi:10.1007/BF00254779 | Significant different in energy intake | Search |
|  | Heidegger CP, Berger MM, Graf S, et al. Optimisation of energy provision with supplemental parenteral nutrition in critically ill patients: a randomised controlled clinical trial. *Lancet*. 2013;381(9864):385-393. doi:10.1016/S0140-6736(12)61351-8 | Significant different in energy intake | Search |
|  | Heimburger DC, Geels VJ, Bilbrey J, Redden DT, Keeney C. Effects of small-peptide and whole-protein enteral feedings on serum proteins and diarrhea in critically ill patients: a randomized trial. *JPEN J Parenter Enteral Nutr.* 1997;21(3):162-167. doi:10.1177/0148607197021003162 | No difference in energy and protein intake | Search |
|  | Heyland D, Muscedere J, Wischmeyer PE, et al. A randomized trial of glutamine and antioxidants in critically ill patients [published correction appears in N Engl J Med. 2013 May 9;368(19):1853. Dosage error in article text.]. *N Engl J Med.* 2013;368(16):1489-1497. doi:10.1056/NEJMoa1212722 | Immunonutrition | Previous SR |
|  | Hoffer LJ, Bistrian BR. Appropriate protein provision in critical illness: a systematic and narrative review. *Am J Clin Nutr*. 2012;96(3):591-600. doi:10.3945/ajcn.111.032078 | Systematic review & meta-analysis – included studies reviewed | Search |
|  | Hsieh LC, Chien SL, Huang MS, Tseng HF, Chang CK. Anti-inflammatory and anticatabolic effects of short-term beta-hydroxy-beta-methylbutyrate supplementation on chronic obstructive pulmonary disease patients in intensive care unit. Asia Pac J Clin Nutr. 2006;15(4):544-550. | Not high vs low protein | Previous SR |
|  | Hsu CW, Sun SF, Lin SL, et al. Duodenal versus gastric feeding in medical intensive care unit patients: a prospective, randomized, clinical study. *Crit Care Med.* 2009;37(6):1866-1872. doi:10.1097/CCM.0b013e31819ffcda | Significant different in energy intake | Previous SR |
|  | Huang HH, Chang SJ, Hsu CW, Chang TM, Kang SP, Liu MY. Severity of illness influences the efficacy of enteral feeding route on clinical outcomes in patients with critical illness. *J Acad Nutr Diet.* 2012;112(8):1138-1146. doi:10.1016/j.jand.2012.04.013 | Significant different in energy intake | Previous SR |
|  | Iapichino G, Radrizzani D, Scherini A, et al. Essential and non-essential amino acid requirement in injured patients receiving total parenteral nutrition. *Intensive Care Med*. 1988;14(4):399-405. doi:10.1007/BF00262896 | Not RCT | Previous SR |
|  | Ibrahim EH, Mehringer L, Prentice D, et al. Early versus late enteral feeding of mechanically ventilated patients: results of a clinical trial. JPEN J Parenter Enteral Nutr. 2002;26(3):174-181. doi:10.1177/0148607102026003174 | Significant different in energy intake | Previous SR |
|  | Ishibashi N, Plank LD, Sando K, Hill GL. Optimal protein requirements during the first 2 weeks after the onset of critical illness. Crit Care Med. 1998;26(9):1529-1535. doi:10.1097/00003246-199809000-00020 | Not RCT | Previous SR |
|  | Jensen GL, Miller RH, Talabiska DG, Fish J, Gianferante L. A double-blind, prospective, randomized study of glutamine-enriched compared with standard peptide-based feeding in critically ill patients. Am J Clin Nutr. 1996;64(4):615-621. doi:10.1093/ajcn/64.4.615 | Immunonutrition | Previous SR |
|  | Kagan I, Kremer S, Theilla M, Bendavid I, Singer P, Cohen J. OR63: Effect of Combined Protein Enriched Enteral Feeding and Early Cycle Ergometry in Mechanically Ventilated Critically Ill Patients: A Prospective, Randomized, Comparative, Single-Blind Controlled Study. *Clin Nutr.* 2017;36:S25-S26. doi:10.1016/S0261-5614(17)30724-0 | Abstract only | Search |
|  | Kearns PJ, Chin D, Mueller L, Wallace K, Jensen WA, Kirsch CM. The incidence of ventilator-associated pneumonia and success in nutrient delivery with gastric versus small intestinal feeding: a randomized clinical trial. *Crit Care Med*. 2000;28(6):1742-1746. doi:10.1097/00003246-200006000-00007 | Significant different in energy intake | Previous SR |
|  | Kerrie JP, Bagshaw SM, Brindley PG. Best evidence in critical care medicine. Early versus late parenteral nutrition in the adult ICU: feeding the patient or our conscience?. Can J Anaesth. 2012;59(5):494-498. doi:10.1007/s12630-012-9674-z | Commentary of EPANIC trial | Search |
|  | Kuhls DA, Rathmacher JA, Musngi MD, et al. Beta-hydroxy-beta-methylbutyrate supplementation in critically ill trauma patients. J Trauma. 2007;62(1):125-132. doi:10.1097/TA.0b013e31802dca93 | Not high vs low protein | Previous SR |
|  | Lambell KJ, King SJ, Forsyth AK, Tierney AC. Association of Energy and Protein Delivery on Skeletal Muscle Mass Changes in Critically Ill Adults: A Systematic Review. *JPEN* *J Parenter Enter Nutr*. 2018;42(7):1112-1122. doi:10.1002/jpen.1151 | Systematic review & meta-analysis – included studies reviewed | Search |
|  | Larsson J, Lennmarken C, Mårtensson J, Sandstedt S, Vinnars E. Nitrogen requirements in severely injured patients. *Br J Surg*. 1990;77(4):413-416. doi:10.1002/bjs.1800770418 | No clinically important outcome | Previous SR |
|  | Liebau F, Sundström M, van Loon LJ, Wernerman J, Rooyackers O. Short-term amino acid infusion improves protein balance in critically ill patients. *Critical Care.* 2015;19(1):106. doi:10.1186/s13054-015-0844-6. | Not RCT | CCN website |
|  | Long CL, Crosby F, Geiger JW, Kinney JM. Parenteral nutrition in the septic patient: nitrogen balance, limiting plasma amino acids, and calorie to nitrogen ratios. *Am J Clin Nutr.* 1976;29(4):380-391. doi:10.1093/ajcn/29.4.380 | Not RCT. No clinically important outcome | Previous SR |
|  | Ma N, Shen M, Wan Z, Pan S, Liu X, Yao Z. Zhonghua Wei Zhong Bing Ji Jiu Yi Xue. 2018;30(2):176-180. doi:10.3760/cma.j.issn.2095-4352.2018.02.016 | Significant different in energy intake; no different in protein | Search |
|  | Mansoor O, Breuillé D, Béchereau F, et al. Effect of an enteral diet supplemented with a specific blend of amino acid on plasma and muscle protein synthesis in ICU patients. *Clin Nutr.* 2007;26(1):30-40. doi:10.1016/j.clnu.2006.07.007 | Not high vs low protein | Previous SR |
|  | McKeever L, Peterson SJ, Lateef O, et al. Higher Caloric Exposure in Critically Ill Patients Transiently Accelerates Thyroid Hormone Activation. *J Clin Endocrinol Metab*. 2020;105(2):dgz077. doi:10.1210/clinem/dgz077 | Significant different in energy intake | Personal file |
|  | Meirelles CMJ, de Aguilar-Nascimento JE. Enteral or parenteral nutrition in traumatic brain injury: a prospective randomised trial. Nutr Hosp. 2011;26(5):1120-1124. doi:10.1590/S0212-16112011000500030 | Significant different in energy intake | Search |
|  | Meredith JW, Ditesheim JA, Zaloga GP. Visceral protein levels in trauma patients are greater with peptide diet than with intact protein diet. *J Trauma.* 1990;30(7):825-829. doi:10.1097/00005373-199007000-00011 | No difference in energy and protein intake | Personal file |
|  | Mesejo A, Montejo-González JC, Vaquerizo-Alonso C, et al. Diabetes-specific enteral nutrition formula in hyperglycemic, mechanically ventilated, critically ill patients: a prospective, open-label, blind-randomized, multicenter study. *Crit Care*. 2015;19:390. doi:10.1186/s13054-015-1108-1 | No difference in energy and protein intake | Search |
|  | Mowatt-Larssen CA, Brown RO, Wojtysiak SL, Kudsk KA. Comparison of tolerance and nutritional outcome between a peptide and a standard enteral formula in critically ill, hypoalbuminemic patients. *JPEN J Parenter Enteral Nutr.* 1992;16(1):20-24. doi:10.1177/014860719201600120 | No difference in energy and protein intake | Personal file |
|  | Nakamura K, Kihata A, Naraba H, et al. β-Hydroxy-β-methylbutyrate, Arginine, and Glutamine Complex on Muscle Volume Loss in Critically Ill Patients: A Randomized Control Trial. J*PEN J Parenter Enteral Nutr.* 2020;44(2):205-212. doi:10.1002/jpen.1607 | Immunonutrition | Personal file |
|  | National Heart, Lung, and Blood Institute Acute Respiratory Distress Syndrome (ARDS) Clinical Trials Network, Rice TW, Wheeler AP, et al. Initial trophic vs full enteral feeding in patients with acute lung injury: the EDEN randomized trial. *JAMA*. 2012;307(8):795-803. doi:10.1001/jama.2012.137 | Significant different in energy intake | Personal file |
|  | ﻿Ochoa J, Huhmann MB, Files DC, et al. Hypocaloric high-protein enteral nutrition improves glucose management in critically ill patients. *JPEN J Parenter Enter Nutr. 2017;41(2):289*. doi:http://dx.doi.org/10.1177/0148607116686023 (Abstract 30) | Abstract only; Significant different in energy intake | Search |
|  | Ott LG, Schmidt JJ, Young AB, et al. Comparison of administration of two standard intravenous amino acid formulas to severely brain-injured patients. Drug Intell Clin Pharm. 1988;22(10):763-768. doi:10.1177/106002808802201004 | No difference in energy and protein intake | Search |
|  | Ozgultekin A, Turan G, Durmus Y, et al. Comparison of the efficacy of parenteral glutamine and branched-chain amino acid solutions given as extra supplements in parallel to the enteral nutrition in head trauma. *E Spen Eur E J Clin Nutr Metab* 2008; 3: e211-6. | Immunonutrition | Previous SR |
|  | ﻿Pertikov SS, Solodov AA, Tveritnev PM, et al. Use A High-Protein Tube Feeding In Critically Ill Patients: The Results Of A Multicenter Study. *Clin Nutr.* 2019;38(Supplement 1):S300. doi:10.1016/S0261-5614%2819%2932614-7 | Abstract only | Search |
|  | Petros S, Horbach M, Seidel F, Weidhase L. Hypocaloric vs Normocaloric Nutrition in Critically Ill Patients: A Prospective Randomized Pilot Trial. *JPEN J Parenter Enteral Nutr.* 2016;40(2):242-249. doi:10.1177/0148607114528980 | Significant different in energy intake | Personal file |
|  | Pitkänen O, Takala J, Pöyhönen M, Kari A. Nitrogen and energy balance in septic and injured intensive care patients: response to parenteral nutrition. *Clin Nutr.* 1991;10(5):258-265. doi:10.1016/0261-5614(91)90004-v | No clinically important outcome | CCN websie |
|  | Qiu C, Chen C, Zhang W, et al. Fat-Modified Enteral Formula Improves Feeding Tolerance in Critically Ill Patients: A Multicenter, Single-Blind, Randomized Controlled Trial. *JPEN J Parenter Enteral Nutr.* 2017;41(5):785-795. doi:10.1177/0148607115601858 | Significant different in energy intake | Previous SR |
|  | ﻿Rice TW, Files DC, Morris P, et al. Facilitated glucose control in critically Ill patients utilizing a very high protein low carbohydrate formula. *Intensive Care Med Exp.* 2017;5(2):269. doi:10.1186/s40635-017-0151-4 | Abstract only; Similar protein between group | Search |
|  | Rice TW, Mogan S, Hays MA, Bernard GR, Jensen GL, Wheeler AP. Randomized trial of initial trophic versus full-energy enteral nutrition in mechanically ventilated patients with acute respiratory failure. *Crit Care Med.* 2011;39(5):967-974. doi:10.1097/CCM.0b013e31820a905a | Significant different in energy intake | Previous SR |
|  | Ridley EJ, Davies AR, Parke R, et al. Supplemental parenteral nutrition versus usual care in critically ill adults: a pilot randomized controlled study. *Crit Care*. 2018;22(1):12. doi:10.1186/s13054-018-1939-7 | Significant different in energy intake | Personal file |
|  | Saffle JR, Larson CM, Sullivan J. A randomized trial of indirect calorimetry-based feedings in thermal injury. *J Trauma*. 1990;30(7):776-783. doi:10.1097/00005373-199007000-00003 | No clinically important outcomes reported by protein group | Personal file |
|  | Scheinkestel CD, Kar L, Marshall K, Bailey M, Davies A, Nyulasi I, Tuxen DV. Prospective randomized trial to assess caloric and protein needs of critically Ill, anuric, ventilated patients requiring continuous renal replacement therapy. *Nutrition*. 2003 Nov-Dec;19(11-12):909-16. | Not high vs low protein | Search |
|  | Schmitz JE, Lotz P, Ahnefeld FW, Grünert A. Untersuchungen zur Eiweiss- und Energieversorgung von Intensivpatienten [Protein and energy metabolism in intensive care patients]. *Infusionsther Klin Ernahr*. 1981;8(4):158-162. | Unable to find the full-text article; No clinically important outcome | Search |
|  | ﻿Seres DS, Ippolito PR. Pilot study evaluating the efficacy, tolerance and safety of a peptide-based enteral formula versus a high protein enteral formula in multiple ICU settings (medical, surgical, cardiothoracic). Clin Nutr. 2017;36(3):706‐709. doi:10.1016/j.clnu.2016.04.016 | No clinically important outcome per group; Nutrition intake per group not reported | Search |
|  | Serra MC, Verceles AC, Parker EA, Feinberg T. Effects of protein supplementation on energy and protein intake: Preliminary findings from a randomized, controlled trial in the intensive care unit. *Glob Adv Heal Med.* 2018;7:118. doi:10.1177/2164956118773837 (Abstract 2251) | Abstract only: Significant different in energy intake; No clinically important outcomes | Search |
|  | Shaw JH, Wildbore M, Wolfe RR. Whole body protein kinetics in severely septic patients. The response to glucose infusion and total parenteral nutrition. *Ann Surg*. 1987;205(3):288-294. doi:10.1097/00000658-198703000-00012 | Not RCT | Previous SR |
|  | Singer P, Anbar R, Cohen J, et al. The tight calorie control study (TICACOS): a prospective, randomized, controlled pilot study of nutritional support in critically ill patients. *Intensive Care Med*. 2011;37(4):601-609. doi:10.1007/s00134-011-2146-z | Significant different in energy intake | Search |
|  | Singer P, De Waele E, Sanchez C, et al. TICACOS international: A multi-center, randomized, prospective controlled study comparing tight calorie control versus Liberal calorie administration study. *Clin Nutr*. 2021;40(2):380-387. doi:10.1016/j.clnu.2020.05.024 | Significant different in energy intake | Search |
|  | Taylor SJ, Fettes SB, Jewkes C, Nelson RJ. Prospective, randomized, controlled trial to determine the effect of early enhanced enteral nutrition on clinical outcome in mechanically ventilated patients suffering head injury. *Crit Care Med.* 1999;27(11):2525-2531. doi:10.1097/00003246-199911000-00033 | Significant different in energy intake | Search |
|  | Tiengou LE, Gloro R, Pouzoulet J, et al. Semi-elemental formula or polymeric formula: is there a better choice for enteral nutrition in acute pancreatitis? Randomized comparative study. *JPEN J Parenter Enteral Nutr.* 2006;30(1):1-5. doi:10.1177/014860710603000101 | No difference in energy and protein intake | Personal file |
|  | Twyman D, Young AB, Ott L, Norton JA, Bivins BA. High protein enteral feedings: a means of achieving positive nitrogen balance in head injured patients.. *JPEN J Parenter Enteral Nutr.* 1985 Nov-Dec;9(6):679-84. | No clinically important outcomes | Search |
|  | van der Heijden A, Verbeek MJ, Schreurs VV, Akkermans LM, Vos A. Efecto del aumento de la ingesta de proteínas sobre el balance nitrogenado en pacientes críticos ventilados mecánicamente y sometidos a nutrición parenteral total [Effect of increasing protein ingestion on the nitrogen balance of mechanically ventilated critically ill patients receiving total parenteral nutrition]. *Nutr Hosp.* 1993;8(5):279-287. | Abstract only; No clinically important outcomes | Search |
|  | Verbruggen SC, Coss-Bu J, Wu M, Schierbeek H, Joosten KF, Dhar A, et al. Current recommended parenteral protein intakes do not support protein synthesis in critically ill septic, insulin-resistant adolescents with tight glucose control. *Crit Care Med* 2011;39(11):2518-25. | Adolescent | CCN website |
|  | Wandrag L, Brett SJ, Frost G, Hickson M. Impact of supplementation with amino acids or their metabolites on muscle wasting in patients with critical illness or other muscle wasting illness: a systematic review. *J Hum Nutr Diet*. 2015;28(4):313-330. doi:10.1111/jhn.12238 | Systematic review & meta-analysis – included studies reviewed | Search |
|  | Wichansawakun S, Wongkongkathep P, & Tantiyavarong, P. Clin Nutr 2019.. MON-PO626: A Randomized Controlled Trial of the Effect of Protein Restriction to Delay Renal Replacement Therapy in Septic Patients with Acute Renal Failure in Thammasat University Hospital, Preliminary Analysis. doi:﻿10.1016/S0261-5614%2819%2932459-8 | Abstract only | Search |
|  | Wischmeyer PE, Hasselmann M, Kummerlen C, et al. A randomized trial of supplemental parenteral nutrition in underweight and overweight critically ill patients: the TOP-UP pilot trial. *Crit Care*. 2017;21(1):142. doi:10.1186/s13054-017-1736-8 | Significant different in energy intake | Search |
|  | Wolfe RR, Goodenough RD, Burke JF, Wolfe MH. Response of protein and urea kinetics in burn patients to different levels of protein intake. *Ann Surg.* 1983;197(2):163-171. doi:10.1097/00000658-198302000-00007 | No clinically important outcomes | Previous SR |

CCN: critical care nutrition, RCT: randomized controlled trial, SR: systematic review

**Table S3: Clinical Trial Registry of Ongoing Studies**

| **No** | **Trial Registration Number** | **Country** | **Title** | **Acronym** | **N** | **Intervention** | **Control** | **Primary outcome** | **Last Update** | **Status** |
| --- | --- | --- | --- | --- | --- | --- | --- | --- | --- | --- |
| 1 | CTRI/2020/02/ 023512 | India | Effect of High Protein Enteral Nutrition on Muscle Wasting in Critically Ill Patients | - | 30 | 1.5 g/kg/d | 1.0 g/kg/d | Skeletal muscle wasting during the first week of critical illness | February 24, 2020 | Not yet recruiting |
| 2 | IRCT  20180619040151N4 | Iran | Evaluation of the effect of high protein nutritional support in comparison to nutritional support with regular protein intake in intensive care unit patients: a double-blind randomized clinical trial | - | 60 | 2.2 g/kg/d whey protein | 1.2 g/kg/d whey protein | 60 days mortality | November 7, 2019 | Completed |
| 3 | NCT02106624 | China | A Trial to Assess the Effect of High Nitrogen Intake in Critically Ill Patients | - | 89 | Up to 2.5-3.0 g/kg lean mass weight (PN/EN) | Up to 1.2-1.5 g/kg lean mass weight (PN/EN) | All-cause mortality (28 & 90 days) | May 27, 2020 | Completed |
| 4 | NCT02837861 | USA | Early and Adequate Protein Feeding Post-Traumatic Injury | EMS | 45 | Routine nutritional support + Supplemental IV amino acids 1.5-2g/kg/day | Routine nutritional support | Nitrogen balance and Catabolic Index | October 27, 2020 | Completed |
| 5 | NCT02865408 | Canada | Amino Acid Nutrition in the Critically-ill | AA-ICU | 30 | 1) 2.5 g/kg/d IV amino acid and EN  2) 1.75 g/kg/d IV amino acid and EN | 1.0 g/kg/d EN | Whole body protein balance (0-48h) | February 24, 2021 | Recruiting |
| 6 | NCT03160547 | Canada | The Effect of Higher Protein Dosing in Critically Ill Patients | EFFORT | 4000 | ≥2.2 g/kg/d protein/amino-acid | ≤1.2 g/kg/d protein/amino-acid | 60-day mortality | December 16, 2020 | Recruiting |
| 7 | NCT03170401 | USA | Supplemental Enteral Protein in Critical Illness |  | 500 | EN with additional protein  supplementation | No protein supplementation | Serum concentrations of transthyretin at 3 weeks after injury. | February 19, 2018 | Recruiting |
| 8 | NCT03231540 | Netherlands | The PREServation of MUScle Function in Critically Ill Patients (PRESMUS) | PRESMUS | 50 | 1.5 g/kg/d through why protein supplement | 1g/kg/d | In vitro loss of skeletal muscle function | July 27, 2017 | Recruiting |
| 9 | NCT03480555 | Saudi Arabia | Replacing Protein Via Enteral Nutrition in a Stepwise Approach in Critically Ill Patients | Replenish | 40 | 2 g protein/kg/d (range 1.8-2.2 g/kg/d) | 0.8-1 g protein/kg/d | Recruitment rate and achievement of protein intake | January 5, 2021 | Completed |
| 10 | NCT04012333 | Germany/ Canada | The Effect of Higher Protein Dosing in Critically Ill Patients: A Multicenter Randomized Trial | EFFORT combo | 142 | >2.2g/kg/day by EN and PN | <1.2g/kg/day by EN only | 6-minute walking distance at hospital discharge | March 12, 2020 | Not yet recruiting |
| 11 | NCT04177446 | China | A Study About Nutritional Support of Enhanced Protein in Critical Patients | - | 180 | Extra protein | Extra maltodextrin as placebo | Urea nitrogen in 24h | December 20, 2019 | Not yet recruiting |
| 12 | NCT04468503 | Pakistan | Comparative Effect of Protein Prescription Strategies on Nitrogen Balance and Upshots in Critically Ill Patients | - | 100 | Target 2g/kg/d | Target 2g/kg/d | Change in nitrogen balance every 72h | July 13, 2020 | Not yet recruiting |
| 13 | NCT04475666 | Saudi Arabia | Replacing Protein Via Enteral Nutrition in Critically Ill Patients | REPLENISH | 2502 | Standard amount of protein (max 1.2 g/kg/d) + supplemental protein at 1.2 g/kg/d | Standard amount of protein (max 1.2 g/kg/d) only. | 90 day-all cause mortality | February 24, 2021 | Recruiting |
| 14 | NCT04633421 | Netherlands | PRotEin Provision in Critical IllneSs | PRECISe | 824 | EN feed with 8g protein/100 kcal (target 2.0 g/kg/d) | EN feed with 5g protein/100 kcal (target 1.2 g/kg/d) | Health Related Quality of Life (HRQL) | March 24, 2021 | Recruiting |
| 15 | UMIN000030866 | Japan | Efficacy and safety of high-protein early enteral nutrition in critically ill patients with respiratory failure, single-center prospective randomized control trial | - | 20 | 1.2-1.5 g/kg/d by EN | 0.6-1.2 g/kg by EN | N/A | July 20, 2020 | Completed |
| **Studies that combined higher protein and early mobility/exercise** | | | | | | | | | | |
| 16 | NCT02509520 | USA | Assessing The Effects of Exercise, Protein, and Electric Stimulation On Intensive Care Unit Patients Outcomes | ExPrEs | 60 | Mobility-based Physical Rehab + High protein | Mobility-based Physical Rehab only | Muscle mass strength and physical function | November 20, 2020 | Active, not recruiting |
| 17 | NCT03021902 | Canada/ USA | Nutrition and Exercise in Critical Illness | NEXIS | 142 | 2.0-2.5 g/kg/d IV amino acids + in-bed cycle ergometry | Usual care | 6 minute walk distance at hospital discharge | February 11, 2019 | Recruiting |
| 18 | NCT03469882 | Brazil | High Protein Intake and Early Exercise in Adult Intensive Care Patients | - | 120 | 2.0-2.5 g/kg/d + in-bed cycle ergometry exercise | Usual protein and exercise | Physical component summary (PCS) 3 and 6 months after randomization | May 6, 2019 | Recruiting |
| 19 | NCT04099108 | South Africa | Effect of Combined IV Bolus Amino Acid Supplementation and Mobilisation on Muscle Mass in Patients Over the First Week of ICU Care: RCT | - | 80 | Combined cycle ergometry and bolus amino acid supplementation | Standard care only | Change in myofiber cross-sectional area over first week of ICU(muscle biopsy and ultrasound) | September 30, 2019 | Not yet recruiting |
| 20 | NCT04261543 | Malaysia | The Effect of High Protein and Early Resistance Exercise Versus Usual Care in Critically Ill Patients (EFFORT-X Trial) | EFFORT-X | 120 | ≥2.2 g/kg boy weight through EN + Early cycle ergometry for 45 minutes/day | ≤1.2 g/kg body weight + usual care | Rectus femoris cross-sectional area and linear depth mass at Day10 of randomization (Ultrasound) | February 7, 2020 | Recruiting |
| 21 | NTR7010 | Netherlands | Effect of early mobilisation combined with additional protein on muscle mass in critically ill patients | TRAIN-ICU | 40 | 1.5 g/kg/d + early mobilization 3 time/d (add 15g protein after mobilization) | 1.5 g/kg/d + early mobilization 3 times/d | Muscle thickness of the mid-upper arm, forearm and thigh measured by ultrasonography | February 2, 2018 | NA |

Note: included registries from both ClinicalTrial.gov and the database searching

**Table S4: Patients’ Baseline Characteristics of Included Studies**

| **Author, year (country)** | **Age** | | **Sex (M/F)** | | **APACHE II** | | **SOFA** | | **MV,%** | | **Medical,%** | | **Sepsis.%** | | **Weight, kg or BMI, kg/m^2^** | |
| --- | --- | --- | --- | --- | --- | --- | --- | --- | --- | --- | --- | --- | --- | --- | --- | --- |
|  | **High** | **Low** | **High** | **Low** | **High** | **Low** | **High** | **Low** | **High** | **Low** | **High** | **Low** | **High** | **Low** | **High** | **Low** |
| Clifton 1985^1^  (USA) | 34±12 | 38±12 | 9/1 | 10/0 | - | - | - | - | - | - | - | - | - | - | - | - |
| Mesejo 2003^2^  (Spain) | 64.6±9.63 | 65.2 ±14.95 | 17/7 | 24/2 | 18 (14-21) | 19 (16-20) | - | - | - | - | - | - | - | - |  |  |
| Zhou 2006^3^  (China) | 65.7±15.6 | | 29/22 | | APACHE II>19: 31/51 (60.8) | | - | - | - | - | - | - | - | - | - | - |
| Singer 2007^4^  (Israel) | 74±6 | 55±20 | - | - | SAPS II: 13.6±3.4 | SAPS II: 12.8±3.3 | - | - | 100 | 100 | - | - | 12.5 | 16.7 | - | - |
| Rugeles 2013^5^ (Columbia) | 53.3 ±19.5 | 55.7 ±19.5 | 22/18 | 24/16 | 13.9±4.8 | 15.1 ±6.2 | 7.5±2.9 | 6.7±2.5 | - | - | 100 | 100 | - | - | 63±10.7 | 65.8±11.0 |
| Doig 2015^6^ (Australia) | 63.3 ±15.4 | 62.7 ±16.6 | 158/81 | 147/88 | 21.7 ±7.6 | 20.2 ±6.8 | - | - | 82.4 | 82.5 | 66.5 | 66.4 | 14.6 | 10.6 | BMI:  28.9 ± 7.0 | BMI:  29.5 ± 6.9 |
| Ferrie 2015^7^  (Australia) | 67.0 (55.5-74.3) | 64.5 (49.3-70.0) | 38/22 | 36/24 | 25.5 ±9.4 | 23.7 ±8.1 | 9.4 ±4.1 | 9.2 ±4.0 | 95 | 98 | - | - | - | - | 73.2 ±16.1 | 77.7 ±21.7 |
| Jakob 2017^8^  (Switzerland) | 65.3 (52.6-75.3) | 61.6 (48.6-71.3) | 33/13 | 28/16 | 28.5 (22.3-32.8) | 27.5 (22.0-33.3) | 8.0 (6.0-11.0) | 7.0 (5.0-10.0) | 94 | 98 | - | - | - | - | BMI: 28.8 (25.1-34.2) | BMI: 27.8 (23.5-31.5) |
| Fetterplace 2018^9^  (Australia) | 55±13 | 57±16 | 23/7 | 21/9 | 22±6.2 | 20 ±5.9 | - | - | 100 | 100 | 70 | 53 | 10 | 3.3 | BMI: 30 ±7.1 | BMI: 29 ±5.3 |
| Van Zanten 2018^10^ (Netherlands) | 63.9 ±13.3 | 60.8 ±15.2 | 9/13 | 13/9 | 25 (21-28) | 24 (18-27) | 10 (9-11) | 9 (7-11) | 100 | 100 | 36.4 | 40.9 | - | - | 84.9 ±18.3  BMI: 30.3 ±4.1 | 91.2 ±20.7  BMI: 30.7 ±8.4 |
| Vega-Alava 2018^11^ (Philippines) | 57 (47-70) | 59 (45-72) | 11/9 | 9/11 | SAPS II: 23.5 (19-34) | SAPS II: 25.7 (19-36) | - | - | 100 | 100 | - | - | - | - | BMI: 22.23 (19.6-25.7) | BMI: 23.21 (18.8-26.1) |
| Azevedo 2019^12^ (Brazil) | 65.0 ±18.8 | 67.4 ±18.9 | 34/23 | 32/31 | APACHE IV: 81.1 ±32.4 | APACHE IV: 77.2 ±30.7 | 9.8 ±14.6 | 6.8 ±4.0 | 100 | 100 | 80 | 73 | 21.0 | 23.8 | - | - |
| Danielis 2019^13^  (Italy) | 66 (57-72) | 63 (46-70) | 11/8 | 11/10 | 17 (13.5-22) | 17 (13-22) | - | - | 100 | 100 | - | - | - | - | BMI: 25 (23-27.5) | BMI: 24 (21-26) |
| Badjatia 2020^14^ (USA) | 60±8 | 58±14 | 5/7 | 5/8 | 20±5 | 18±10 | - | - | - | - | - | - | - | - | - | - |
| Bukhari 2020^^15^  (Indonesia) | TBI: 38.29 ±18.35  Non-TBI: 50.25 ±15.92 | TBI: 41.6 ±20.11  Non-TBI: 42.44 ±20.27 | - | - | - | - | - | - | - | - | - | - | - | - | IBW  TBI: 59.27 ±6.87  Non-TBI: 52.48 ±5.44 | IBW  TBI: 56.20 ±3.90  Non-TBI: 55.26 ±6.26 |
| Chapple 2020^16^ (Australia) | 60 (50-72) | 61 (46-68) | 39/19 | 44/14 | 22 (16-26) | 22 (16-27) | - | - | 100 | 100 | 81 | 71 | - | - | ABW: 85 (75-100)  IBW: 65 (57-71) | ABW: 85 (75-101)  IBW: 67 (57-72) |
| Nakamura 2020^17^ (Japan) | 68.3 ±14.3 | 67.9 ±14.9 | 35/25 | 38/19 | 18.6 ±8.1 | 18.2 ±6.0 | 6.8±3.1 | 7.1±3.5 | 85.0 | 91.2 | - | - | 45.0 | 52.6 | 53.1 ±9.6  BMI: 21.3 ±3.9 | 53.6 ±11.2  BMI: 21.5 ±4.5 |
| Carteron 2021^18^ (France) | 57 (44-65) | 55 (40-65) | 67/33 | 53/42 | SAPS II: 48±12 | SAPS II: 49±13 | - | - | 100 | 100 | - | - | - | - | IBW: 65 (58-70)  BMI: 26 (23-29) | IBW:  64 (56-70)  BMI: 26 (23-29) |
| Dresen 2021^19^ (Germany) | 66±16 | 64±15 | 15/6 | 15/6 | SAPS II: 46 ±12 | SAPS II: 45 ±10 | 6±3 | 8±3 | 100 | 100 | 0 | 0 | 90 | 81 | IBW @ BMI 25: 75 ±8.2 | IBW @ BMI 25: 78 ±9.6 |

Data are presented as Mean±SD or Median (Q1-Q3)

^This study has 3 groups: control (n=22), high-protein polymeric (n=19) and oligomeric group (n=14), the control group was excluded from the analysis.

M: male, F: female, APACHE II: acute physiology and chronic health evaluation II, SOFA: sequential organ failure assessment, MV: mechanical ventilation, BMI: body mass index, ABW: actual body weight, IBW: ideal body weight, SAPS II: simplified acute physiology score II, TBI: traumatic brain injury

The mean or median reported age ranged 34 to 74 years and 38 to 67.9 years in the higher and lower protein groups, respectively. The number of males and females in the higher and lower protein group was 526 and 292 and 510 and 305, respectively (excluding 3 studies that did not report sex per group^3,4,15^). Of the 10 studies that reported mean or median APACHE II score, it ranged 13.9 to 28.5 and 15.1 to 27.5 in the higher vs the lower protein groups, respectively. Four studies reported SAPS II score and it ranged 13.6 to 48 in the higher protein group and 15.1 to 49 in the lower protein group.^4,11,18,19^ Of the 10 studies that reported SOFA, it ranges from 6 to 10 and 6.7 to 9.2 in the higher and lower protein groups, respectively. Nine studies included only mechanically ventilated patients, while 4 studies included >80% of mechanically ventilated patients.^6–8,17^ Of the 7 studies that reported body mass index (BMI), patients in 6 of the studies were overweight or obese (BMI ≥25).^6,8–10,13,18^

**Table S5: Nutritional Prescription, Prescription body weight and Energy and Protein Delivered**

| **Author, year (country)** | **Nutritional Prescription** | | **Prescription body weight** | | **Energy Delivered** | | **Protein Delivered** | |
| --- | --- | --- | --- | --- | --- | --- | --- | --- |
|  | **High** | **Low** | **High** | **Low** | **High** | **Low** | **High** | **Low** |
| Clifton 1985  (USA) | **E**: Repeated IC and provide 150% of resting metabolic expenditure  **P**: EN formula with 83g protein/liter; 22% calories from protein | **E**: Repeated IC and provide 150% of resting metabolic expenditure  **P**: EN formula with 70g protein/liter; 14% calories from protein | - | | 52±11 kcal/kg/d  141±15% of resting metabolic expenditure | 48±8 kcal/kg/d  139±9% of resting metabolic expenditure | 29.0±5.3 g N_2_/d (~181.3±33.1 g/d)  0.42±0.09 g N_2_/kg/d (~2.63±0.56 g/kg) | 17.6±3.6 g N_2_/d (~110±22.5 g/d)  0.24±0.04 g N_2_/kg/d  (~1.50±0.25 g/kg) |
| Mesejo 2003  (Spain) | **E**: Harris-benedict equation*1.2  **P:** EN Formula with 22% of calories from protein | **E**: Harris-benedict equation*1.2  **P:** EN Formula with 20% of calories from protein | IBW (method of calculation not reported) | | 1664±203 kcal/d | 1599±226 kcal/d | 14±2.48 N_2_/d (88.8±15.5 g/d) | 12.8±1.8 N_2_/d (80.0±11.3 g/d) |
| Zhou 2006  (China) | **E:** 25 kcal/kg  **P:** EN formula with NPC:N = 110:1 | **E:** 25 kcal/kg  **P:** EN formula with NPC:N = 130:1 | Ideal weight, kg = height in cm - 105 | | - | - | - | - |
| Singer 2007  (Sepsis) | **E:** 2000 kcal/day non-protein calories (dextrose and Intralipid)  **P:** 150 g amino acids | **E:** 2000 kcal/day non-protein calories (dextrose and Intralipid)  **P:** 75 g amino acids | - | | - | - | - | - |
| Rugeles 2013 (Columbia) | **E**: 15 kcal/kg/d  **P**: >1.5 g/kg/d | **E**: 25 kcal/kg/d  **P**: Usual care | - | | 12±0.5 kcal/kg/d^£^ | 14±0.5 kcal/kg/d^£^ | 1.4±0.1 g/kg/d^£^ | 0.76±0.2 g/kg/d^£^ |
| Doig 2015 (Australia) | **E**: -  **P**: up to 2.0 g/kg/d | Decide by the attending clinicians | If BMI>25, use IBW at BM 23 | Decide by the attending clinicians | eFig1c  ~1200 kcal/d | eFig1c  ~956 kcal/d | eFig1a ~1.67 g/kg/d | eFig1a ~0.71 g/kg/d |
| Ferrie 2015  (Australia) | **E**: 25 kcal/kg/d  **P**: PN formula with 57g amino acids/liter; 21.5% calories from amino acids | **E**: 25 kcal/kg/d  **P**: PN formula with 40g amino acids/liter; 13.3% calories from amino acids | Current weight if BMI 20-30; Weight at BMI 20 if underweight; Weight at BMI 27.5 if obese | | First 3 study days:  1053±450 kcal/d  23.5±3.9 kcal/kg/d  First 7 study days:  1610±468 kcal/d  23.1±3.9 kcal/kg/d | First 3 study days:  1700±524 kcal/d  26.0±3.8 kcal/kg/d  First 7 study days:  1720±516 kcal/d  24.9±4.2 kcal/kg/d | First 3 study days:  76±25 g/d  1.17±0.21 g/kg/d  First 7 study days:  76±26 g/d  1.09± 0.22 g/kg/d | First 3 study days:  55±20 g/d  0.87±1.17g/kg/d  First 7 study days:  60±21 g/d  0.90± 0.21 g/kg/d |
| Jakob 2017  (Switzerland) | **E**: 25 kcal/kg/d and adjusted by IC during the first night after study start, 3 days afterwards, at end of nutrition with the study product, and 2 days afterwards if still intubated  **P**: EN formula with 94 g protein/liter; 25% calories from protein | **E**: 25 kcal/kg/d and adjusted by IC during the first night after study start, 3 days afterwards, at end of nutrition with the study product, and 2 days afterwards if still intubated  **P**: EN formula with 61 g protein/liter; 16% calories from protein | Weight taken from medical records or relatives, or estimated by medical staff. (not clear ABW or IBW) | | 18 (12.5-20.9) kcal/kg  14.4±6.6 kcal/kg^£^  85 (71-95) % | 19.7 (17.3-23.1 kcal/kg)  17.2±6.4 kcal/kg^£^  90 (84-96)% | 1.13 (0.78-1.31) g/kg  0.9±0.4 g/kg^£^ | 0.80 (0.70-0.94) g/kg  0.7±0.2 g/kg^£^ |
| Fetterplace 2018  (Australia) | **E**: 25 kcal/kg/d  **P**: 1.5 g/kg/d  (Volume-based feeding protocol) | **E**: 25 kcal/kg/d  **P**: 1.0 g/kg/d | Age<65:  - use ABW if BMI 18.5-25  Age≥65:  - use ABW if BMI 22-27  If BMI≥32, use adjusted weight = ideal weight + 25% (actual weight – ideal weight) | | 1646±447 kcal/d  21±5.2 kcal/kg/d  Include non-nutrition E  1835±340 kcal/d  23±5.7 kcal/kg/d  84±21% | 1398±308 kcal/d  18±2.7 kcal/kg/d  Include non-nutrition E  1598±340 kcal/d  21±3.3 kcal/kg/d  73±11% | 94±27 g/d  1.2±0.3 g/kg/d  90±25% | 58±12 g/d  0.75±0.11g/kg/d  57±8% |
| van Zanten 2018 (Netherlands) | **E**: 25 kcal/kg IBW/d  **P**: EN formula with 8 g protein/100 kcal; 32% of calories from protein | **E**: 25 kcal/kg IBW/d  **P**: EN formula with 5 g protein/100 kcal; 20% of calories from protein | IBW at BMI 30 (if BMI>30). | | 1162±606 kcal/d (first 10d)  15.0±9.3 kcal/kg/d (first 10d)^£^  16.6 (8.9-23.3) kcal/kg IBW/d (28 days) | 1163±375 kcal/d (first 10d)  13.8±8.6 kcal/kg/d (first 10d)^£^  14.4 (10.9-18.8) kcal/kg IBW/d (28 days) | 1.37±0.82 g/kg IBW/d (day 5)  1.20±0.74 g/kg IBW/d (first 10d) ^£^ | 0.72±0.47 g/kg IBW/d (day 5)  0.70±0.43 g/kg IBW/d (first 10d) ^£^ |
| Vega-Alava 2018 (Philippines) | **E:** 25-30 kcal/kg IBW/d * stress factor  **P:** EN formula with hydrolyzed whey protein (10 g protein per serving and 16% of calories from protein) + 100% whey protein supplement (6 g protein per serving), 3 servings, every 8 hour | **E:** 25-30 kcal/kg IBW/d * stress factor  **P:** EN formula with hydrolyzed whey protein (10 g protein per serving and 16% of calories from protein) only | IBW computed based on height | | - | - | - | - |
| Azevedo 2019 (Brazil) | **E**: IC daily for the first 3 days, then IC every 2 days until day 10  **P**: 2.0-2.2 g/kg/d | **E**: 25 kcal/kg/d  **P**: 1.4-1.5 g/kg/d | - | | 1139 (890-1278) kcal/d  73.2% of IC | 1140 (889-1331) kcal/d  78% of 25 kcal/kg/d | 1.69 (1.33-1.80) g/kg/d  80% of 2.1 g/kg/d | 1.13 (0.97-1.34) g/kg/d  77.9% of 1.45 g/kg/d |
| Danielis 2019  (Italy) | **E:** *Age<60:* 8 x weight (kg) + 14 x height (cm) + 32 x minute ventilation (L/min) + 94 x temperature (degrees °C) – 4834; *Age >60:* (0.85 x Harris Benedict equation) + (175 x temperature degrees °C) + (33 x minute ventilation (L/min)) – 6433.  **P:** 1.8 g/kg/d  (*EN:* Protein 21%, Carbs/Dextrose 31%, Fats 46% Fiber 2%; *PN:* Protein 22%, Carbs/Dextrose 40%, Fats 38%) | **E:** 20-25 kcal/kg/d  **P:** follow energy and type of formula  (*EN:* Protein 16%, Carbs/Dextrose 35%, Fats 49%; *PN:* Protein 16%, Carbs/Dextrose 49%, Fats 35%) | Weight assessed by ICU bed scale | | 1490±292.8 kcal/d^£^ | 1460±403.3 kcal/d^£^ | 100±17.6 g/d^£^ | 52±18.1 g/d^£^ |
| Badjatia 2020 (USA) | **E**: Mifflin St-Jeor (non-intubated); Penn State (intubated)  **P**: 1.75 g/kg/d (≥9g leucine/d) | **E**: Mifflin St-Jeor (non-intubated); Penn State (intubated)  **P**: 1.2-1.4 g/kg/d | - | | 20.0±7.1 kcal/kg/d | 19.8±9.9 kcal/kg/d | 1.51±0.47 g/kg/d | 0.88±0.36 g/kg/d |
| Bukhari 2020^  (Indonesia) | **E**: 25-30kcal/kg/d  **P**: 1.2-2.0 g/kg/d  **EN Formula:** High-protein polymeric (22.4% calories from protein) | **E**: 25-30kcal/kg/d  **P**: 1.2-2.0 g/kg/d  **EN Formula:** Oligomeric (16.2% calories from protein) | IBW (method of calculation not reported) | | TBI: 2358.65±714.57 kcal/d  Non TBI: 1998.96 ±684.16 kcal/d  Pooled: 2131.48 ±695.04 kcal/d | TBI: 2336.94 ±891.93 kcal/d  Non TBI: 1887.12 ±598.72 kcal/d  Pooled: 2015.64 ±683.91 kcal/d | TBI: 134.03 ±42.58 g/d  Non-TBI: 102.88 ±37.47 g/d  Pooled: 114.36±39.35 g/d | TBI: 93.79±26.65 g/d  Non-TBI: 80.29±29.38 g/d  Pooled: 84.15±28.72 g/d |
| Chapple 2020 (Australia) | **Goal Volume:** 1mg/kg IBW/h (max 100 ml/h)  **E:** EN formula with 1260 kcal/liter  **P**: EN formula with 100 g protein/liter; 32% of calories from protein | **Goal Volume:** 1mg/kg IBW/h (max 100 ml/h)  **E:** EN formula with 1250 kcal/liter  **P**: EN formula with 63 g protein/liter; 20% of calories from protein | IBW - Men = (height (cm) – 152.4) * 0.9 + 50  - Women = (height (cm) – 152.4) * 0.9 + 45.5 | | Trial EN:  19.2±6.5 kcal/kg IBW/d  1233±487 kcal/d  Trial EN + PN:  1245±489 kcal/d | Trial EN:  19.6±5.4 kcal/kg IBW/d  1260±393 kcal/d  Trial EN + PN:  1271±392 kcal/d | Trial EN+PN, albumin, protein supplements:  1.52±0.52 g/kg IBW/d | Trial EN+PN, albumin, protein supplements:  0.99±0.27 g/kg IBW/d |
| Nakamura 2020 (Japan) | **E**: 20 kcal/kg/d (initial target), up to 30 kcal/kg/d if malnutrition (defined as BMI<18.5, recent weight loss>10%, or physician’s decision)  **P**: 1.8 g/kg/d | **E**: 20 kcal/kg/d (initial target), up to 30 kcal/kg/d if malnutrition (defined as BMI<18.5, recent weight loss>10%, or physician’s decision)  **P**: 0.9 g/kg/d | - | | 17.9±10.1 kcal/kg/d^£^ | 16.2±9.5 kcal/kg/d^£^ | 1.36±0.80 g/kg/d^£^ | 0.72±0.46 g/kg/d^£^ |
| Carteron 2021 (France) | **Goal Volume:** 42 ml/h for males and females with an estimated ideal body weight of ≤60kg; 63 ml/h for others.  **E**: EN formula with caloric density 1.5 kcal/ml  **P**: EN formula with 9.4 g of hydrolyzed protein per 100ml | **Goal Volume:** 42 ml/h for males and females with an estimated ideal body weight of ≤60kg; 63 ml/h for others.  **E**: EN formula with caloric density 1.5 kcal/ml  **P**: EN formula with 7.5 g of protein per 100ml | IBW by using the Lorentz’s Formula | | 20.2±6.3 kcal/kg/d | 21.0±6.5 kcal/kg/d | 1.3±0.4 g/kg/d | 1.1±0.3 g/kg/d |
| Dresen 2021 (Germany)* | **E**: Repeated IC or Harris-Benedict. Multiply with an illness-specific coefficient  **P**: 1.8 g/kg/d | **E**: Repeated IC or Harris-Benedict. Multiply with an illness-specific coefficient  **P**: 1.2 g/kg/d | IBW at BMI 25 | | 1989.3±655.2 kcal/d  27±8.9 kcal/kg/d  97±34% | 1951±828 kcal/d  24.6±9.8 kcal/kg/d  87±30% | 112.4±35 g/d  1.5±0.5 g/kg/d  86±26% | 81.8±31.9 g/d  1.0±0.4 g/kg/d  86±32% |

Data are presented as Mean±SD or Median (Q1-Q3).

^£^Information from the author

*^This study has 3 groups: control (n=22), high-protein polymeric (n=19) and oligomeric group (n=14), the control group was excluded from the analysis.*

E: energy, P: protein, EN: enteral nutrition, IC: Indirect calorimetry, N_2_: nitrogen intake, BMI: body mass index, ABW: actual body weight, IBW: ideal body weight, TBI: traumatic brain injury

Nitrogen intake (gram) / 0.16 = protein intake (gram)

**Table S6: Scores of the Methodological Quality Scoring System for all Included Studies**

| **Author, year (country)** | **Concealed**  **Randomization** | **Intention-to-treat**  **Analysis** | **Blinding** | **Patient**  **Selection** | **Comparability**  **of groups at**  **baseline** | **Extent of**  **Follow-up** | **Description**  **of treatment**  **protocol** | **Description**  **of treatment**  **co-interventions** | **Objectivity**  **of the definition**  **of outcomes** | **Total score**  **(max 14)** |
| --- | --- | --- | --- | --- | --- | --- | --- | --- | --- | --- |
| Clifton 1985  (USA) | 1 | 2 | 0 | 0 | 1 | 1 | 1 | 1 | 2 | **9** |
| Mesejo 2003  (Spain) | 2 | 2 | 0 | 0 | 0 | 1 | 1 | 0 | 1 | **7** |
| Zhou 2006  (China) | 1 | 2 | 0 | 0 | 1 | 1 | 0 | 0 | 2 | **7** |
| Singer 2007  (Israel) | 1 | 2 | 0 | 0 | 0 | 1 | 0 | 0 | 1 | **5** |
| Rugeles 2013  (Columbia) | 2 | 0 | 2 | 0 | 1 | 0 | 0 | 0 | 2 | **7** |
| Doig 2015  (Australia) | 2 | 0 | 0 | 1 | 0 | 0 | 1 | 1 | 2 | **7** |
| Ferrie 2015  (Australia) | 2 | 2 | 2 | 1 | 1 | 1 | 1 | 0 | 2 | **12** |
| Jakob 2017  (Switzerland) | 1 | 2 | 2 | 1 | 1 | 1 | 1 | 0 | 2 | **11** |
| Fetterplace 2018  (Australia) | 2 | 2 | 1 | 1 | 1 | 0 | 1 | 0 | 2 | **10** |
| Van Zanten 2018  (Netherlands) | 2 | 2 | 2 | 1 | 1 | 1 | 1 | 0 | 2 | **12** |
| Vega-Alava 2018  (Philippines) | 2 | 2 | 0 | 1 | 1 | 1 | 1 | 1 | 1 | **10** |
| Azevedo 2019  (Brazil) | 1 | 0 | 0 | 0 | 0 | 1 | 1 | 0 | 2 | **5** |
| Danielis 2019  (Italy) | 2 | 2 | 0 | 0 | 0 | 1 | 1 | 0 | 1 | **7** |
| Badjatia 2020  (USA) | 1 | 2 | 1 | 1 | 1 | 1 | 1 | 0 | 1 | **9** |
| Bukhari 2020  (Indonesia) | 1 | 0 | 0 | 1 | 0 | 0 | 1 | 0 | 2 | **5** |
| Chapple 2020  (Australia) | 2 | 0 | 2 | 1 | 1 | 1 | 1 | 0 | 2 | **10** |
| Nakamura 2020  (Japan) | 2 | 0 | 1 | 1 | 0 | 1 | 1 | 0 | 1 | **7** |
| Carteron 2021  (France) | 2 | 0 | 0 | 1 | 1 | 1 | 1 | 1 | 1 | **8** |
| Dresen 2021  (Germany) | 2 | 0 | 1 | 1 | 0 | 0 | 1 | 1 | 2 | **8** |

**Table S7: Outcomes summary**

| **Study** | **Mortality n(%)** | | **Infections n(%)** | | **Duration of Ventilation and Length of Stays (n)** | | **Muscle Mass**  **and Strength (n)** | | **Functional, Quality of Life outcomes and Discharge location (n)** | |
| --- | --- | --- | --- | --- | --- | --- | --- | --- | --- | --- |
|  | **Higher Protein** | **Lower Protein** | **Higher Protein** | **Lower Protein** | **Higher Protein** | **Lower Protein** | **Higher Protein** | **Lower Protein** | **Higher Protein** | **Lower Protein** |
| **1) Clifton 1985** | **3-mo**  1/10 (10) | **3-mo**  1/10 (10) | **Not specified**  3/10 (30) | **Not specified**  2/10 (20) | **NR** | **NR** | **NR** | **NR** | **NR** | **NR** |
| **2) Mesejo 2003** | **ICU**  7/24 (29.2) | **ICU**  8/26 (30.8) | **Hospital -acquired infection**  8/24 (33.3) | **Hospital-acquired infection**  10/26 (38.5) | **MV**  9.4±5.96 (24)  **ICU**  14.8±8.76 (24) | **MV**  8.7±6.18 (26)  **ICU**  14.8±9.39 (26) | **NR** | **NR** | **NR** | **NR** |
| **3) Zhou 2006** | **28-d**  7/25 (28)  **90-d**  11/25 (44) | **28-d**  10/26 (38.5)  **90-d**  11/26 (42.3) | **NR** | **NR** | **NR** | **NR** | **NR** | **NR** | **NR** | **NR** |
| **4) Singer 2007** | **ICU**  3/8 (37.5) | **ICU**  2/6 (33.3) | **NR** | **NR** | **NR** | **NR** | **NR** | **NR** | **NR** | **NR** |
| **5) Rugeles 2013** | **NR** | **NR** | **NR** | **NR** | **MV**  8.5±4.6 (n=40)  **ICU**  9.5±5.5 (n=40) | **MV**  9.7±4.9 (n=40)  **ICU**  10.4±5.0 (n=40) | **NR** | **NR** | **NR** | **NR** |
| **6) Doig 2015** | **ICU**  28/239 (11.7)  **Hospital**  37/239 (15.5)  **90-d**  42/236 (17.8) | **ICU**  30/235 (12.8)  **Hospital**  43/235 (18.3)  **90-d**  47/235 (20.0) | **NR** | **NR** | **MV**  7.33 (7.00-7.68) (239)  **ICU**  11.6 (10.8 to 12.5) (239)  **Hospital**  26.0 (24.2 to 28.0) (239) | **MV**  7.26 (6.94-7.61) (235)  **ICU**  10.7 (10.0 to 11.5) (235)  **Hospital**  24.8 (23.0 to 26.6) (235) | **NR** | **NR** | **D90 RAND-36 General Health**  50.5±27.2 (192)  **D90 ECOG Performance Status**  1.31±1.0 (192)  **D90 RAND-36 Physical Function**  47.7±33.7 (192) | **D90 RAND-36 General Health**  52.8±25.9 (180)  **D90 ECOG Performance Status**  1.18±1.0 (180)  **D90 RAND-36 Physical Function**  53.2±33.0 (180) |
| **7) Ferrie 2015** | **ICU**  8/59 (14)  **Hospital**  12/59 (20)  **6-mo**  15/59 (25) | **ICU**  6/60 (10)  **Hospital**  9/60 (15)  **6-mo**  9/60 (15**)** | **NR** | **NR** | **MV**  2 (1-3)  4.87±14.37^£^(59)  **ICU**  5 (3-8)  9.85±14.83^£^(59)  **Hospital**  25.0 (16.8-41.3)  41.75±37.36^£^(59) | **MV**  2 (1-5)  2.67±6.16^£^(59)  **ICU**  6 (3.8-10.0)  9.85±14.83^£^(60)  **Hospital**  27.5 (18.8-55.8)  37.70±35.88^£^(60) | **D7 forearm thickness, cm**  3.2±0.4 (35)^£^  **D7 biceps thickness, cm**  2.5±0.6 (35) ^£^  **D7 thigh area, cm^2^**  6.8±2.1(n=29) ^£^  **D7 ∑3 muscle, cm**  8.4±1.0 (39) ^£^  **D7 change in thigh area, %**  -8.4±31.1 (28)^£^  **D7 HG Str** 22.1±10.1 (52) | **D7 forearm thickness, cm**  2.8±0.4 (35) ^£^  **D7 biceps thickness, cm**  2.4±0.4 (35) ^£^  **D7 thigh area, cm^2^**  5.8±1.9 (28) ^£^  **D7 ∑3 muscle, cm**  7.9±1.1 (33) ^£^  **D7 change in thigh area, %**  30±139.2 (29)^£^  **D7 HG Str**  18.5±11.8 (56) | **NR** | **NR** |
| **8) Jakob 2017** | **NR** | **NR** | **Secondary infection**  19/46 (41.3) | **Secondary infection**  19/44 (43.2) | **MV**  6.2 (4.8-7.7)  2.8±2.6^£(^46)  **ICU**  7.0 (5.3-8.7)  9.0±7.7^£^(46)  **Hospital**  31.0 (27.0-35.0)  21.7±12.4^£^(46) | **MV**  7.0 (4.7-9.3)  4.1±3.0^£^(44)  **ICU**  10.0 (6.6-13.4)  11.4±10.4^£^(44)  **Hospital**  36.0 (29.9-42.1)  21.7±11.0^£^(44) | **NR** | **NR** | **NR** | **NR** |
| **9) Fetterplace 2018** | **28-day**  4/30 (13.3)  **60-day**  4/30 (13.3) | **28-day**  5/30 (16.7)  **60-day**  5/30 (16.7) | **NR** | **NR** | **MV**  8.7±7.5 (30)  **ICU**  10.6±8.3 (30)  **Hospital**  27.4±19.0 (30) | **MV**  7.0±5.0 (30)  **ICU**  9.1±5.5 (30)  **Hospital**  18.8±10 9 (30) | **QMLT loss at D15/ICUDC**  12.73±18.05 (24)^£^  **Best HGS kg at awakening or ICU DC or D15**  20±6.1 (6)  **MRC score** **at awakening or ICU DC or D15**  55±5.9 (7) | **QMLT loss at D15/ICUDC**  21.25±17.67 (23)^£^  **Best HGS kg at awakening or ICU DC or D15**  21±9.3 (16)  **MRC score** **at awakening or ICU DC or D15**  52±9.6 (14) | **Scored Physical Function in ICU Test**  6.8±3.8 (8)  **Discharge to rehab facility**  12/30 (40%) | **Scored Physical Function in ICU Test**  7.9±3.4 (14)  **Discharge to rehab facility**  13/30 (43%) |
| **10) van Zanten 2018** | **28-day**  2/22 (9.1)  **42-day**  3/22 (13.6)  **ICU**  1/22 (4.5)  **Hospital**  2/22 (9.1) | **28-day**  3/22 (13.6)  **42-day**  3/22 (13.6)  **ICU**  2/22 (9.1)  **Hospital**  3/22 (13.6) | **NR** | **NR** | **MV**  10.0±8.7 (22)  **ICU**  18.4±13.4 (22)  **Hospital**  28.5±13.3 (22) | **MV**  7.4±5.4 (22)  **ICU**  18.3±12.7 (22)  **Hospital**  28.2±13.2 (22) | **NR** | **NR** | **NR** | **NR** |
| **11) Vega-Alava 2018** | **Not specified**  0/20 | **Not specified**  0/20 | **VAP**  0/20 (0) | **VAP**  5/20 (25) | **MV**  5.4 (no SD) (20) | **MV**  7.45 (no SD) (20) | **NR** | **NR** | **NR** | **NR** |
| **12) Azevedo 2019** | **ICU**  22/57 (38.5)  **Hospital**  26/57 (45.6) | **ICU**  28/63 (44.4)  **Hosp**  29/63 (46.0) | **NR** | **NR** | **MV**  9 (5-14) (57)  **ICU**  21 (13-33) (57) | **MV**  9 (5-14) (63)  **ICU**  18 (10-35) (63) | **HGS at ICU discharge, kg**  Male: 18 (15-25) (15)  Female: 8 (2-17) (9) | **HGS at ICU discharge, kg**  Male: 23.5 (13.7-32.0) (14)  Female: 14 (7-22.5) (13) | **SF-36 PCS 3-mo**  93.6±126.1 (55)  **SF-36 PCS 6-mo**  92.0±133.4 (52) | **SF-36 PCS 3-mo**  85.2±110.6 (59)  **SF-36 PCS 6-mo**  90.0±120.6 (58) |
| **13) Danielis 2019** | **ICU**  2/19 (11) | **ICU**  7/21 (33) | **NR** | **NR** | **MV**  9.1±7.5^£^(19)  **ICU**  14.5±7.2^£^(19) | **MV**  9.3±4.6^£^(21)  **ICU**  16.0±6.5^£^(21) | **NR** | **NR** | **NR** | **NR** |
| **14) Badjatia 2020** | **NR** | **NR** | **Hosp-acquired infection**  3/12 (25) | **Hosp-acquired infection**  6/13 (46) | **ICU**  18±7 (12) | **ICU**  20±8 (13) | **CT mid-thigh cross-sectional area atrophy, %**  6.5±4.1 (12) | **CT mid-thigh cross-sectional area atrophy, %**  12.5±6.4 (13) | **Modified Rankin Scale**  D14: 4 (2-4)  D90: 1 (0-2)  **SPPB**  D14: 2 (0-7.8)  D90: 12 (10-12)  **D90 Short-form NeuroQOL**  i) Fatigue: 29±15  ii) Lower extremity mobility: 90±8 (12)  iii) Cognition: 35±5 | **Modified Rankin Scale**  D14: 4 (3-5)  D90: 2 (1-3)  **SPPB**  D14: 1 (0-5)  D90: 9 (4-12)  **D90 Short-form NeuroQOL**  i) Fatigue: 41±28  ii) Lower extremity mobility:  73±27 (13)  iii) Cognition: 31±12 |
| **15) Bukhari 2020^1^** | **Hospital**  7/19 (36.8) | **Hospital**  3/14 (21.4) | **NR** | **NR** | **ICU**  9.38±6.80 (13)  **Hospital**  18.38±9.51 (13) | **ICU**  9.09±5.53 (11)  **Hospital**  24.73±14.29 (11) | **NR** | **NR** | **NR** | **NR** |
| **16) Chapple 2020** | **ICU**  12/58 (21)  **28-d**  12/56 (21)  **90-d**  14/55 (26) | **ICU**  10/58 (17)  **28-d**  14/57 (25)  **90-d**  15/56 (27) | **NR** | **NR** | **28-d MV free day**  18±9 (58)  **ICU**  13±13 (58)  **Hospital**  24±21 (58) | **28-d MV free day**  18±9 (58)  **ICU**  14±18 (58)  **Hospital**  26±32 (58) | **NR** | **NR** | **D90 EQ-5D-5L** (41)  i) Mobility: 2.2±1.3  ii) Self-care: 2±1.2  iii) Usual activities: 2.5±1.3  iv) Pain or discomfort: 1.8±0.9  v) Anxiety/ Depression: 1.8±0.9  vi) VAS: 60±32  **D90 Discharge to Rehab Facility**  5/41 (12.2%) | **D90 EQ-5D-5L** (41)  i) Mobility: 2±1.3  ii) Self-care: 1.7±1.3  iii) Usual activities: 2.1±1.3  iv) Pain or discomfort: 1.9±0.9  v) Anxiety/ Depression: 1.8±0.9  vi) VAS: 53±32  **D90 Discharge to Rehab Facility**  6/41 (14.6%) |
| **17) Nakamura 2020** | **28-d Survival**  90.0% (n=60) | **28-d Survival**  89.5% (n=57) | **NR** | **NR** | **MV**  5.0±3.0^£^(60)  5 (2-6.5)  **ICU**  8.5±4.8^£^(60)  7 (5-12)  **Hospital**  43.5±39.3^£^(60)  26.5 (18-58) | **MV**  5.9±3.1^£^(57)  5.5 (3-9)  **ICU**  9.6±5.1^£^(57)  9 (6-13)  **Hospital**  50.4±35.6^£^(57)  45.5 (18.25-75.75) | **Femoral muscle volume loss, %**  12.9±8.5 (60) | **Femoral muscle volume loss, %**  16.9±7.0 (57) | **FSS-ICU ICU discharge**  15.5 (3-30.75)  **Barthel Index Hospital discharge**  62.5 (0-91.25)  **EQ-5D Hospital discharge**  8 (5-14.5) | **FSS-ICU ICU discharge**  18 (2.5-35)  **Barthel Index Hospital discharge**  12.5 (1-93.75)  **EQ-5D Hospital discharge**  8 (5-14) |
| **18) Carteron 2021** | **28-d**  20/100 (20)  **60-d**  23/100 (23) | **28-d**  21/95 (22)  **60-d**  23/95 (24) | **Pneumonia**  47/100 (47) | **Pneumonia**  41/95 (43) | **MV**  10 (6-16)  12±9^£^(100)  **ICU**  14 (8-21)  16±11^£^(100) | **MV**  11 (6-17)  13±9^£^(95)  **ICU**  15 (10-23)  18±11^£^(95) | **NR** | **NR** | **NR** | **NR** |
| **19) Dresen 2021** | **ICU**  8/21 (38)  **28-d**  2/21 (9.5) | **ICU**  7/21 (33)  **28-d**  4/21 (19.0) | **Pneumonia in ICU**  19/21 (90)  **Wound infection in ICU**  11/21 (52) | **Pneumonia in ICU**  17/21 (81)  **Wound infection in ICU**  11/21 (52) | **MV in ICU**  1372±642h  ~57.2±26.8d (21)  **MV in study period**  797±133h  ~33.2±5.5d (21)  **ICU**  68±34d (21) | **MV in ICU**  1350±1170h  ~56.3±48.8d (21)  **MV in study period**  758±191h  ~31.6±8.0d (21)  **ICU**  62±48d (21) | **QMLT mid right, mm**  -0.19±0.13  **QMLT, mid left, mm**  -0.18±0.10  **QMLT 2/3 right, mm**  -0.13±0.08  **QMLT, 2/3 left, mm**  -0.08±0.06  **Mean of all 4 measurements, mm (daily changes)**  -0.15±0.08  **Mean decrease of all 4 measurements in 28 days**  -3.4±1.8 mm  (30.4±11.7%) (15) | **QMLT mid right, mm**  -0.36±0.12  **QMLT, mid left, mm**  -0.24±0.10  **QMLT 2/3 right, mm**  -0.28±0.08  **QMLT, 2/3 left, mm**  -0.21±0.06  **Mean of all 4 measurements, mm (daily changes)**  -0.28±0.08  **Mean decrease of all 4 measurements in 28 days**  -5.7±2.5 mm  (51.8±21.1%) (12) | **NR** | **NR** |

CT: computed tomography, ECOG: Eastern Collaborative Oncology Group, FSS-ICU: functional status score in the ICU, HGS: handgrip strength, ICU: intensive care unit, MRC: medical research council, NR: not reported, PCS: physical component score, QMLT: quadriceps muscle layer thickness, RAND: public domain version of the Short Form 36, SF-36: short-form 36 quality of life questionnaire, SPPB: short physical performance battery, VAP: ventilator-associated pneumonia, VAS: Visual Analogue Scale

£: Information obtained from the author

^1^Bukhari 2020 has 3 groups: control (n=22), high-protein polymeric (n=19) and oligomeric group (n=14), the control group was excluded from the analysis. The result of TBI and Non-TBI group were combined.

Note: continuous data are presented as mean±SD (n) or median (quartile 1-qurtile 3) (n), unless otherwise stated.

**Table S8: Summary of results of meta-analysis**

**a) The main analysis: with *a priori* subgroup analysis of studies that used EN vs PN/IV AA strategy to alter protein delivery between groups**

| **Outcomes** | **Trials** | **N (higher/ lower protein)** | **I^2^ (%)** | **RR/MD/SMD (95% CI)** | **p-value** |
| --- | --- | --- | --- | --- | --- |
| **Overall Mortality** | 15 | 749/743 | 0 | 0.91 (0.75, 1.10) | 0.34 |
| EN strategy  PN/IV AA strategy  Test for subgroup differences: Chi^2^ = 0.05 , df = 1 (p=0.82) , I^2^ = 0% | 12  3 | 443/442  306/301 | 0  0 | 0.90 (0.71, 1.13)  0.94 (0.67, 1.33) | 0.36  0.74 |
| **ICU Mortality** | 9 | 507/512 | 0 | 0.94 (0.74, 1.20) | 0.63 |
| EN strategy  PN/IV AA strategy  Test for subgroup differences: Chi^2^ = 0.12 , df = 1 (p=0.73) , I^2^ = 0% | 6  3 | 201/211  306/301 | 0  0 | 0.91 (0.67, 1.24)  1.00 (0.66, 1.51) | 0.55  1.00 |
| **Hospital Mortality** | 5 | 396/394 | 0 | 0.98 (0.76, 1.26) | 0.86 |
| EN strategy  PN/IV AA strategy  Test for subgroup differences: Chi^2^ = 0.09 , df = 1 (p=0.76) , I^2^ = 0% | 3  2 | 98/99  298/295 | 0  9 | 1.03 (0.72, 1.47)  0.94 (0.64, 1.40) | 0.89  0.77 |
| **28-day Mortality** | 7 | 314/308 | 0 | 0.83 (0.60, 1.15) | 0.26 |
| No subgroup of study that used PN/IV AA strategy |  |  |  |  |  |
| **≥60-day Mortality** | 7 | 515/512 | 0 | 0.99 (0.78, 1.24) | 0.91 |
| EN strategy  PN/IV AA strategy  Test for subgroup differences: Chi^2^ = 0.21 , df = 1 (p=0.65) , I^2^ = 0% | 5  2 | 220/217  295/295 | 0  56 | 0.96 (0.70, 1.32)  1.13 (0.61, 2.08) | 0.81  0.70 |
| **Infectious Complications** | 7 | 233/229 | 0 | 1.05 (0.88, 1.25) | 0.59 |
| No subgroup of study that used PN/IV AA strategy |  |  |  |  |  |
| **Length of Mechanical Ventilation** | 10 | 421/416 | 8 | -0.57 (-1.29, 0.14) | 0.12 |
| EN strategy  PN/IV AA strategy  Test for subgroup differences: Chi^2^ = 2.02 , df = 1 (p=0.16) , I^2^ = 50.5% | 9  1 | 362/357  59/60 | 0  N/A | -0.73 (-1.39, -0.07)  2.20 (-1.78, 6.18) | 0.03  0.28 |
| **Length of ICU Stay** | 13 | 504/498 | 0 | -0.76 (-1.75, 0.23) | 0.13 |
| EN strategy  PN/IV AA strategy  Test for subgroup differences: Chi^2^ = 2.48 , df = 1 (p=0.12) , I^2^ = 59.7% | 12  1 | 445/439  59/60 | 0  N/A | -0.95 (-1.97, 0.07)  2.58 (-1.69, 6.85) | 0.07  0.24 |
| **Length of Hospital Stay** | 7 | 288/282 | 22 | 0.34 (-3.32, 4.00) | 0.85 |
| EN strategy  PN/IV AA strategy  Test for subgroup differences: Chi^2^ = 0.33 , df = 1 (p=0.56) , I^2^ = 0% | 6  1 | 229/222  59/60 | 32  N/A | -0.01 (-4.08, 4.06)  4.05 (-9.11, 17.21) | 1.00  0.55 |
| **Percentage Muscle Loss (per week)** | 5 | 139/134 | 16 | -3.44 (-4.99, -1.90) | <0.0001 |
| EN strategy  PN/IV AA strategy  Test for subgroup differences: Chi^2^ = 2.48 , df = 1 (p=0.12) , I^2^ = 59.7% | 4  1 | 111/105  28/29 | 0  N/A | -3.37 (-4.66, -2.07)  38.4 (-13.56, 90.36) | <0.00001  0.15 |
| **Percentage Muscle Loss (sensitivity analysis using SMD measure)** | 5 | 139/134 | 73 | -0.52 (-1.03, -0.00) | 0.05 |
| EN strategy  PN/IV AA strategy  Test for subgroup differences: Chi^2^ = 10.96 , df = 1 (p=0.0009) , I^2^ = 90.9% | 4  1 | 111/105  28/29 | 22  N/A | -0.68 (-1.02, -0.34)  0.37 (-0.15, 0.90) | <0.0001  0.16 |
| **Handgrip Strength** | 2 | 58/72 | 24 | 2.08 (-2.16, 6.32) | 0.34 |
| EN strategy  PN/IV AA strategy  Test for subgroup differences: Chi^2^ = 1.32 , df = 1 (p=0.25) , I^2^ = 24.1% | 1  1 | 6/16  52/56 | N/A  N/A | -1.00 (-7.68, 5.68)  3.60 (-0.53, 7.73) | 0.77  0.09 |
| **Discharge to Rehabilitation Facility** | 3 | 86/87 | 0 | 1.05 (0.74, 1.50) | 0.77 |
| No subgroup of study that used PN/IV AA strategy |  |  |  |  |  |
| **Quality of Life Physical Measures (SMD)** | 4 | 300/293 | 55 | 0.07 (-0.22, 0.36) | 0.63 |
| EN strategy  PN/IV AA strategy  Test for subgroup differences: Chi^2^ = 3.61 , df = 1 (p=0.06) , I^2^ = 72.3% | 3  1 | 108/113  192/180 | 24  N/A | 0.20 (-0.12, 0.52)  -0.16 (-0.37, 0.04) | 0.21  0.11 |

AA: amino acids, EN: enteral nutrition, ICU: intensive care unit, IV: intravenous, MD: mean difference, PN: Parenteral nutrition, RR: risk ration, SMD: standardized mean difference

**b) Sensitivity Analysis without Doig 2015**

| **Outcomes** | **Trials** | **N (higher/ lower protein)** | **I^2^ (%)** | **RR/MD/SMD (95% CI)** | **p-value** |
| --- | --- | --- | --- | --- | --- |
| Overall Mortality | 14 | 510/508 | 0 | 0.93 (0.75, 1.16) | 0.52 |
| ICU Mortality | 8 | 268/277 | 0 | 0.95 (0.71, 1.26) | 0.72 |
| Hospital Mortality | 4 | 157/159 | 0 | 1.08 (0.78, 1.50 | 0.66 |
| ≥60-day mortality | 6 | 279/277 | 0 | 1.05 (0.78, 1.41) | 0.74 |
| Quality of Life Physical Measures (SMD) | 3 | 108/113 | 24 | 0.20 (-0.12, 0.52) | 0.21 |

**c) Post-hoc subgroup analysis: subgroup analysis of studies that enrolled heterogenous patient population vs patients with head/brain pathology**

| **Outcomes** | **Trials** | **N (higher/ lower protein)** | **I^2^ (%)** | **RR/MD/SMD (95% CI)** | **p-value** |
| --- | --- | --- | --- | --- | --- |
| **Overall Mortality** | 14 | 741/737 | 0 | 0.91 (0.75,1.10) | 0.33 |
| Heterogenous patients  Patients with head/brain pathology  Test for subgroup differences: Chi^2^ = 0.11 , df = 1 (p=0.74) , I^2^ = 0% | 11  3 | 606/606  135/131 | 0  0 | 0.92 (0.74, 1.14)  0.85 (0.54, 1.32) | 0.46  0.30 |
| **ICU Mortality** | 9 | 507/512 | 0 | 0.94 (0.74, 1.20) | 0.63 |
| No subgroup of study that enrol only patients with head/brain pathology |  |  |  |  |  |
| **Hospital Mortality** | 5 | 396/394 | 0 | 0.98 (0.76, 1.26) | 0.86 |
| No subgroup of study that enrol only patients with head/brain pathology |  |  |  |  |  |
| **28-day Mortality** | 7 | 314/308 | 0 | 0.83 (0.60, 1.15) | 0.26 |
| Heterogenous patients  Patients with head/brain pathology  Test for subgroup differences: Chi^2^ = 0.01 , df = 1 (p=0.92) , I^2^ = 0% | 5  2 | 189/187  125/121 | 0  0 | 0.82 (0.51, 1.31)  0.84 (0.54, 1.32) | 0.40  0.46 |
| **≥60-day Mortality** | 7 | 515/512 | 0 | 0.99 (0.78, 1.24) | 0.91 |
| Heterogenous patients  Patients with head/brain pathology  Test for subgroup differences: Chi^2^ = 0.00 , df = 1 (p=0.99) , I^2^ = 0% | 4  3 | 380/381  135/131 | 0  0 | 0.99 (0.74, 1.31)  0.98 (0.67, 1.45) | 0.93  0.94 |
| **Infectious Complications** | 7 | 233/229 | 0 | 1.05 (0.88, 1.25) | 0.59 |
| Heterogenous patients  Patients with head/brain pathology  Test for subgroup differences: Chi^2^ = 0.11 , df = 1 (p=0.74) , I^2^ = 0% | 4  3 | 111/111  122/118 | 45  0 | 0.97 (0.66, 1.43)  1.05 (0.78, 1.41) | 0.87  0.74 |
| **Length of Mechanical Ventilation** | 10 | 421/416 | 8 | -0.57 (-1.29, 0.14) | 0.12 |
| Heterogenous patients  Patients with head/brain pathology  Test for subgroup differences: Chi^2^ = 1.10 , df = 1 (p=0.30) , I^2^ = 8.7% | 9  1 | 321/321  100/95 | 17  N/A | -0.43 (-1.26, 0.40)  -1.00 (-3.53, 1.53) | 0.31  0.44 |
| **Length of ICU Stay** | 13 | 504/498 | 0 | -0.76 (-1.75, 0.23) | 0.13 |
| Heterogenous patients  Patients with head/brain pathology  Test for subgroup differences: Chi^2^ = 0.91 , df = 1 (p=0.34) , I^2^ = 0% | 11  2 | 392/390  112/108 | 0  0 | -0.57 (-1.64,0.49)  -2.00 (-4.73, 0.73) | 0.29  0.15 |
| **Length of Hospital Stay** | 7 | 288/282 | 22 | 0.34 (-3.32, 4.00) | 0.85 |
| No subgroup of study that enrol only patients with head/brain pathology |  |  |  |  |  |
| **Percentage Muscle Loss (per week)** | 5 | 139/134 | 16 | -3.44 (-4.99, -1.90) | <0.0001 |
| Heterogenous patients  Patients with head/brain pathology  Test for subgroup differences: Chi^2^ = 0.30 , df = 1 (p=0.58) , I^2^ = 0% | 4  1 | 127/121  12/13 | 35  N/A | -3.95 (-6.66, -1.25)  -3.00 (-5.09, -0.91) | 0.004  0.005 |
| **Handgrip Strength** | 2 | 58/72 | 24 | 2.08 (-2.16, 6.32) | 0.34 |
| No subgroup of study that started intervention for >3 days of ICU admission |  |  |  |  |  |
| **Discharge to Rehabilitation Facility** | 3 | 86/87 | 0 | 1.05 (0.74, 1.50) | 0.77 |
| Heterogenous patients  Patients with head/brain pathology  Test for subgroup differences: Chi^2^ = 0.15 , df = 1 (p=0.70) , I^2^ = 0% | 2  1 | 74/74  12/13 | 0  NA | 1.10 (0.72,1.68)  0.95 (0.50,1.80) | 0.42  0.87 |
| **Quality of Life Physical Measures (SMD)** | 4 | 300/293 | 55 | 0.07 (-0.22, 0.36) | 0.63 |
| Heterogenous patients  Patients with head/brain pathology  Test for subgroup differences: Chi^2^ = 4.03 , df = 1 (p=0.04) , I^2^ = 75.2% | 3  1 | 288/280  12/13 | 17  NA | -0.05 (-0.25,0.14)  0.81 (-0.01,1.63) | 0.59  0.05 |

Note:

From Table 2, we have:

1. Nine studies included patients with mixed medical-surgical ICU population (Doig 2015, Ferrie 2015, Jakob 2017, Fetterplace 2018, van Zanten 2018, Azevedo 2019, Danielis 2019, Chapple 2020 and Nakamura 2020)
2. One study included heterogenous medical patient (Rugeles 2013)
3. One study included heterogenous surgical patient that stayed ≥10 days in the ICU (Dresen 2021)
4. Three studies did not specify the population clearly (Mesejo 2003, Vaga-Alava 2018, Bukhari 2020)
5. Four studies included patients with head/brain pathology (Clifton 1985, Zhou 2006, Badjatia 2020 and Carteron 2021)
6. One study included patients with acute renal failure (Singer 2007)

We performed a subgroup analysis for studies that enrolled heterogenous mixed medical-surgical, medical, or surgical patients (see above: a-c), we assumed that the 3 studies with unclear population also enrolled heterogenous patients (see above: d).

Another subgroup analysis was performed for the 4 studies that included patients with head/brain pathology (see above: e).

Singer 2017 (see above: f) was excluded from the subgroup analyses above because this is the only study that enrolled patient with acute renal failure.

**d) Post-hoc subgroup Analysis: subgroup analysis of studies that started intervention ≤3 vs >3 days of ICU admission**

| **Outcomes** | **Trials** | **N (higher/ lower protein)** | **I^2^ (%)** | **RR/MD/SMD (95% CI)** | **p-value** |
| --- | --- | --- | --- | --- | --- |
| **Overall Mortality** | 15 | 749/743 | 0 | 0.91 (0.75, 1.10) | 0.34 |
| ≤3 days of ICU admission  >3 days of ICU admission  Test for subgroup differences: Chi^2^ = 0.66 , df = 1 (p=0.42) , I^2^ = 0% | 12  3 | 693/686  56/57 | 0  0 | 0.93 (0.76, 1.14)  0.69 (0.35, 1.38) | 0.49  0.30 |
| **ICU Mortality** | 9 | 507/512 | 0 | 0.94 (0.74, 1.20) | 0.63 |
| ≤3 days of ICU admission  >3 days of ICU admission  Test for subgroup differences: Chi^2^ = 0.24 , df = 1 (p=0.62) , I^2^ = 0% | 8  1 | 486/491  21/21 | 0  N/A | 0.92 (0.71, 1.19)  1.14 (0.51, 2.58) | 0.54  0.75 |
| **Hospital Mortality** | 5 | 396/394 | 0 | 0.98 (0.76, 1.26) | 0.86 |
| No subgroup of study that started intervention for >3 days of ICU admission |  |  |  |  |  |
| **28-day Mortality** | 7 | 314/308 | 0 | 0.83 (0.60, 1.15) | 0.26 |
| ≤3 days of ICU admission  >3 days of ICU admission  Test for subgroup differences: Chi^2^ = 0.41 , df = 1 (p=0.52) , I^2^ = 0% | 5  2 | 268/261  46/47 | 0  0 | 0.88 (0.61, 1.27)  0.68 (0.33, 1.37) | 0.48  0.28 |
| **≥60-day Mortality** | 7 | 515/512 | 0 | 0.99 (0.78, 1.24) | 0.91 |
| ≤3 days of ICU admission  >3 days of ICU admission  Test for subgroup differences: Chi^2^ = 0.03 , df = 1 (p=0.86) , I^2^ = 0% | 5  2 | 480/476  35/36 | 0  0 | 0.98 (0.76, 1.25)  1.04 (0.56, 1.92) | 0.86  0.91 |
| **Infectious Complications** | 7 | 233/229 | 0 | 1.05 (0.88, 1.25) | 0.59 |
| ≤3 days of ICU admission  >3 days of ICU admission  Test for subgroup differences: Chi^2^ = 0.73 , df = 1 (p=0.39) , I^2^ = 0% | 5  2 | 202/198  31/31 | 11  0 | 0.96 (0.72, 1.27)  1.13 (0.88, 1.44) | 0.76  0.35 |
| **Length of Mechanical Ventilation** | 10 | 421/416 | 8 | -0.57 (-1.29, 0.14) | 0.12 |
| ≤3 days of ICU admission  >3 days of ICU admission  Test for subgroup differences: Chi^2^ = 1.10 , df = 1 (p=0.30) , I^2^ = 8.7% | 9  1 | 400/395  21/21 | 7  N/A | -0.65 (-1.37, 0.07)  1.60 (-2.55, 5.75) | 0.08  0.45 |
| **Length of ICU Stay** | 13 | 504/498 | 0 | -0.76 (-1.75, 0.23) | 0.13 |
| ≤3 days of ICU admission  >3 days of ICU admission  Test for subgroup differences: Chi^2^ = 0.28 , df = 1 (p=0.60) , I^2^ = 0% | 12  1 | 483/477  21/21 | 0  N/A | -0.77 (-1.76,0.22)  6.00 (-19.16, 31.16) | 0.13  0.64 |
| **Length of Hospital Stay** | 7 | 288/282 | 22 | 0.34 (-3.32, 4.00) | 0.85 |
| No subgroup of study that started intervention for >3 days of ICU admission |  |  |  |  |  |
| **Percentage Muscle Loss (per week)** | 5 | 139/134 | 16 | -3.44 (-4.99, -1.90) | <0.0001 |
| ≤3 days of ICU admission  >3 days of ICU admission  Test for subgroup differences: Chi^2^ = 1.60 , df = 1 (p=0.21) , I^2^ = 37.5% | 4  1 | 124/122  15/12 | 4  N/A | -2.99 (-4.48, -1.50)  -5.35 (-8.69, -2.01) | <0.00001  0.002 |
| **Handgrip Strength** | 2 | 58/72 | 24 | 2.08 (-2.16, 6.32) | 0.34 |
| No subgroup of study that started intervention for >3 days of ICU admission |  |  |  |  |  |
| **Discharge to Rehabilitation Facility** | 3 | 86/87 | 0 | 1.05 (0.74, 1.50) | 0.77 |
| No subgroup of study that started intervention for >3 days of ICU admission |  |  |  |  |  |
| **Quality of Life Physical Measures (SMD)** | 4 | 300/293 | 55 | 0.07 (-0.22, 0.36) | 0.63 |
| No subgroup of study that started intervention for >3 days of ICU admission |  |  |  |  |  |

**References**

1. Clifton GL, Robertson CS, Contant CF. Enteral hyperalimentation in head injury. *J Neurosurg*. 1985;62:186-193. doi:10.3171/jns.1985.62.2.0186

2. Mesejo A, Acosta JA, Ortega C, et al. Comparison of a high-protein disease-specific enteral formula with a high-protein enteral formula in hyperglycemic critically ill patients. *Clin Nutr*. 2003;22(3):295-305. doi:10.1016/S0261-5614(02)00234-0

3. Zhou C-P, Su Y. Effect of the Equal Non-protein-calorie but Different Protein Intake on Enteral Nutritional Metabolism in 51 Patients with Severe Stroke. A Randomized Controlled Study. *Chinese J Clin Nutr*. 2006;14(6):351-355.

4. Singer P. High-dose amino acid infusion preserves diuresis and improves nitrogen balance in non-oliguric acute renal failure. *Wien Klin Wochenschr*. 2007;119(7-8):218-222. doi:10.1007/s00508-007-0794-3

5. Rugeles SJ, Rueda J-D, Díaz C-E, Rosselli D. Hyperproteic hypocaloric enteral nutrition in the critically ill patient: A randomized controlled clinical trial. *Indian J Crit Care Med*. 2013;17(6):343-349. doi:10.4103/0972?5229.123438

6. Doig GS, Simpson F, Bellomo R, et al. Intravenous amino acid therapy for kidney function in critically ill patients: a randomized controlled trial. *Intensive Care Med*. 2015;41(7):1197-1208. doi:10.1007/s00134-015-3827-9

7. Ferrie S, Allman-Farinelli M, Daley M, Smith K. Protein requirements in the critically ill: a randomised controlled trial using parenteral nutrition. *JPEN J Parenter Enter Nutr*. 2016;40(6):795-805. doi:10.1177/0148607115618449

8. Jakob SM, Butikofer L, Berger D, Coslovsky M, Takala J. A randomized controlled pilot study to evaluate the effect of an enteral formulation designed to improve gastrointestinal tolerance in the critically ill patient-the SPIRIT trial. *Crit Care*. 2017;21:140. doi:10.1186/s13054-017-1730-1

9. Fetterplace K, Deane AM, Tierney A, et al. Targeted Full Energy and Protein Delivery in Critically Ill Patients: A Pilot Randomized Controlled Trial (FEED Trial). *JPEN J Parenter Enter Nutr*. 2018;42(8):1252-1262. doi:10.1002/jpen.1166

10. van Zanten ARH, Petit L, De Waele J, et al. Very high intact-protein formula successfully provides protein intake according to nutritional recommendations in overweight critically ill patients: a double-blind randomized trial. *Crit Care*. 2018;22(156):1-12.

11. Vega-Alava KM, Sy RAG, Domado AM. The effect of whey protein supplementation on duration of mechanical ventilation: A pilot study. *Philipp J Intern Med*. 2018;56(2):71-76.

12. Azevedo JRA de, Lima HCM, Montenegro WS, et al. Optimized calorie and high protein intake versus recommended caloric-protein intake in critically ill patients: a prospective, randomized, controlled phase II clinical trial. *Rev Bras Ter Intensiva*. 2019;31(2):171-179. doi:10.5935/0103-507X.20190025

13. Danielis M, Lorenzoni G, Azzolina D, et al. Effect of Protein-Fortified Diet on Nitrogen Balance in Critically Ill Patients: Results from the OPINiB Trial. *Nutrients*. 2019;11(5):972. doi:10.3390/nu11050972

14. Badjatia N, Sanchez S, Judd G, et al. Neuromuscular Electrical Stimulation and High‑Protein Supplementation After Subarachnoid Hemorrhage: A Single‑Center Phase 2 Randomized Clinical Trial. *Neurocrit Care*. 2020. doi:10.1007/s12028-020-01138-4

15. Bukhari A, Taslim NA, As’ad S, et al. Comparison of different early enteral feeding formulas on critically ill patients. *J Nutr Sci Vitaminol*. 2020;66:S2-S10. doi:10.3177/jnsv.66.S2

16. Chapple LS, Summers MJ, Bellomo R, et al. Use of a High Protein Enteral Nutrition Formula to Increase Protein Delivery to Critically Ill Patients: A Randomized, Blinded, Parallel‐group, Feasibility Trial. *JPEN J Parenter Enter Nutr*. 2020. doi:10.1002/jpen.2059

17. Nakamura K, Nakano H, Naraba H, et al. High protein versus medium protein delivery under equal total energy delivery in critical care: A randomized controlled trial. *Clin Nutr*. 2020. doi:10.1016/j.clnu.2020.07.036

18. Carteron L, Samain E, Winiszewski H, et al. Semi‑elemental versus polymeric formula for enteral nutrition in brain‑injured critically ill patients:a randomized trial. *Crit Care*. 2021;25(31):1-12. doi:10.1186/s13054-020-03456-7

19. Dresen E, Weißbrich C, Fimmers R, Putensen C, Stehle P. Medical high-protein nutrition therapy and loss of muscle mass in adult ICU patients: a randomized controlled trial. *Clin Nutr*. 2021. doi:10.1016/j.clnu.2021.02.021
