# Supplementary material for: The effect of higher versus lower protein delivery in critically ill patients: a systematic review and meta-analysis of randomized controlled trials
Source: Crit Care. 2021 Jul 23;25:260. doi: 10.1186/s13054-021-03693-4 (PMC8300989; doi:10.1186/s13054-021-03693-4)
Supplement: Supplementary file 3 — Additional file 3. Supplementary figures. [file 13054_2021_3693_MOESM3_ESM.docx]

**Supplementary figures**

**The Effect of Higher versus Lower Protein Delivery In Critically Ill Patients. A Systematic Review and Meta-Analysis of Randomized Controlled Trials.**

Zheng-Yii Lee, MSc^1^ [zheng_yii@hotmail.com](mailto:zheng_yii@hotmail.com)

Cindy Sing Ling Yap, BSc^1^ [cindyapsl@gmail.com](mailto:cindyapsl@gmail.com)

M. Shahnaz Hasan, MBBS, MAnaes^1^ [shahnaz@ummc.edu.my](mailto:shahnaz@ummc.edu.my)

Julia Patrick Engkasan, MBBS, MRehabMed, PhD^2^ [julia@ummc.edu.my](mailto:julia@ummc.edu.my)

Mohd Yusof Barakatun-Nisak, PhD^3,4^ [bnisak@upm.edu.my](mailto:bnisak@upm.edu.my)

Andrew G. Day, MSc^5^ [andrew.day@kingstonhsc.ca](mailto:andrew.day@kingstonhsc.ca)

Jayshil J. Patel, MD^6^ [jpatel2@mcw.edu](mailto:jpatel2@mcw.edu)

Daren K. Heyland, MSc, FRCPC^5^ [dkh2@queensu.ca](mailto:dkh2@queensu.ca)

^1^ Department of Anesthesiology, Faculty of Medicine, University of Malaya, Kuala Lumpur, Malaysia.

^2^ Department of Rehabilitation Medicine, Faculty of Medicine, University of Malaya, Kuala Lumpur, Malaysia.

^3^ Department of Nutrition and Dietetics, Faculty of Medicine and Health Sciences, Universiti Putra Malaysia, Serdang, Malaysia

^4^ Institute for Social Science Studies, Universiti Putra Malaysia, Selangor, Malaysia

^5^ Department of Critical Care Medicine, Queen’s University and the Clinical Evaluation Research Unit, Kingston General Hospital, Kingston, Ontario, Canada.

^6^ Medical College of Wisconsin, Milwaukee, Wisconsin, Unites States

**Corresponding Author**

Daren K. Heyland

Department of Critical Care Medicine, Queen’s University and the Clinical Evaluation Research Unit, Kingston General Hospital, Kingston, Ontario, Canada.

Email address: [dkh2@queensu.ca](mailto:dkh2@queensu.ca)

**
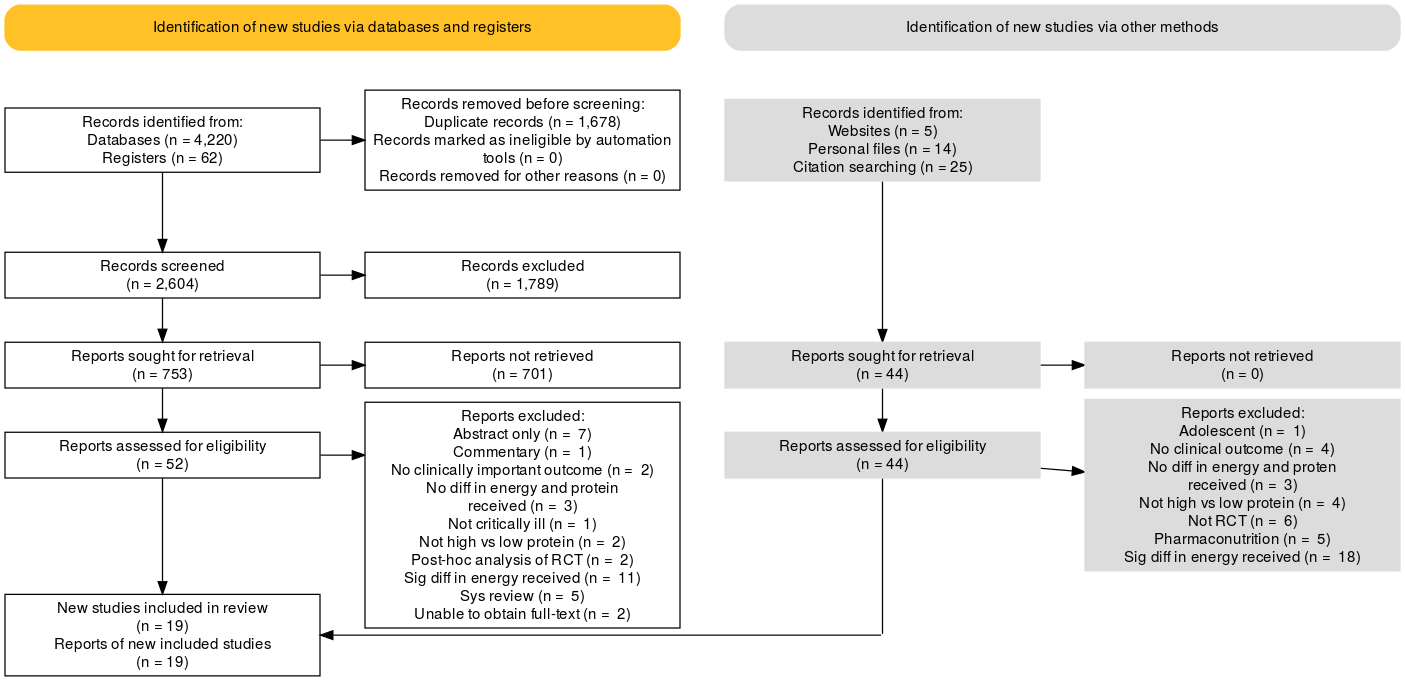
**

**Figure S1: PRISMA 2020 Flow Diagram**

| **a) Protein delivered (g/kg/day)**  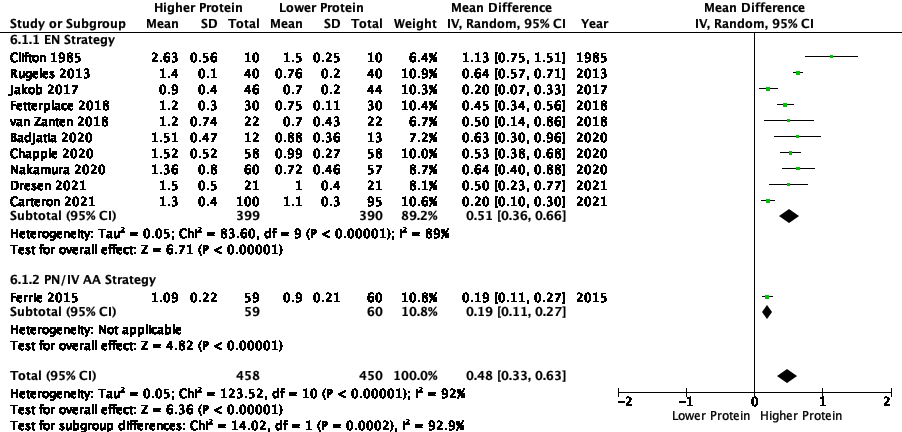  Pooled protein delivered between higher vs lower protein group: 1.31±0.48 vs 0.90±0.30 g/kg/d |
| --- |
| **b) Energy delivered (kcal/kg/day)**  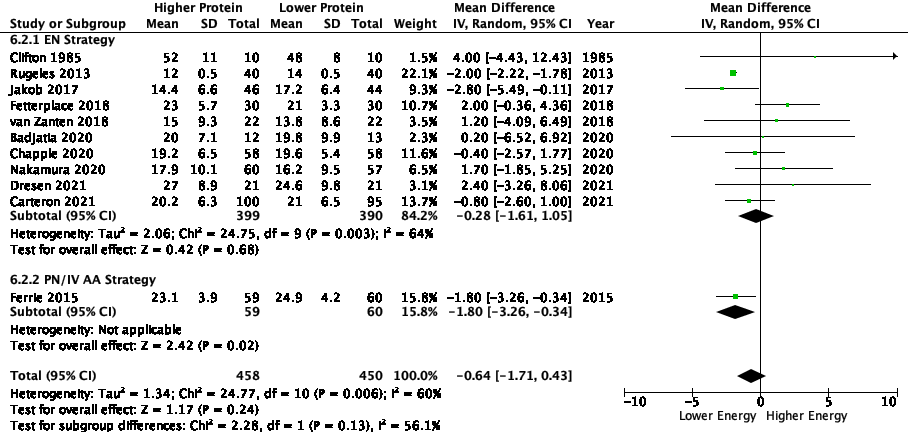  Pooled energy received between higher vs lower protein group: 19.87±6.93 vs 20.13±7.10 kcal/kg/d |

**Figure S2 Protein (g/kg/day) and Energy (kcal/kg/day) delivered between higher vs lower protein group**

| **a) Protein delivered (g/day)**  **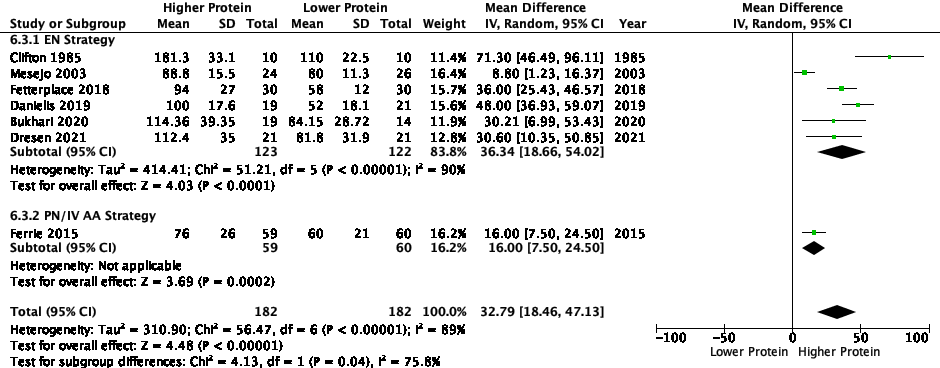**  Pooled protein delivered between higher vs lower protein group: 97.15±27.64 vs 68.72±20.78 g/d |
| --- |
| **b) Energy delivered (kcal/day)**  **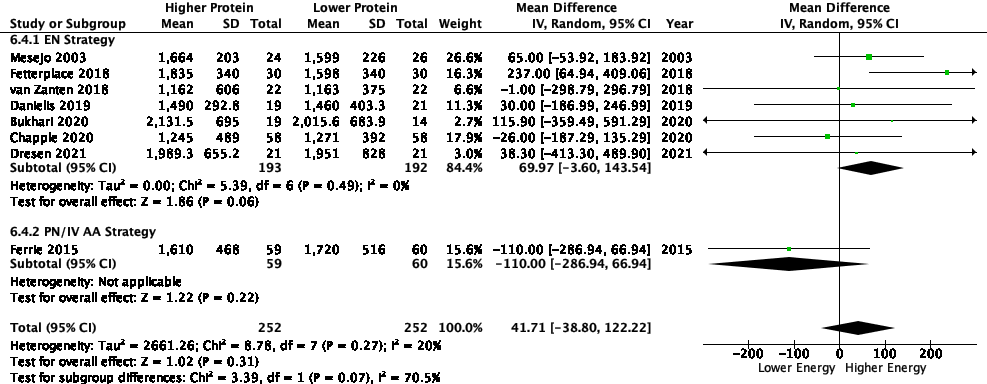**  Pooled energy delivered between higher vs lower protein group: 1580.69±484.05 vs 1555.03±475.19 kcal/d |

**Figure S3 Protein (g/day) and Energy (kcal/day) delivered between higher vs lower protein group**

| **a) Overall Mortality** | | |
| --- | --- | --- |
| **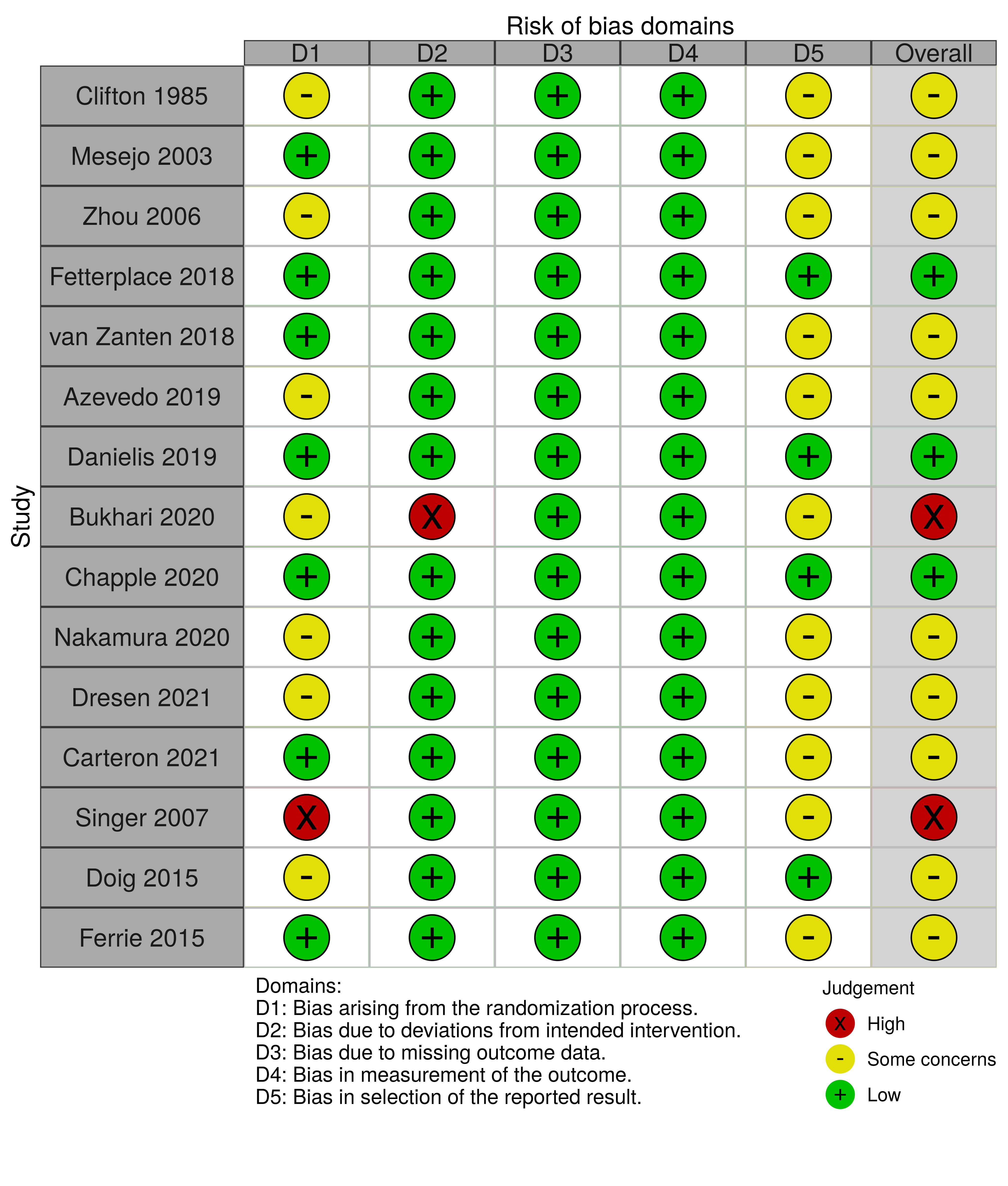** | **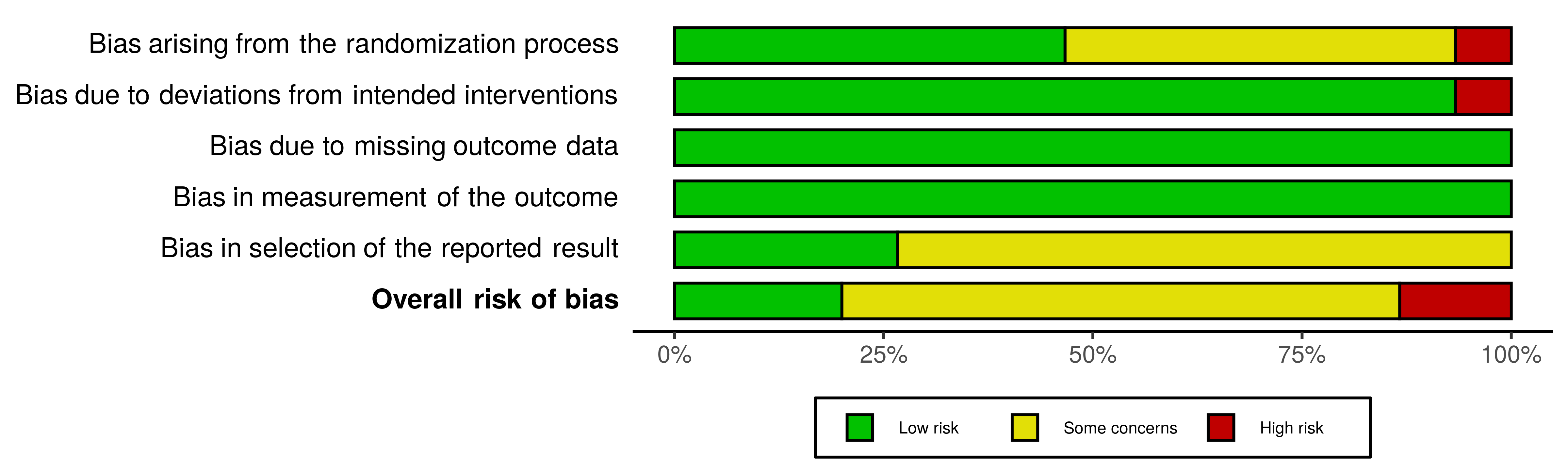** | |
| **b) ICU Mortality** | | |
| **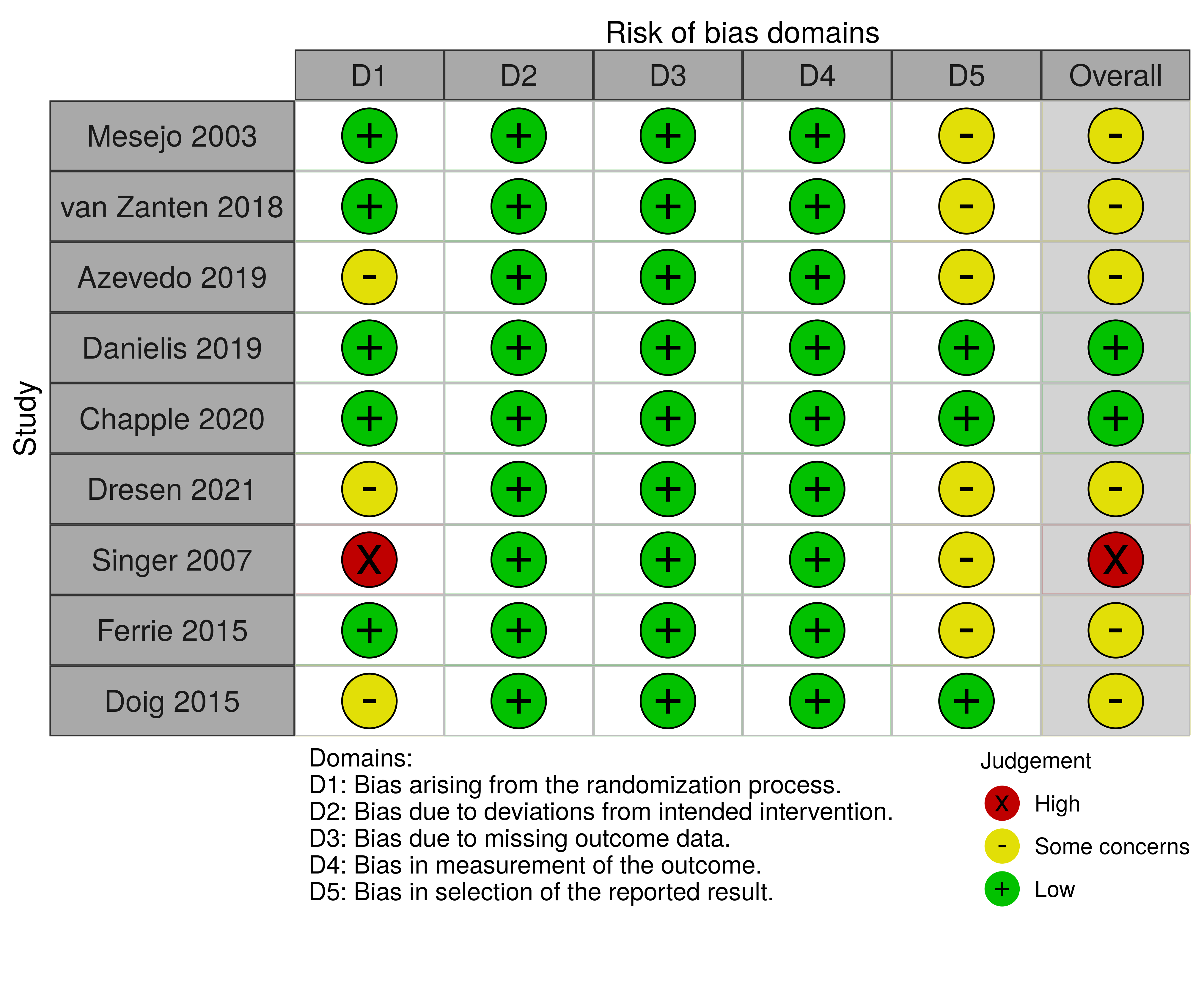** | **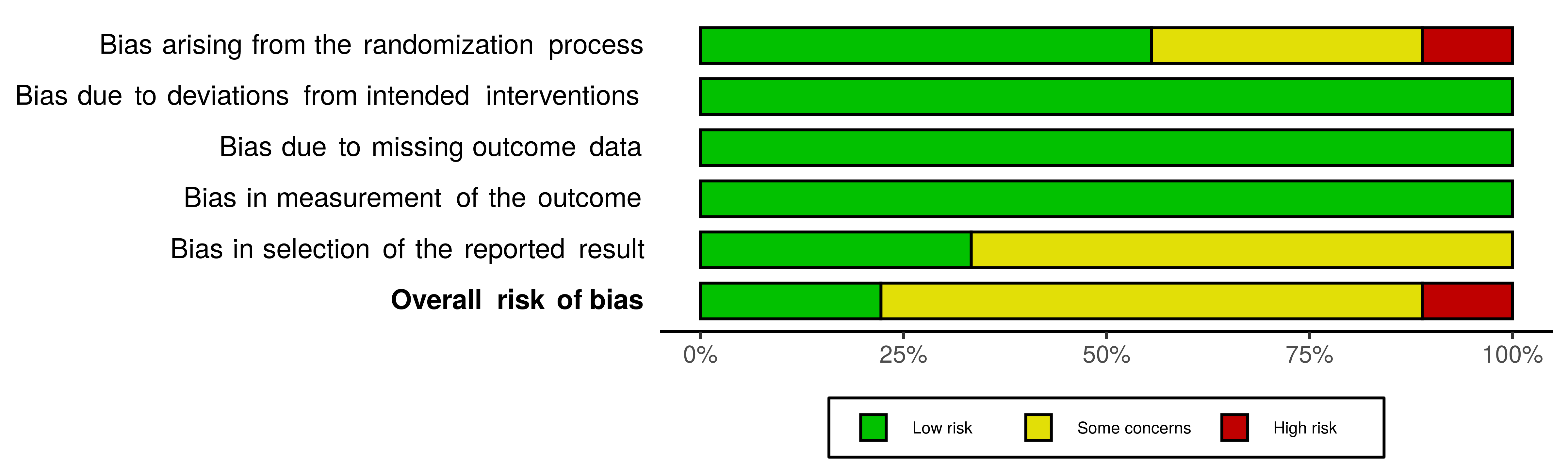** | |
| **c) Hospital Mortality** | | |
| **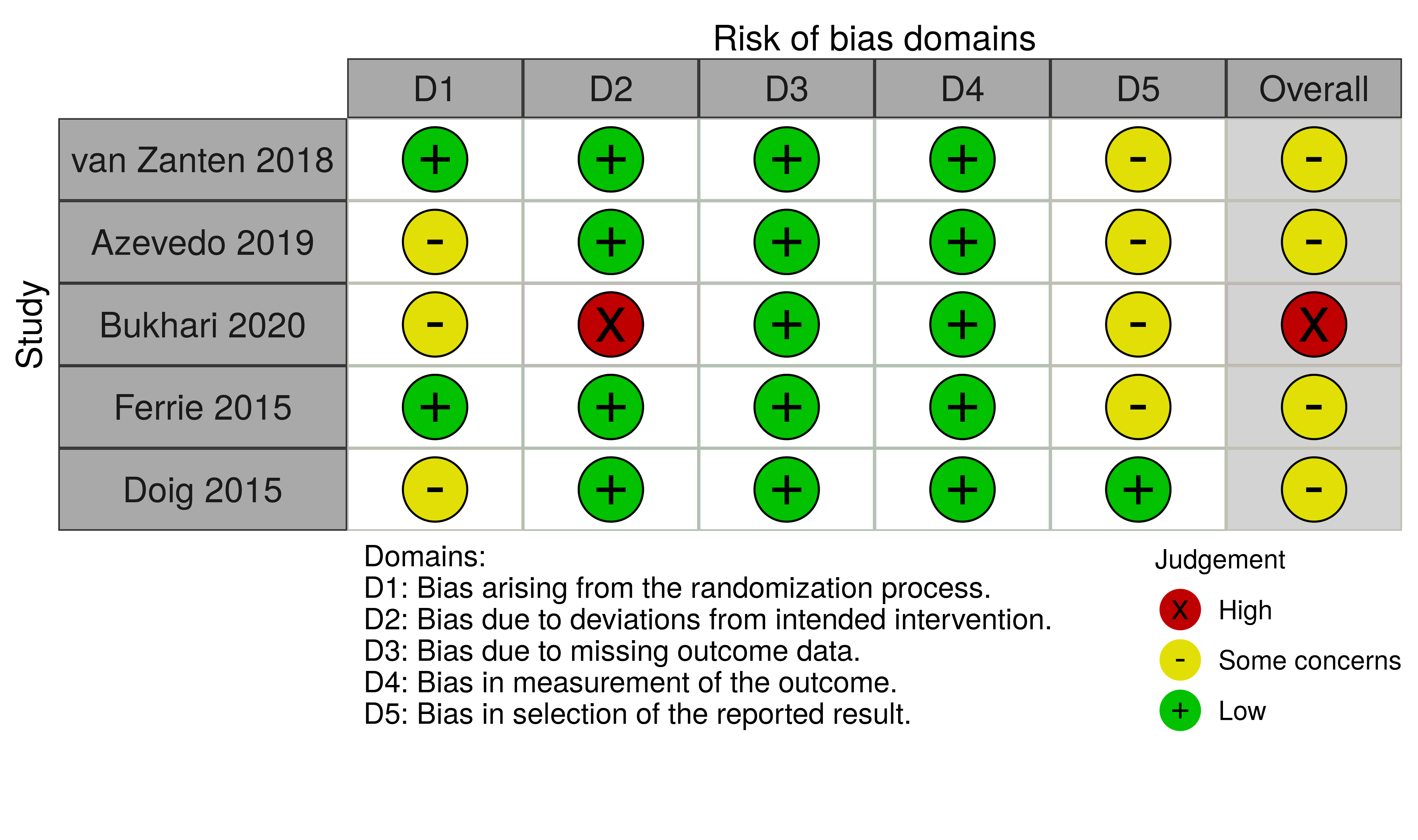** | **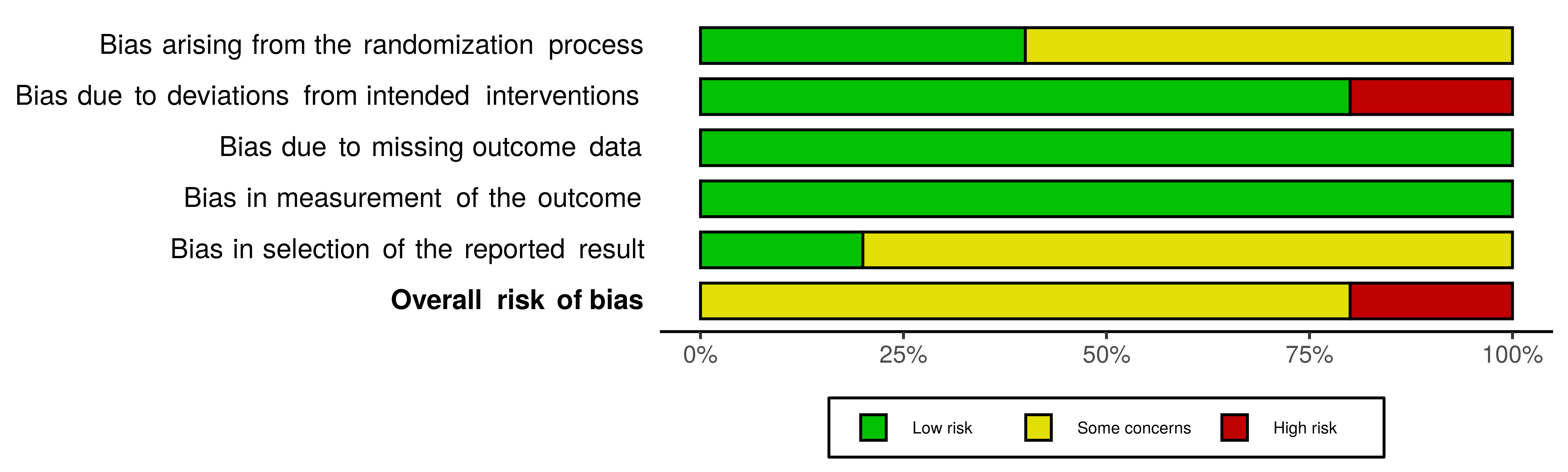** | |
| **d) 28-day mortality** | | |
| **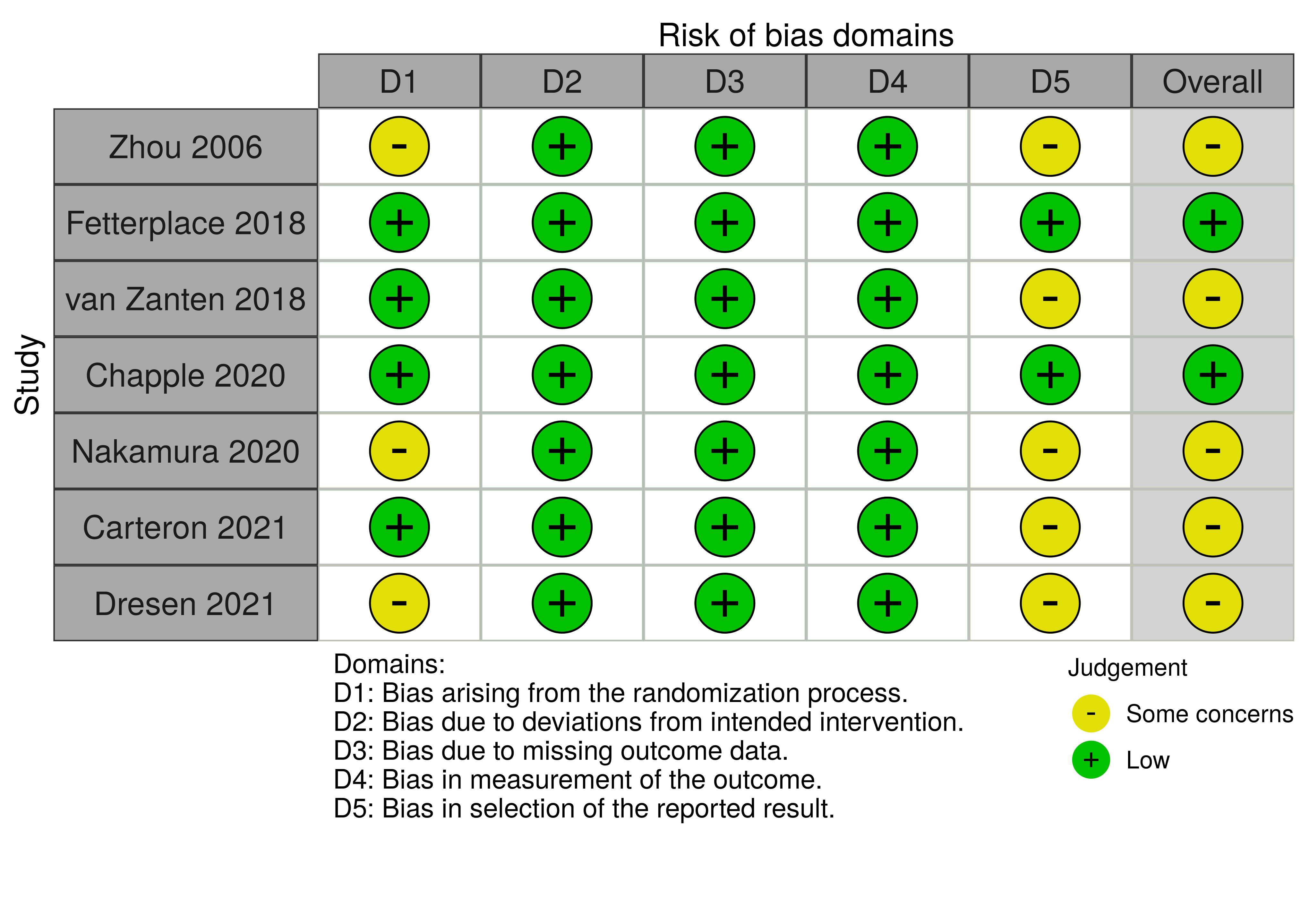** | **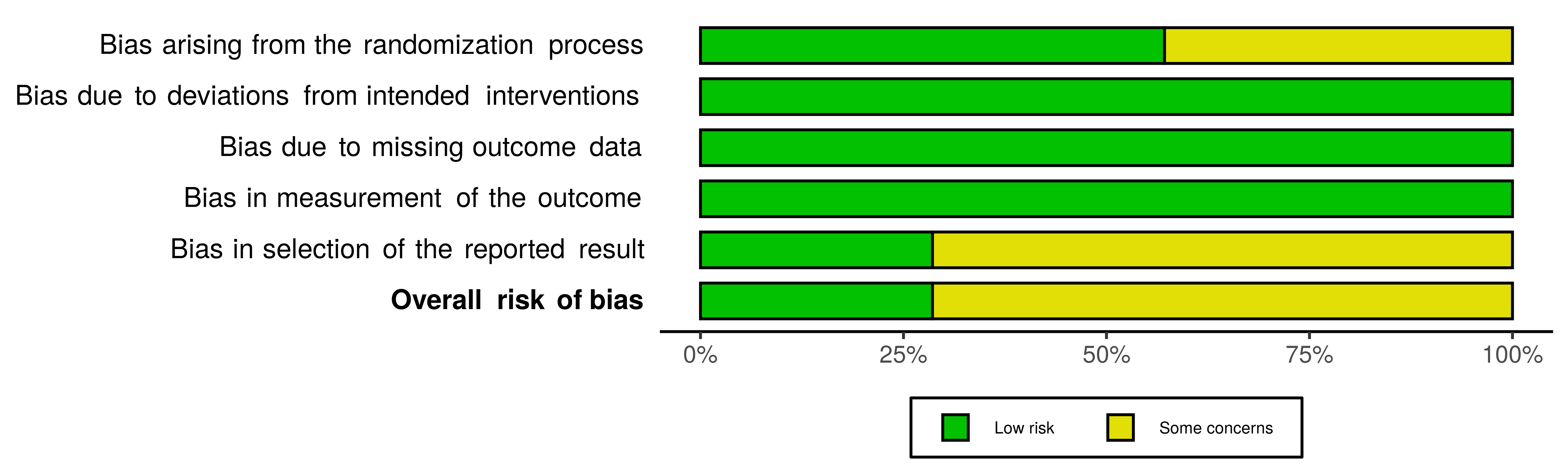** | |
| **e) 60-day mortality** | | |
| **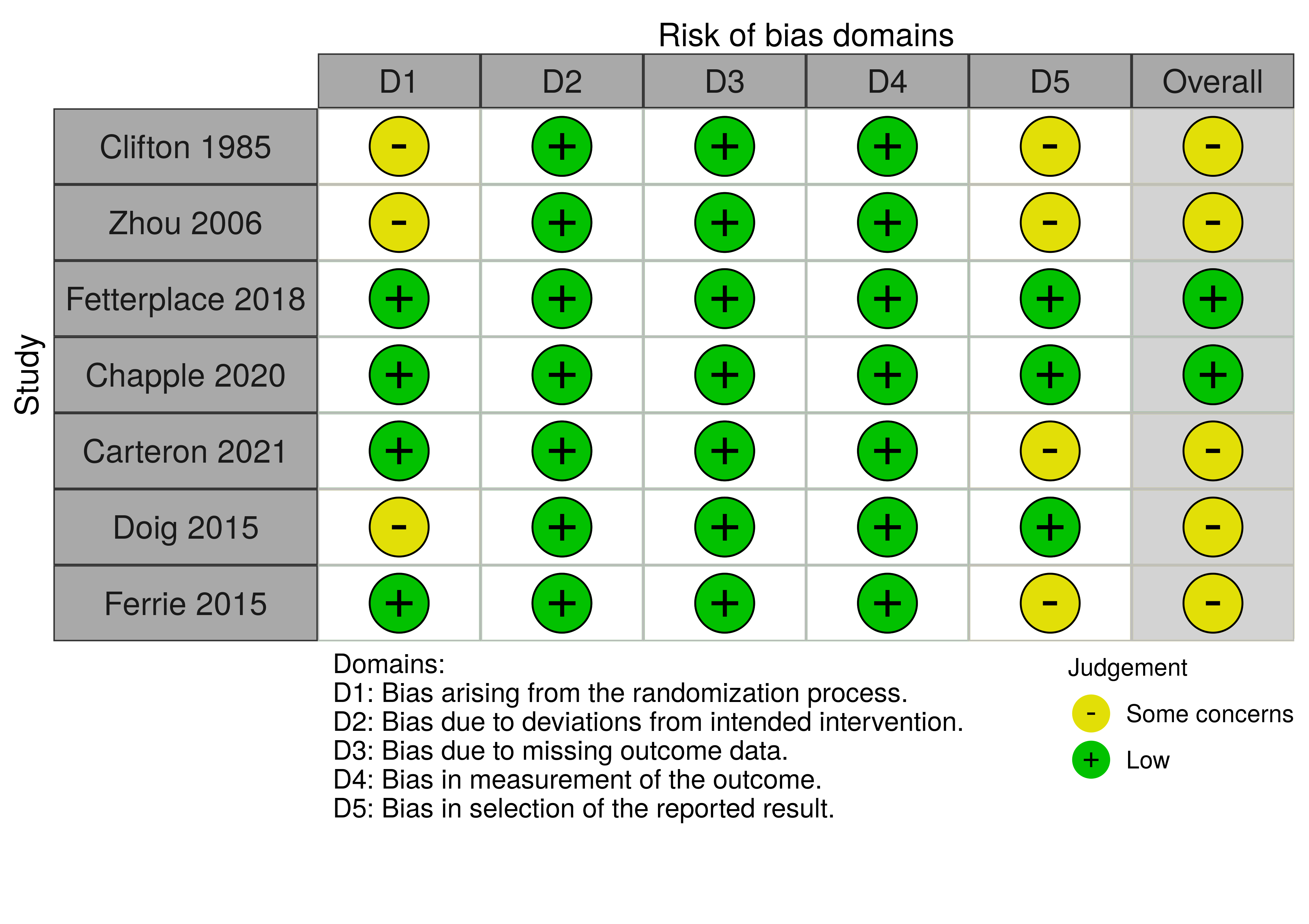** | **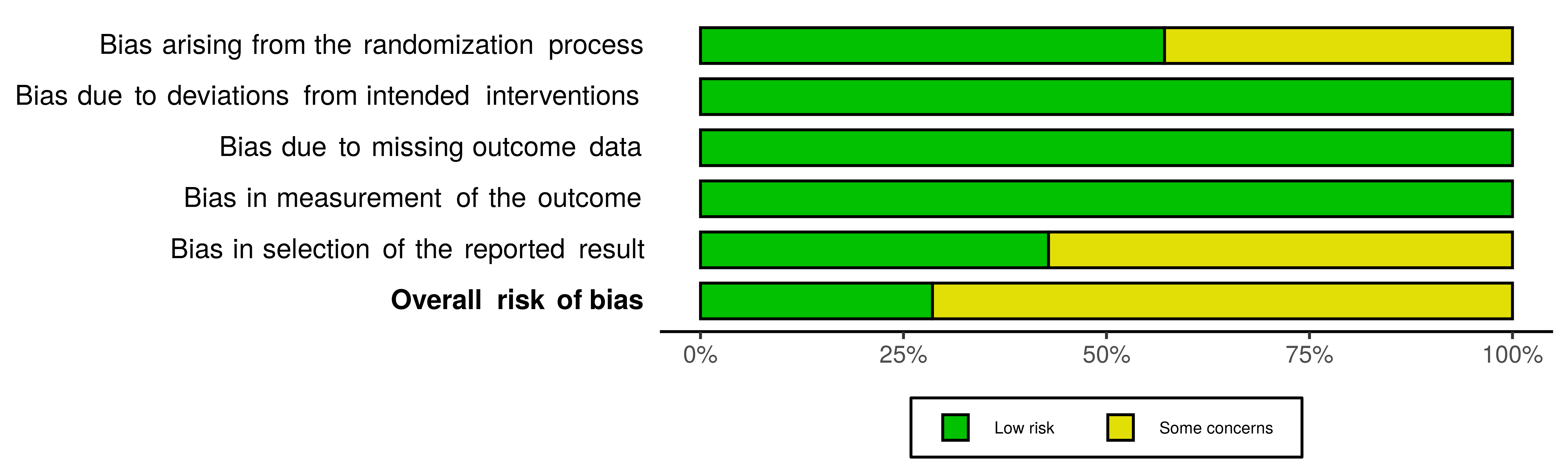** | |
| **f) Infectious complications** | | |
| **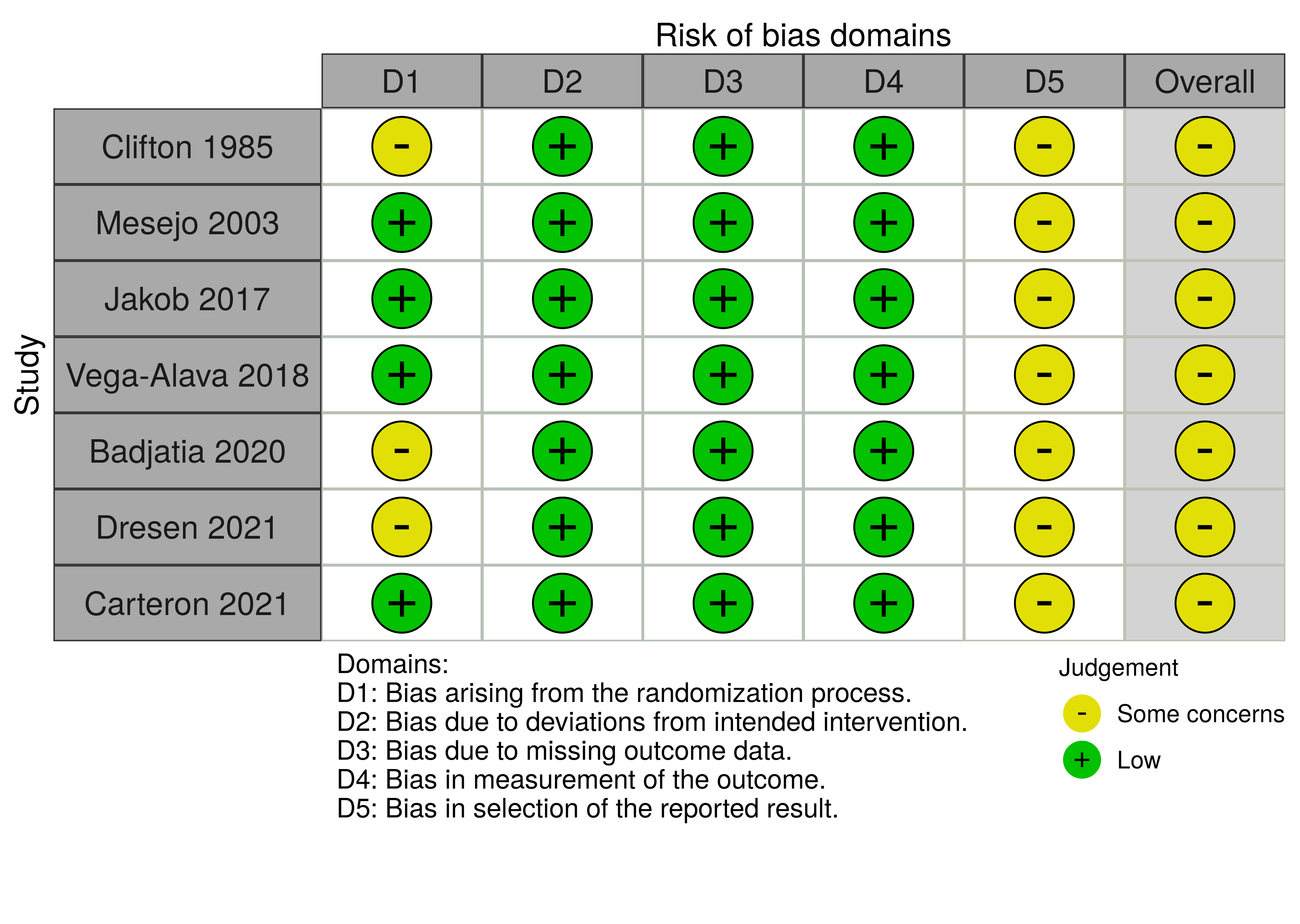** | | **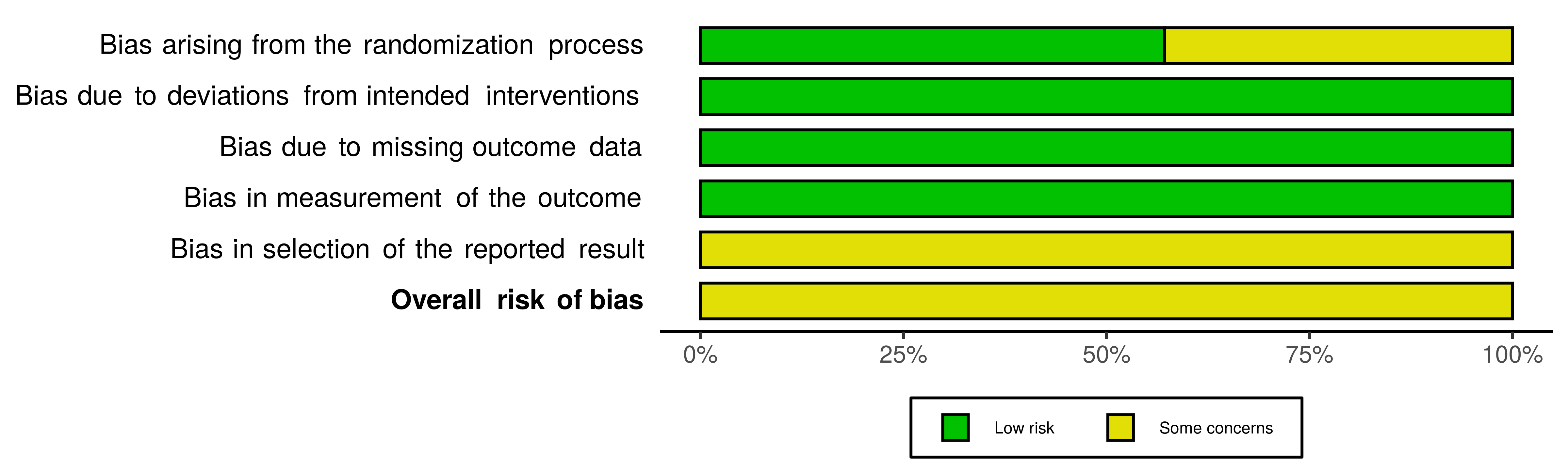** |
| **g) Duration of mechanical ventilation** | | |
| **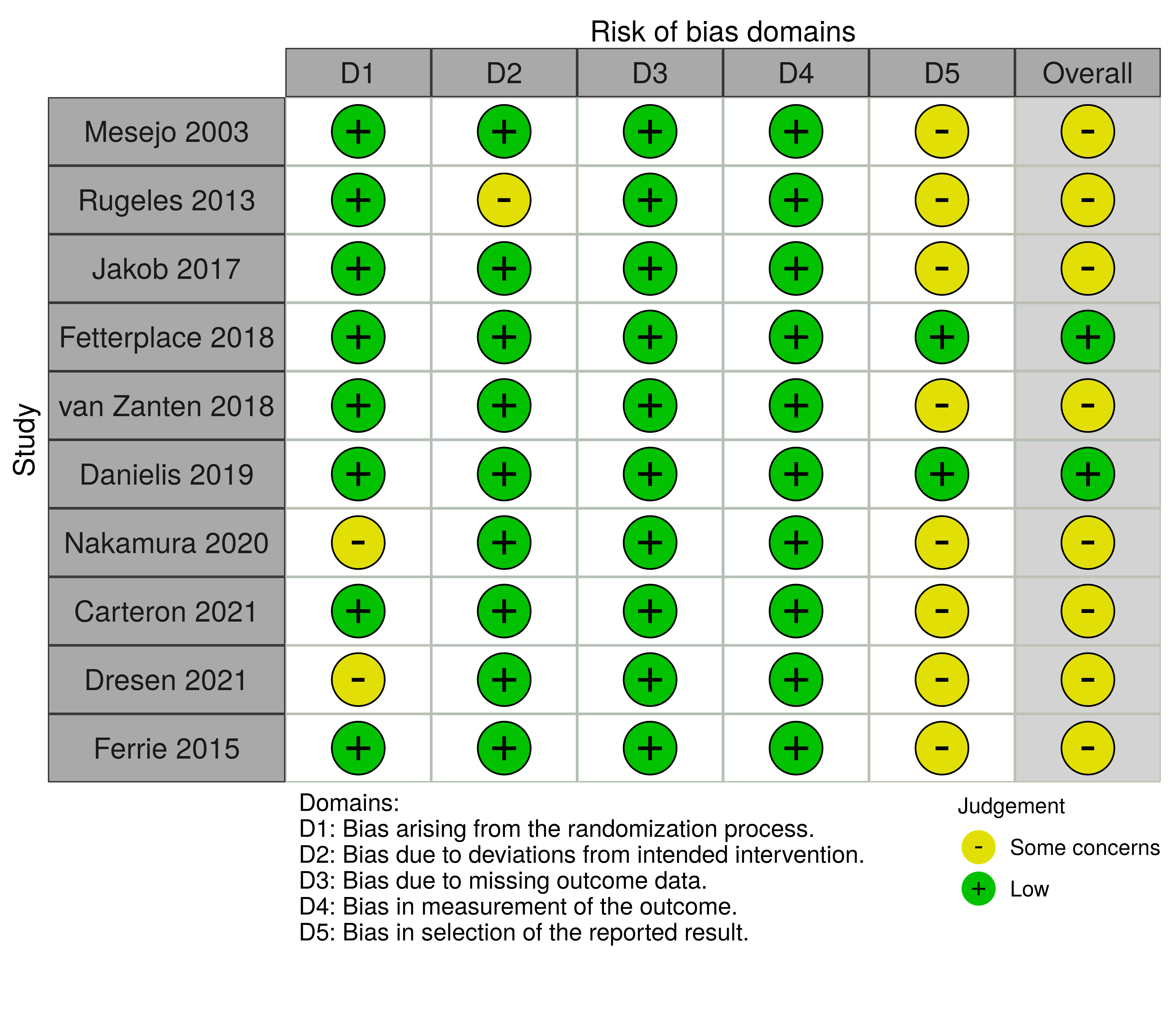** | | **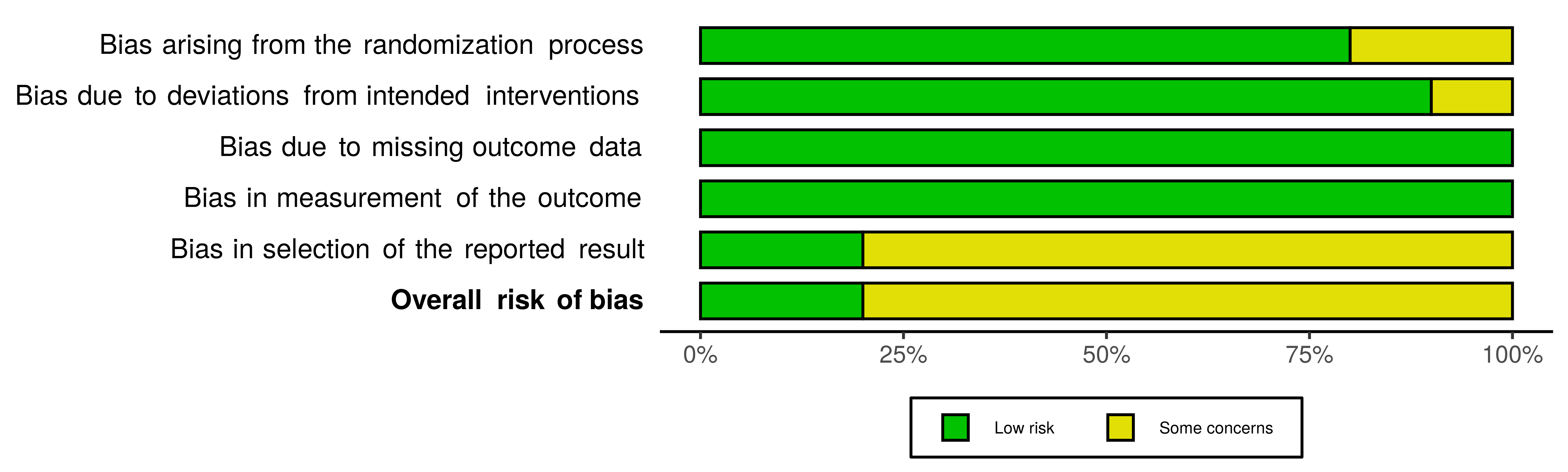** |
| **h) ICU length of stay** | | |
| **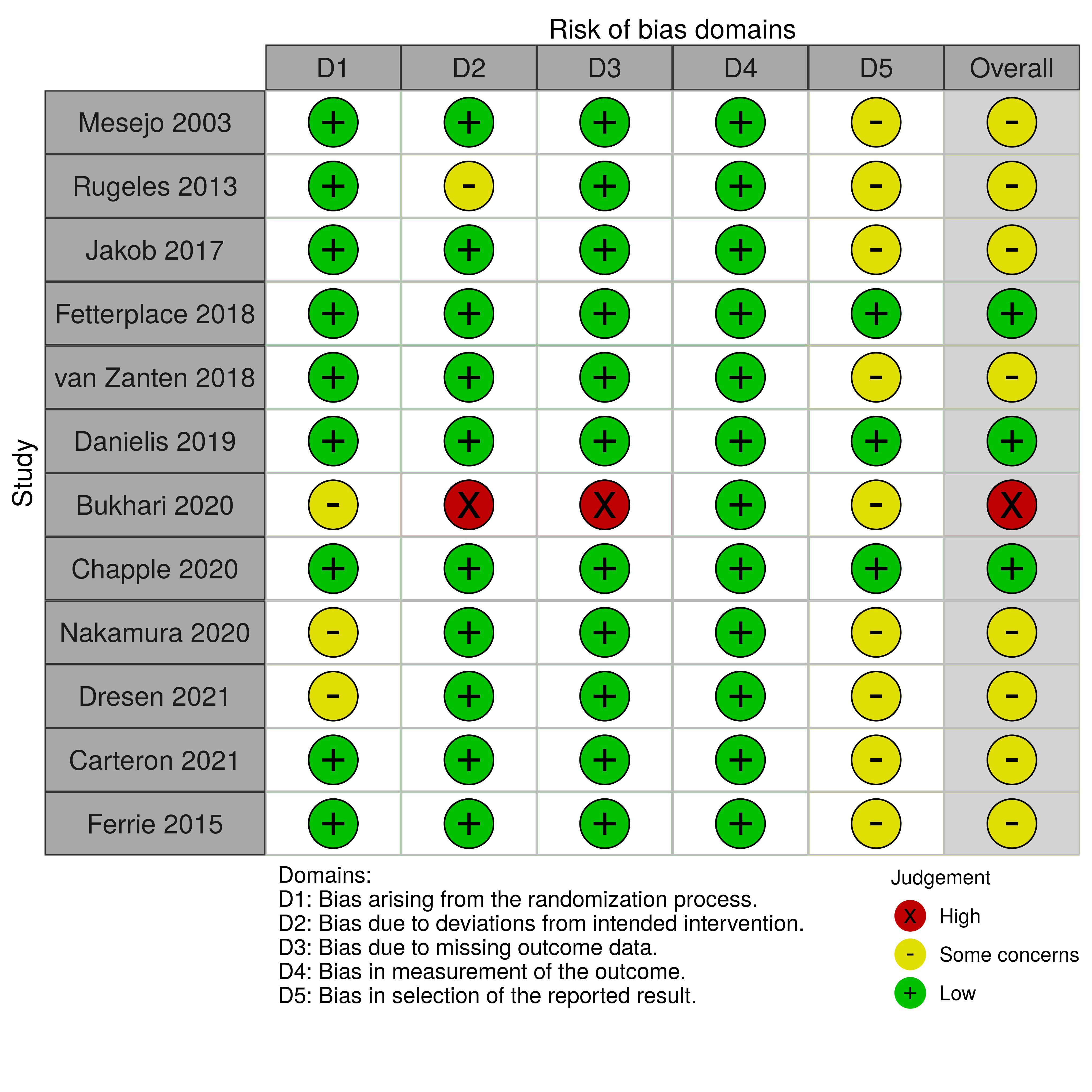** | | **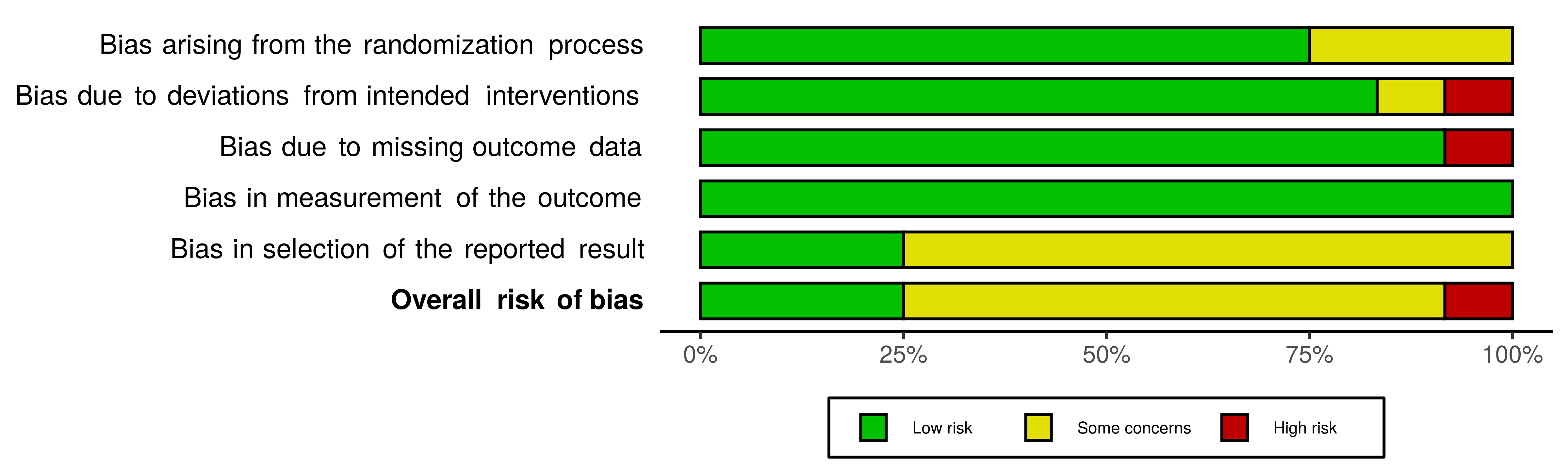** |
| **i) Hospital length of stay** | | |
| **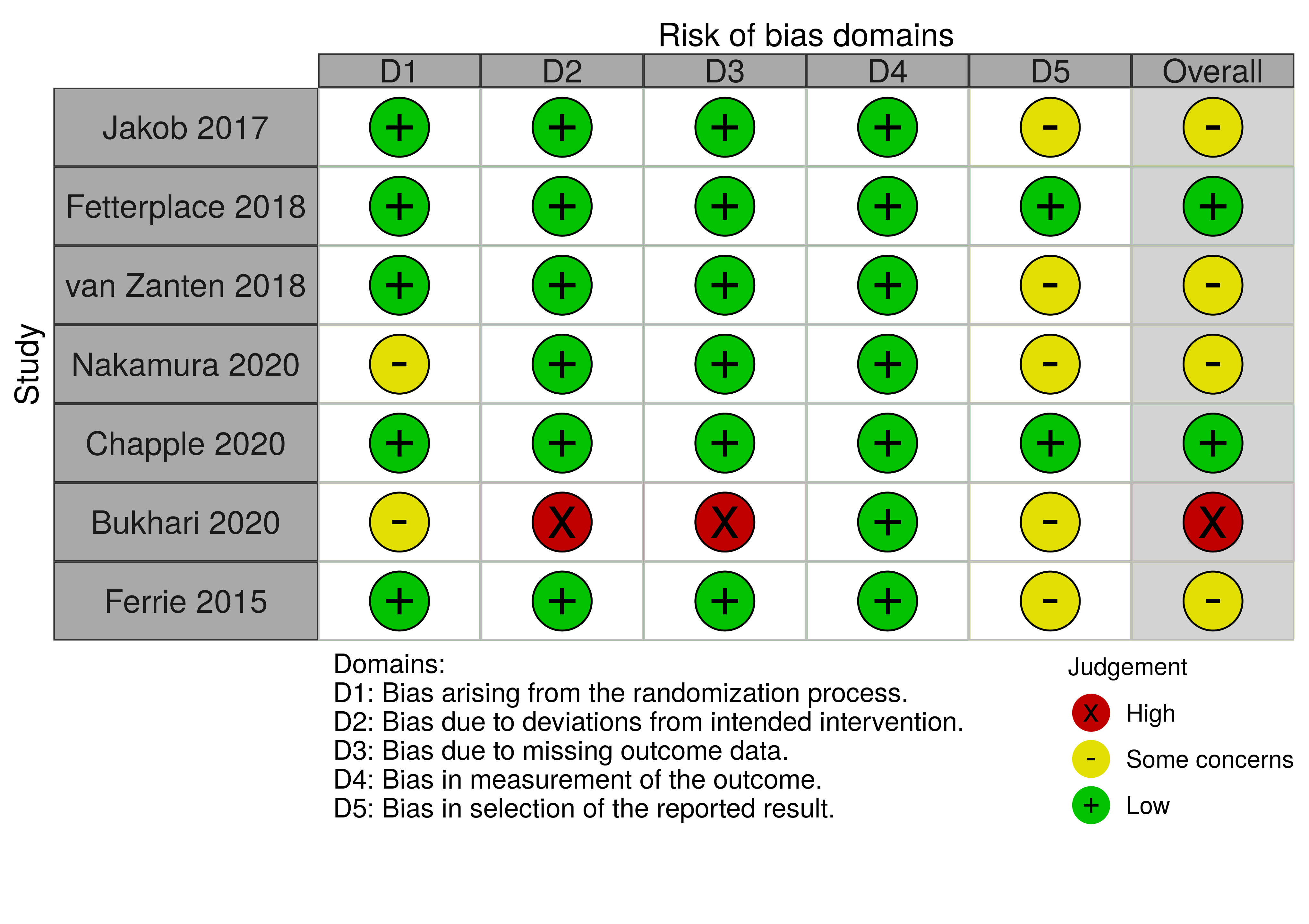** | | **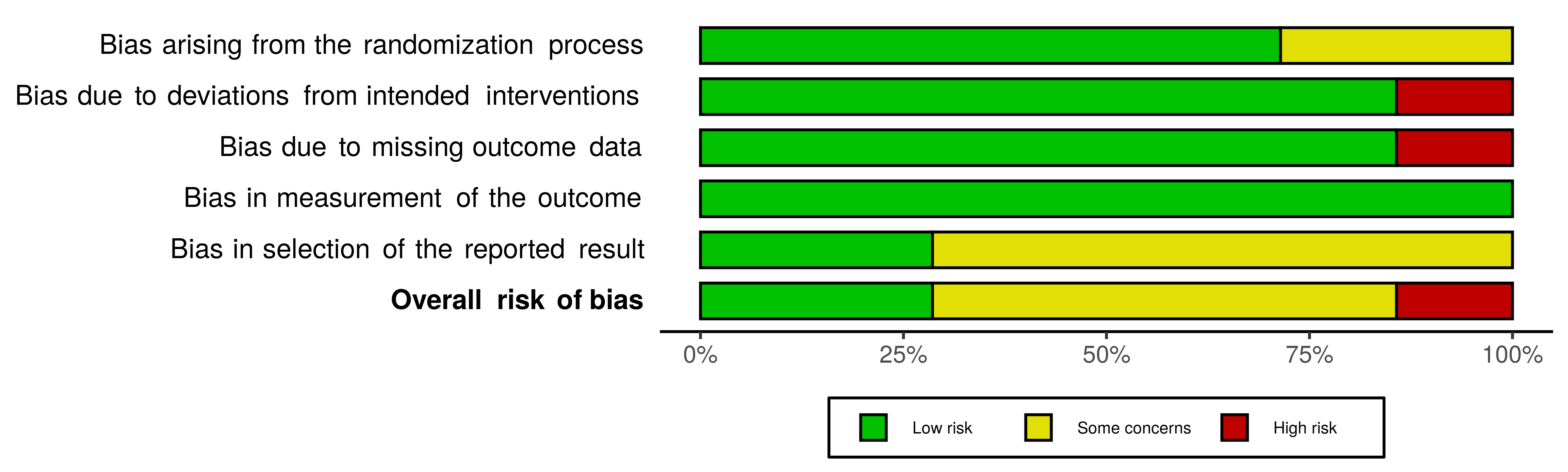** |
| **j) Muscle mass** | | |
| **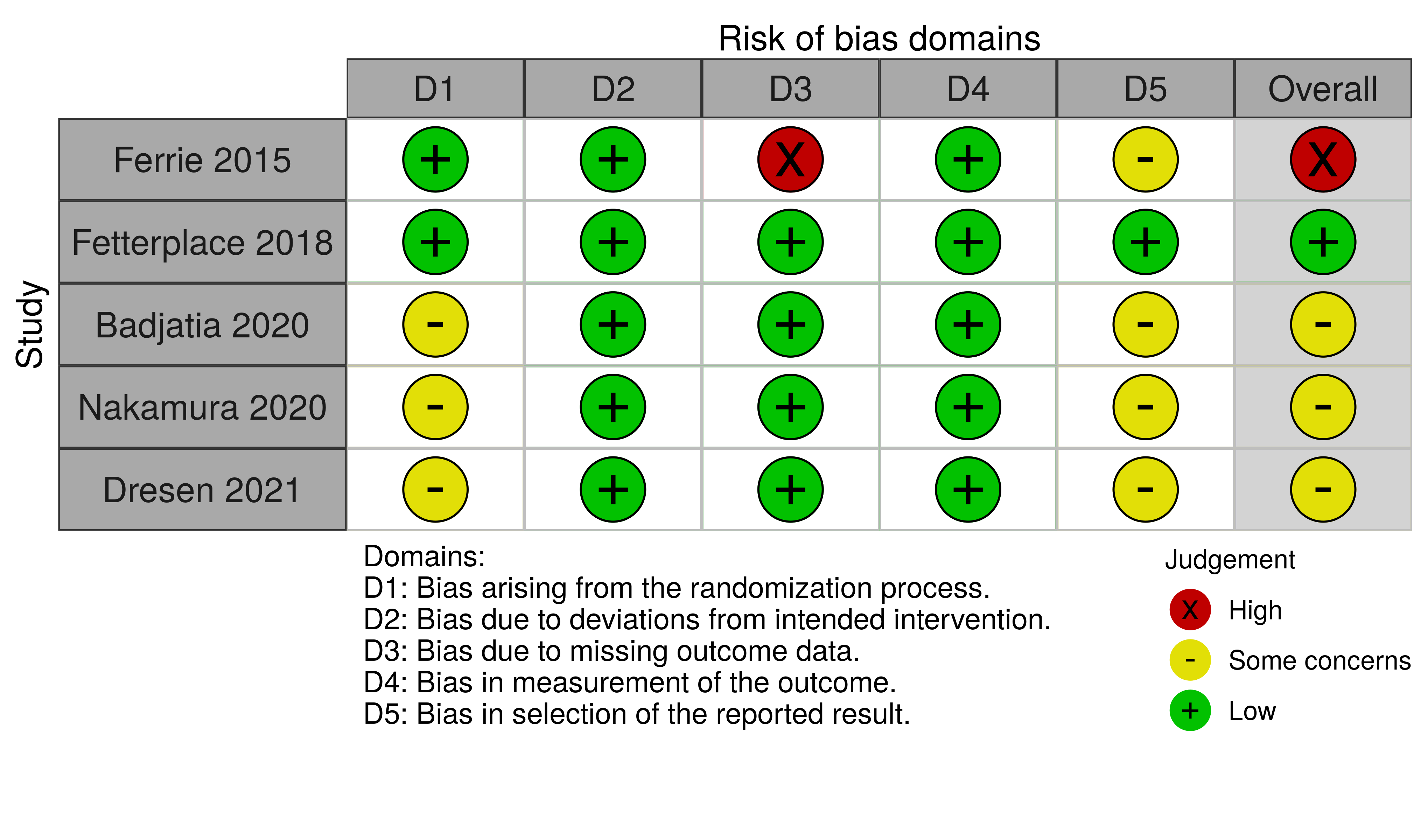** | | **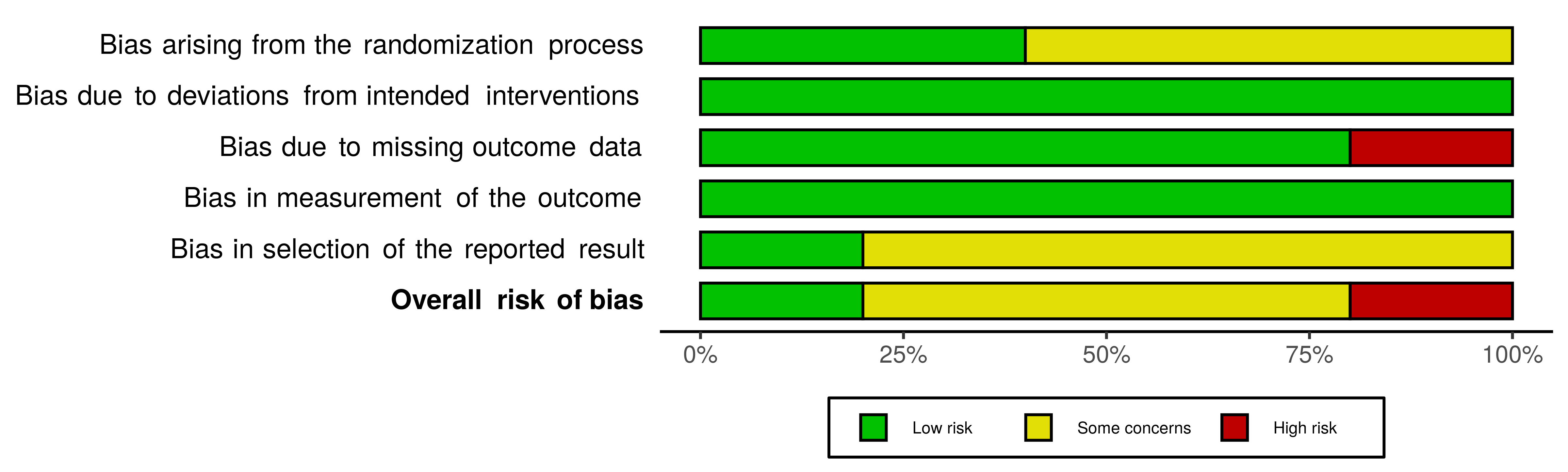** |
| **k) Muscle strength** | | |
| **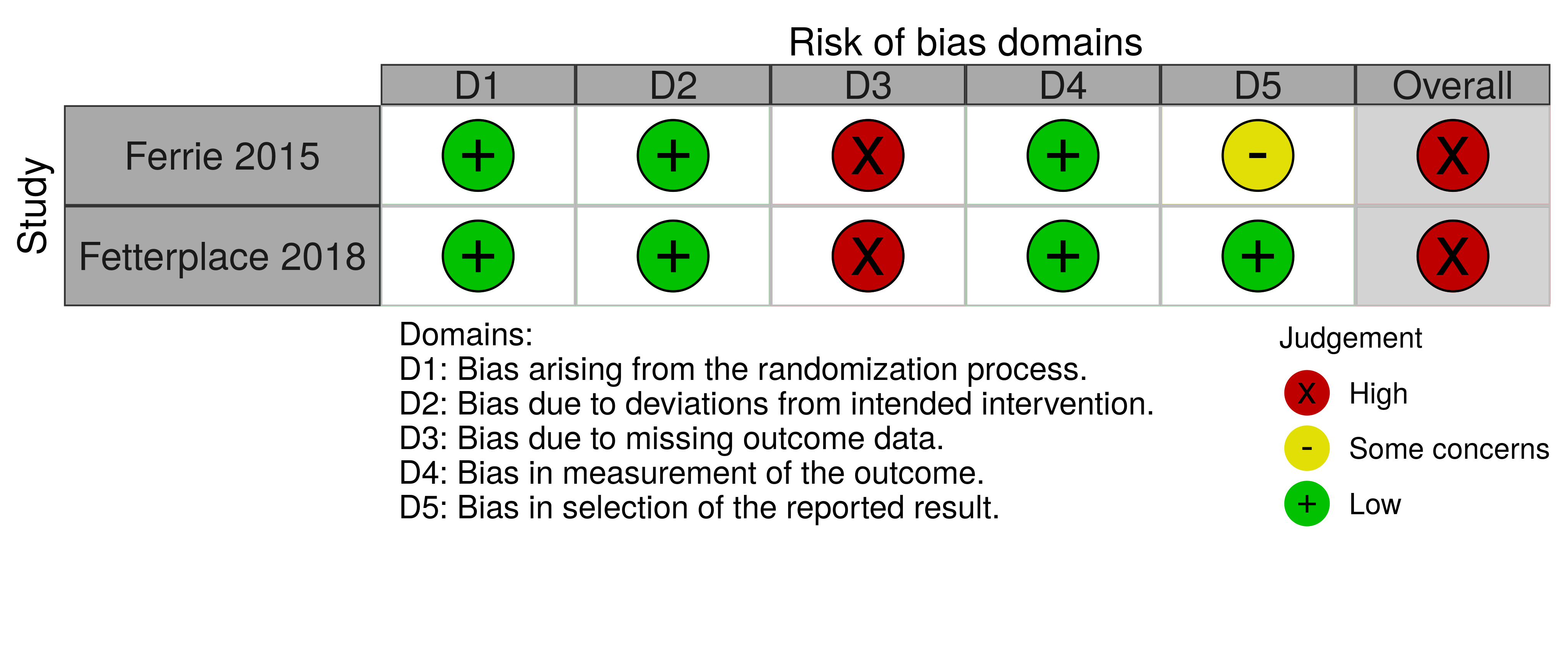** | | **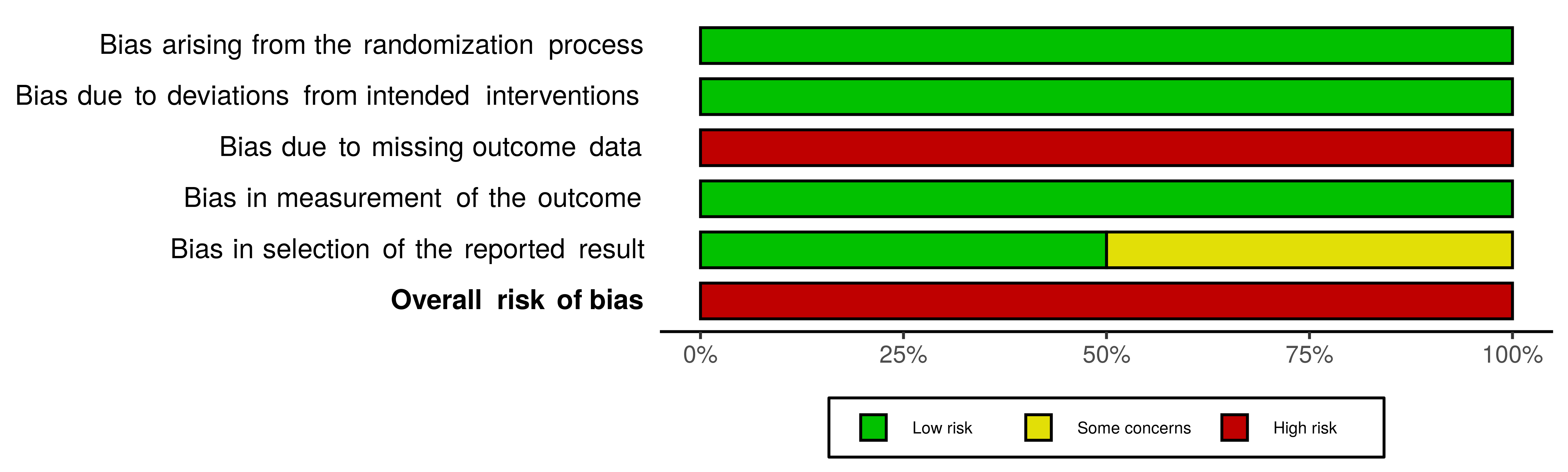** |
| **l) Discharge to rehab facility** | | |
| **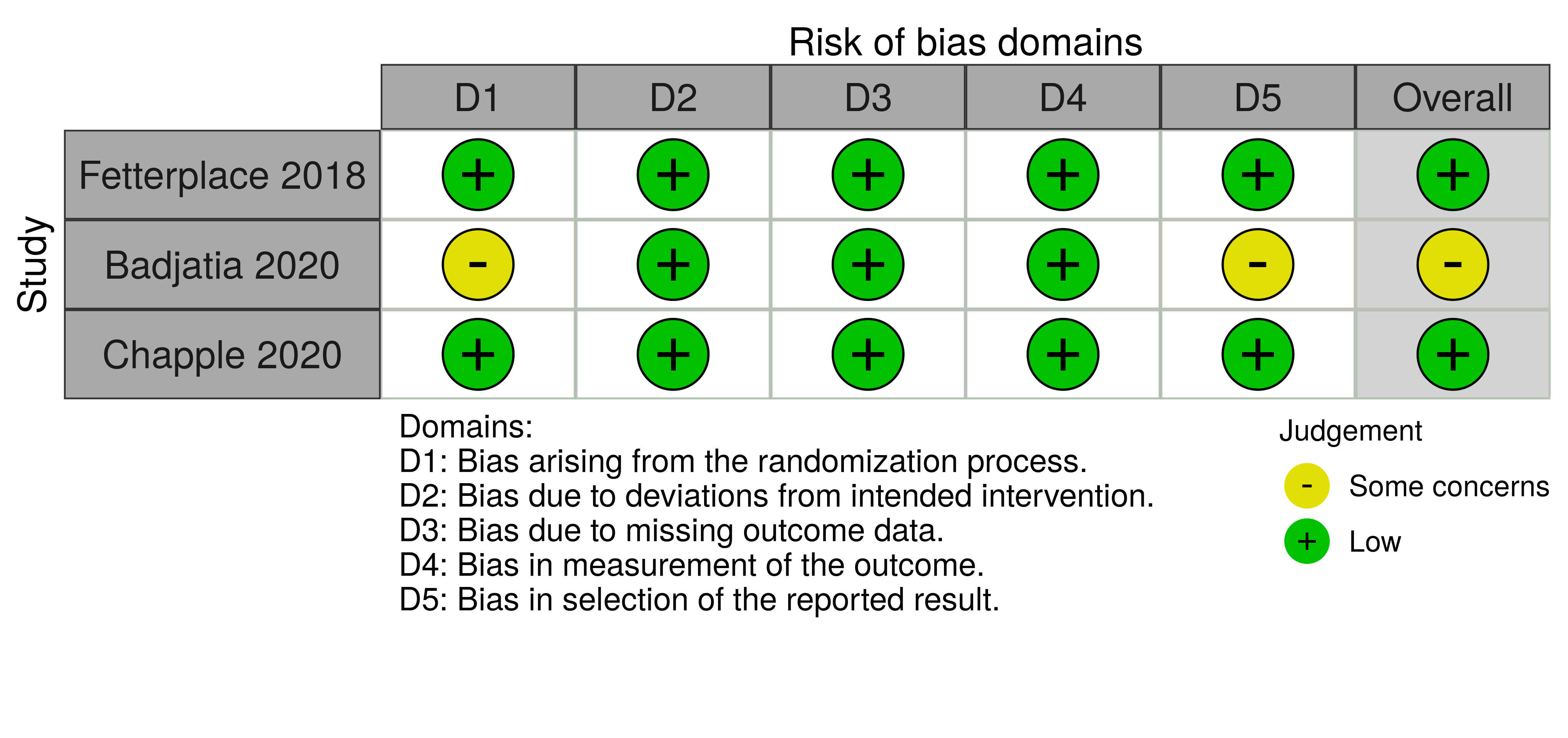** | | **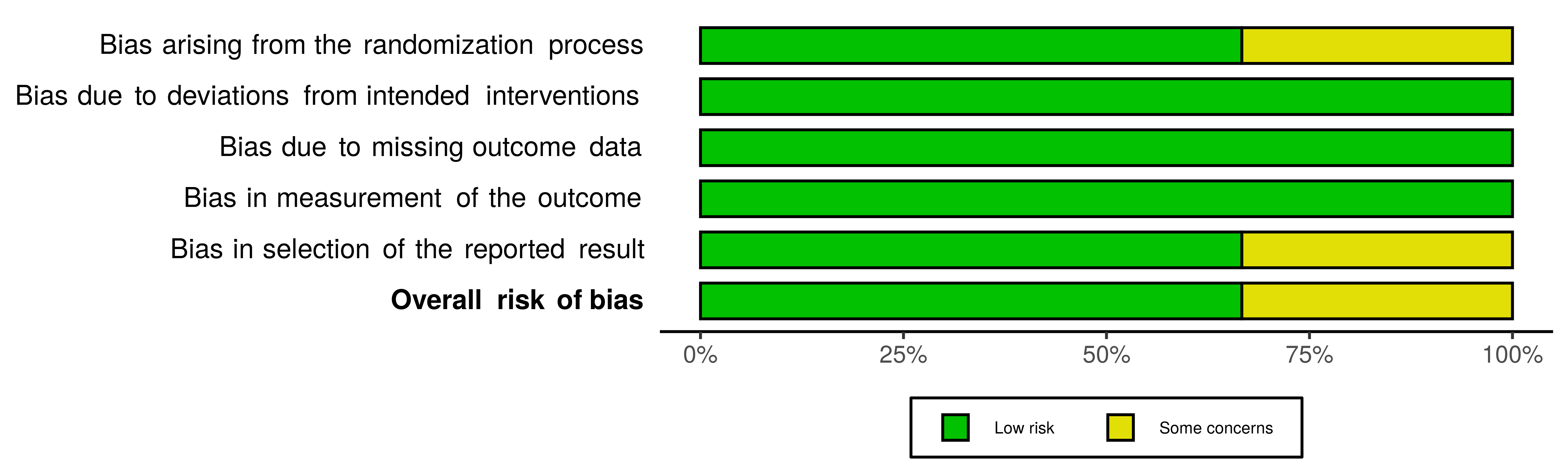** |
| **m) Quality of life physical measures** | | |
| **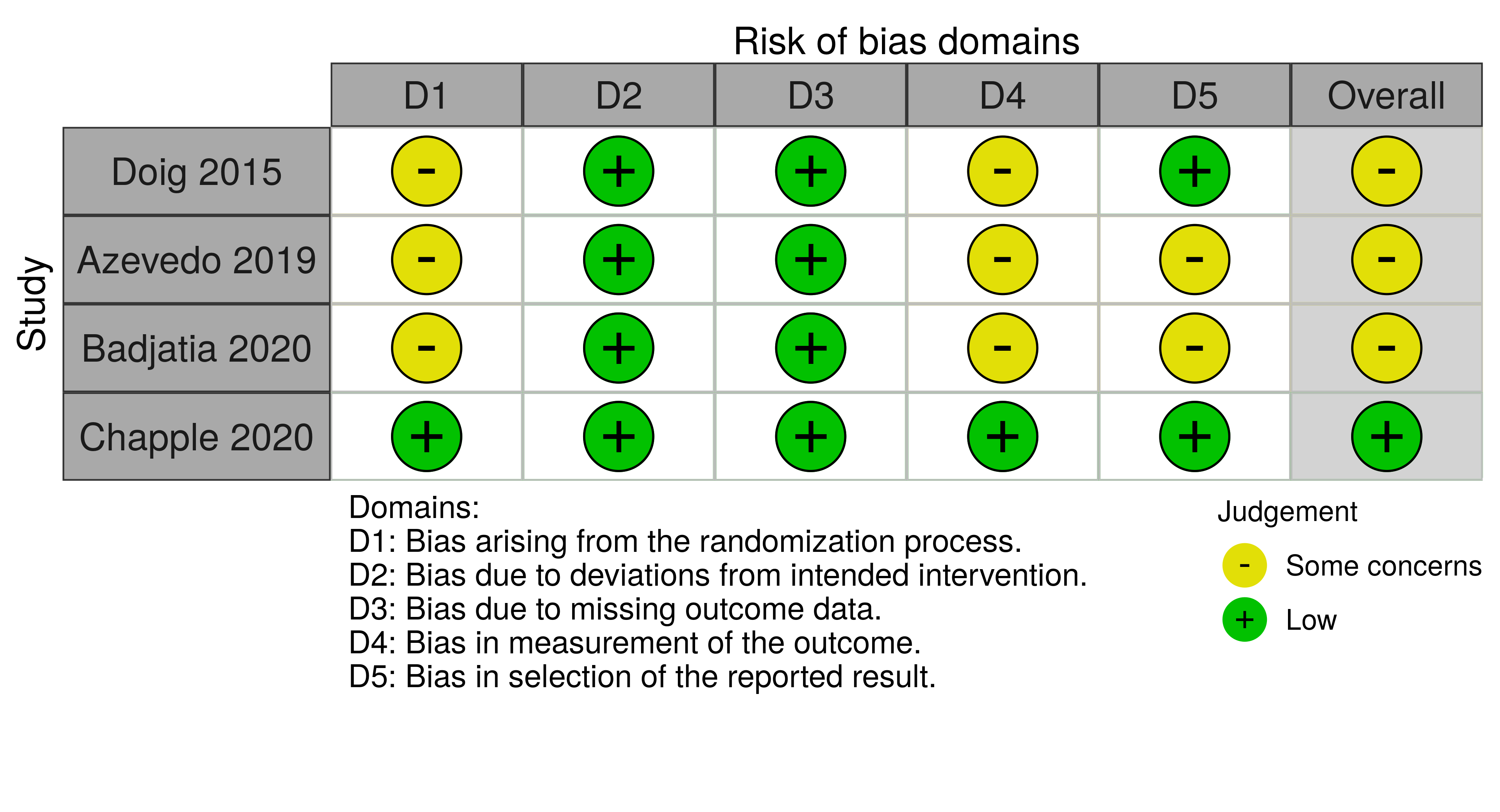** | | **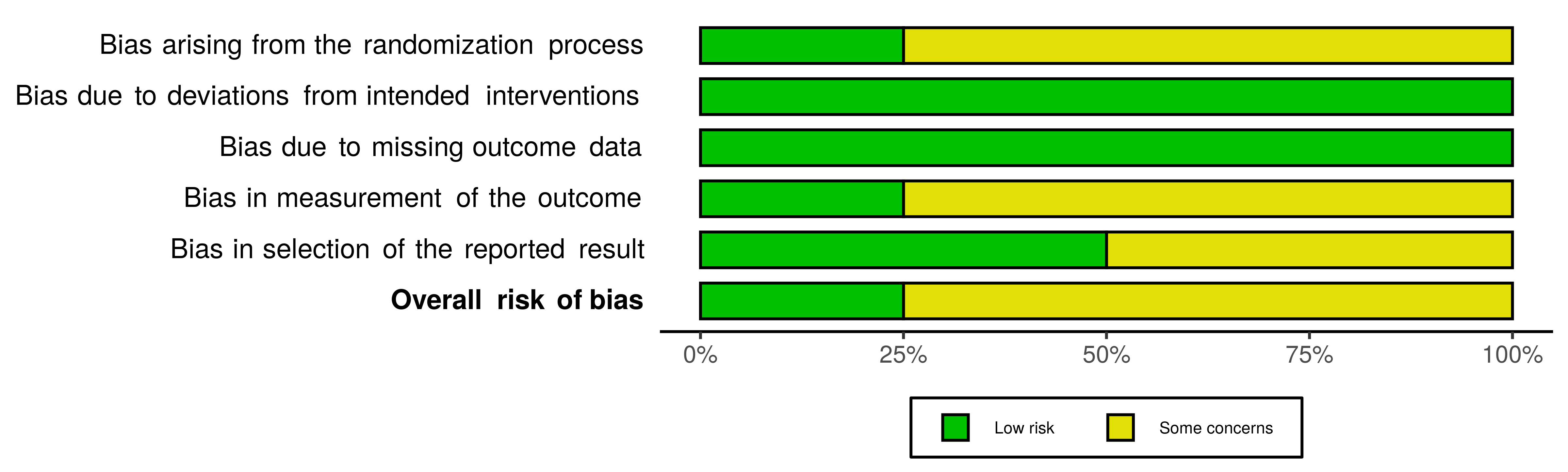** |

**Figure S4 ROB2 Traffic Light and Summary Plots for all Outcomes**

| **a) ICU Mortality**  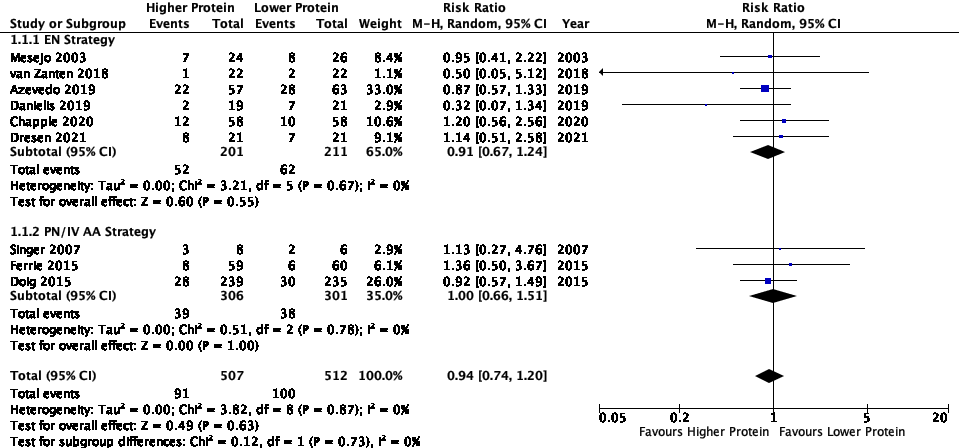 |
| --- |
| **b) Hospital Mortality**  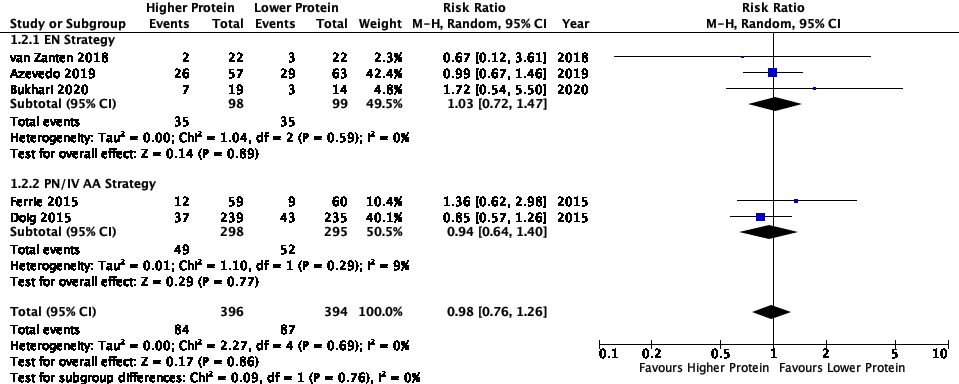 |
| **c) 28-day Mortality**  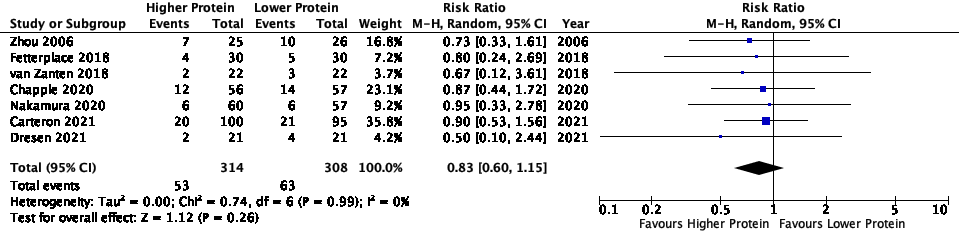 |
| **d) ≥60-day Mortality**  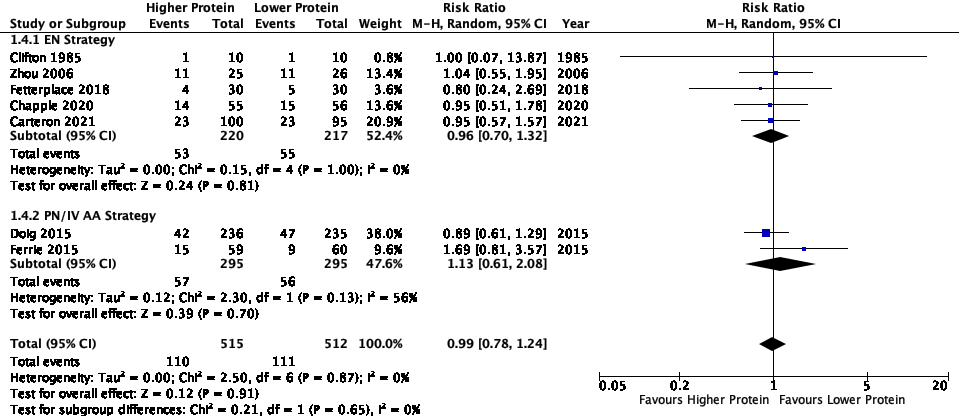 |

**Figure S5 ICU, Hospital, 28- and ≥60-day Mortality**

| **a) Protein Delivered (g/kg/day)**  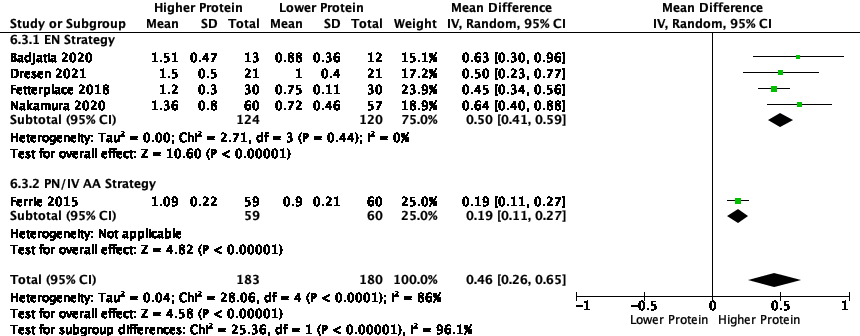 |
| --- |
| **b) Energy delivered (kcal/kg/day)**  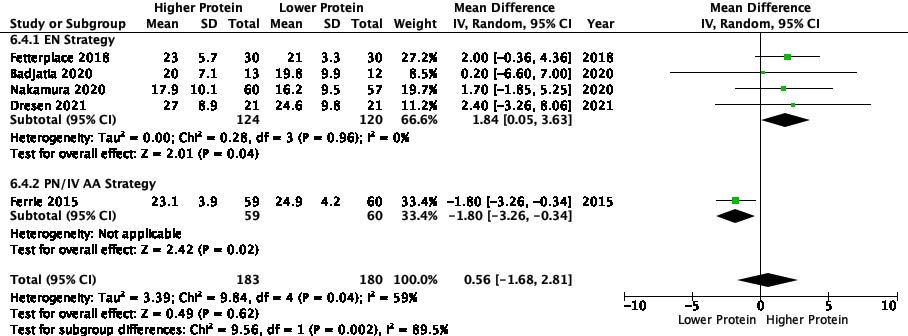 |

**Figure S6 Protein and Energy Delivered in Studies that reported Muscle Outcomes**


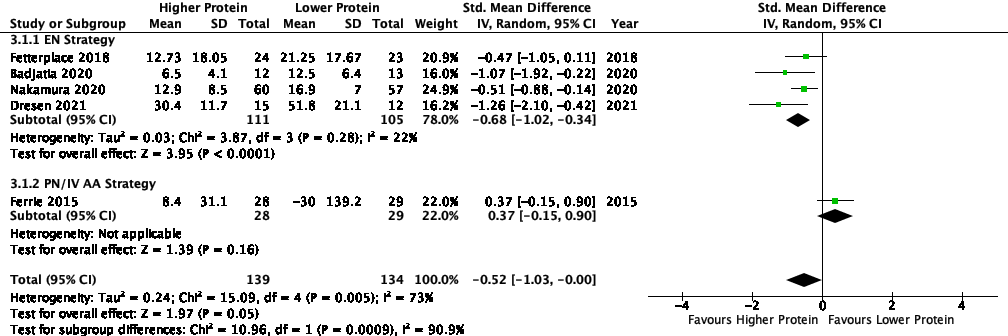


**Figure S7 Percentage of Muscle Change (Standardized)**

Note: Ferrie 2015 found that the lower protein group gained 30.0±139.2% and higher protein group loss 8.4±31.1% of quadriceps muscle. Hence, the sign of the lower protein group was changed to negative to reflect the gain in muscle.


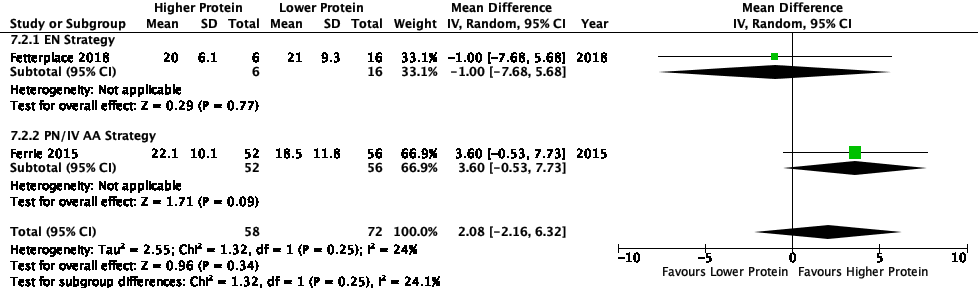


**Figure S8 Handgrip Strength**

Note: Fetterplace 2018: the best handgrip strength at awakening, ICU discharge, or day 15, Ferrie 2015: handgrip strength at day 7. Unable to analyze handgrip strength from Azevedo 2019 because median was reported.


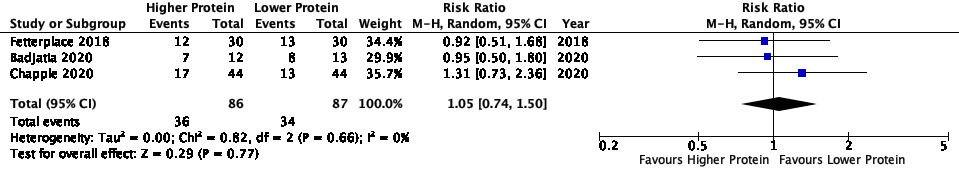


**Figure S9 Discharge to Rehabilitation Facility**


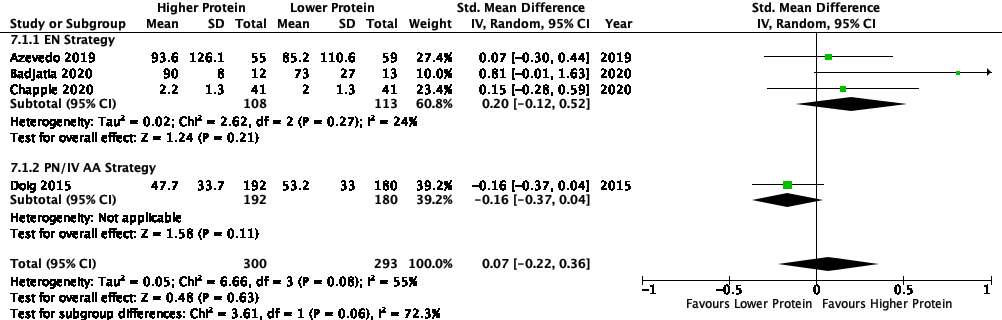


**Figure S10 Quality of Life Physical Measures (Standardized)**

Note:

1. The quality of life (QOL) outcomes reported by the studies were: Doig 2015: RAND-36 general health and physical function at day 90; Azevedo 2019: SF-36 physical component summary (PCS) score at 3- and 6-month; Badjatia 2010: fatigue, lower extremity mobility and cognition outcomes based on the Neuro-QoL questionnaires administered on post-bleed day 90; Chapple 2020: EQ-5D-5L score for mobility, self-care, usual activities, pain/discomfort, anxiety/depression and the result of the EQ-5D-5L visual analogue scale, all at day 90 (see Table S7).
2. The meta-analysis was performed for QOL results associated with physical function: RAND-36 physical function (Doig 2015), SF-36 PCS score at 3-month (Azevedo 2019), Neuro-QoL lower extremity mobility (Badjatia 2010), and EQ-5D-5L score for mobility (Chapple 2020).

| **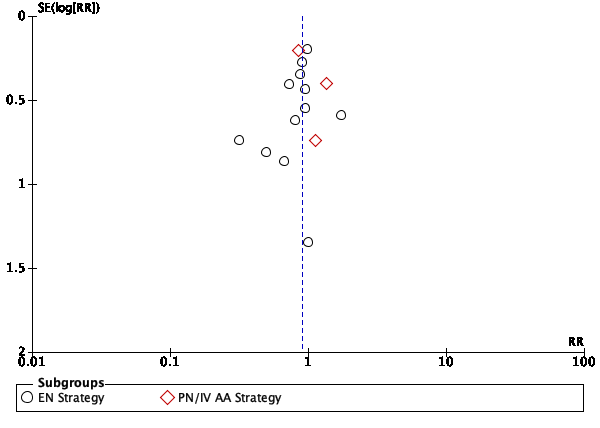**  **a) Overall mortality** (Egger’s test t = -0.0976, df = 13, p = 0.9237) |
| --- |
| **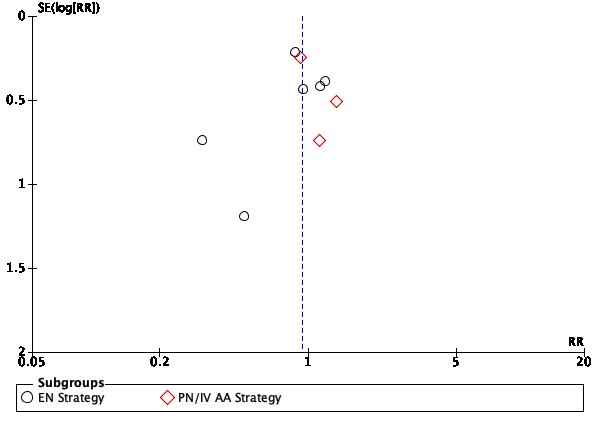**  **b) ICU mortality** |
| 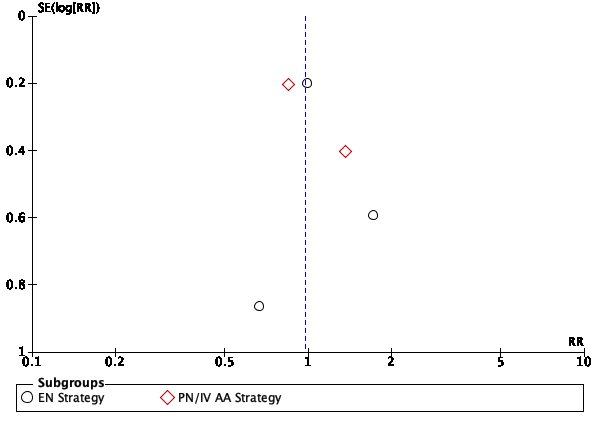  **c) Hospital mortality** |
| 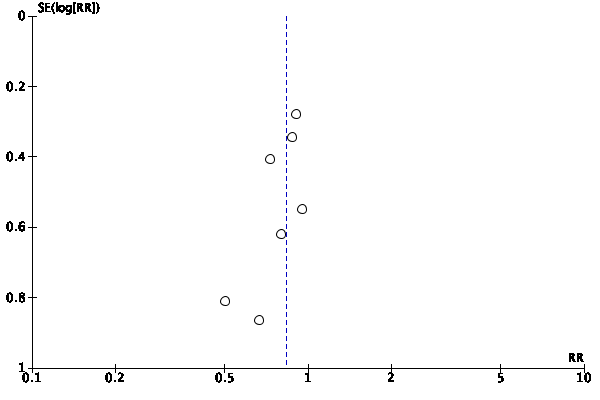  **d) 28-day mortality** |
| 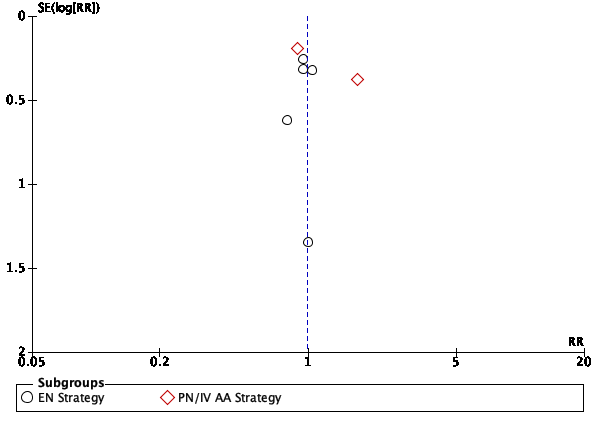  **e) ≥60-day mortality** |
| **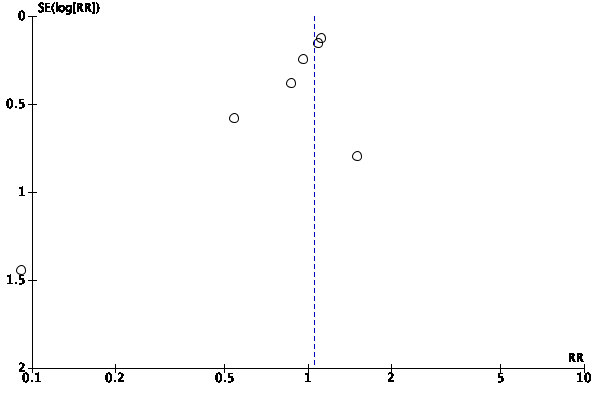**  **f) Infectious complications** |
| **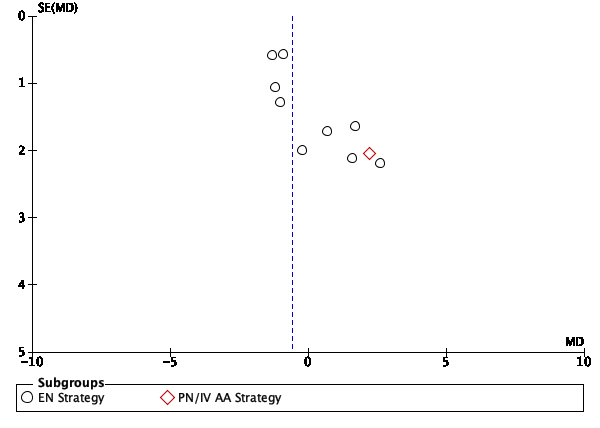**  **g) Duration of mechanical ventilation** (Egger test: t = 4.2813, df = 8, p = 0.0027) |
| **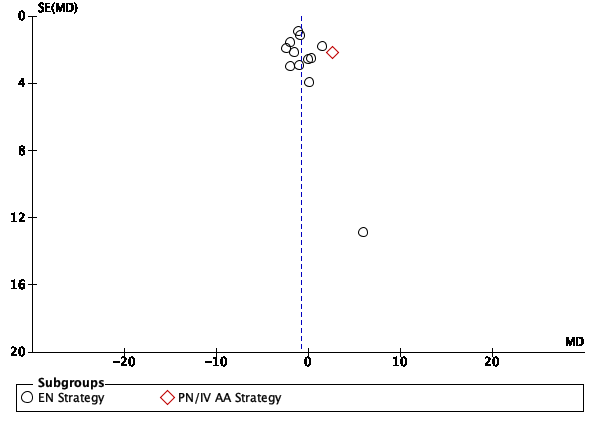**  **h) ICU length of stay** (Egger test: t = 1.0855, df = 11, p = 0.3009) |
| 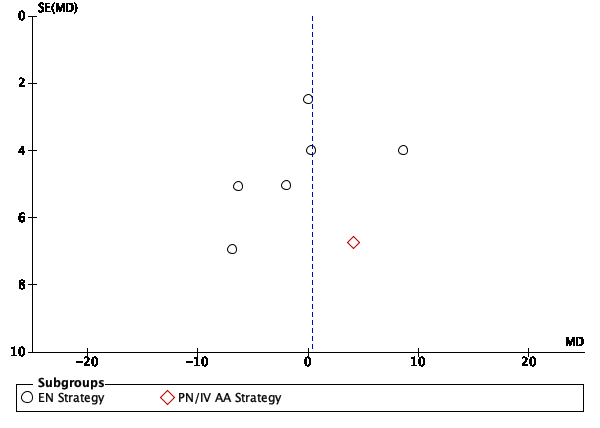  **i) Hospital length of stay** |
| 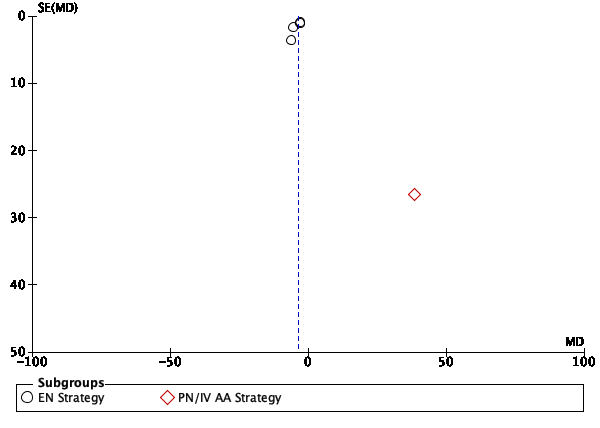  **j) (i) Muscle mass (mean difference)** |
| 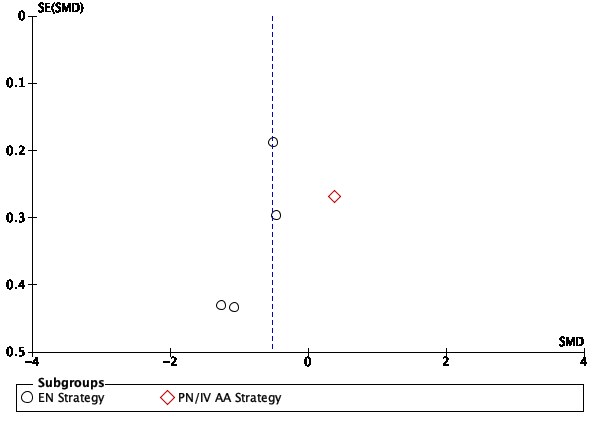  **j) (ii) Muscle mass (standardized)** |
| 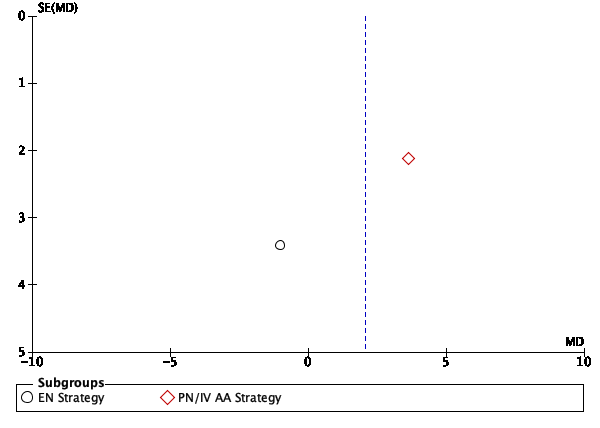  **k) Handgrip strength** |
| 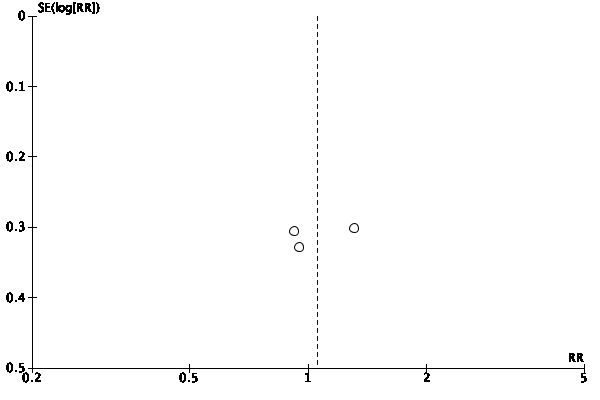  **l) Discharge to rehabiliation facilities** |
| 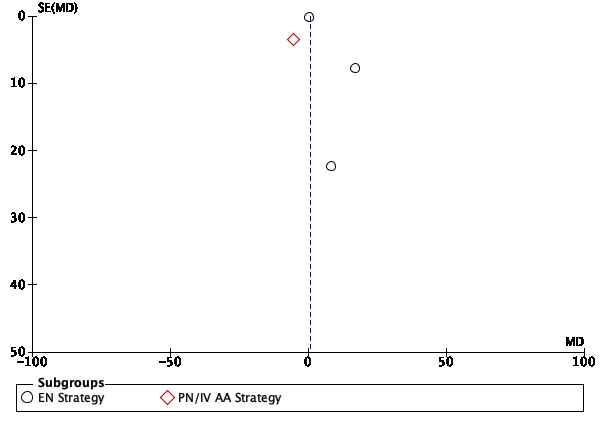  **m) Quality of life physical measure** |

**Figure S11 Funnel plots**
